# Chemoenzymatic synthesis of sulfur-linked sugar polymers as heparanase inhibitors

**DOI:** 10.1038/s41467-022-34788-3

**Published:** 2022-12-02

**Authors:** Peng He, Xing Zhang, Ke Xia, Dixy E. Green, Sultan Baytas, Yongmei Xu, Truong Pham, Jian Liu, Fuming Zhang, Andrew Almond, Robert J. Linhardt, Paul L. DeAngelis

**Affiliations:** 1 Center for Biotechnology and Interdisciplinary Studies, Rensselaer Polytechnic Institute, 110 8th St., Troy, NY 12180 USA; 2 School of Food Science and Pharmaceutical Engineering, Nanjing Normal University, Wenyuan Road 1, Nanjing, 210023 China; 3 Department of Biochemistry and Molecular Biology, University of Oklahoma Health Sciences Center, 940 Stanton L. Young Blvd., Oklahoma, OK 73104 USA; 4 Department of Pharmaceutical Chemistry, Faculty of Pharmacy, Gazi University, 06330 Ankara, Turkey; 5 Division of Chemical Biology and Medicinal Chemistry, Eshelman School of Pharmacy, University of North Carolina, Chapel Hill, North Carolina 27599 USA; 6 School of Chemistry, Manchester Institute of Biotechnology, The University of Manchester, Manchester, M1 7DN United Kingdom

**Keywords:** Glycobiology, Biotechnology, Polysaccharides, Solution-state NMR, Structure-based drug design

## Abstract

Complex carbohydrates (glycans) are major players in all organisms due to their structural, energy, and communication roles. This last essential role involves interacting and/or signaling through a plethora of glycan-binding proteins. The design and synthesis of glycans as potential drug candidates that selectively alter or perturb metabolic processes is challenging. Here we describe the first reported sulfur-linked polysaccharides with potentially altered conformational state(s) that are recalcitrant to digestion by heparanase, an enzyme important in human health and disease. An artificial sugar donor with a sulfhydryl functionality is synthesized and enzymatically incorporated into polysaccharide chains utilizing heparosan synthase. Used alone, this donor adds a single thio-sugar onto the termini of nascent chains. Surprisingly, in chain co-polymerization reactions with a second donor, this thiol-terminated heparosan also serves as an acceptor to form an unnatural thio-glycosidic bond (‘*S*-link’) between sugar residues in place of a natural ‘*O*-linked’ bond. *S*-linked heparan sulfate analogs are not cleaved by human heparanase. Furthermore, the analogs act as competitive inhibitors with > ~200-fold higher potency than expected; as a rationale, molecular dynamic simulations suggest that the *S*-link polymer conformations mimic aspects of the transition state. Our analogs form the basis for future cancer therapeutics and modulators of protein/sugar interactions.

## Abstract (web summary)

Heparin is a family of complex carbohydrates binding to proteins to modulate cell activities. Here the authors report the synthesis, and conformations simulations of *S*-linked hemi-A heparosan [GlcA-S-GlcNAc]_n_, a thio-glycosidic uncleavable polysaccharide, and test it as human heparanase inhibitor.

## Introduction

Glycosaminoglycans (GAGs) are linear, negatively charged heteropolysaccharides that are essential components of the extracellular matrix and contribute to their biological and biomechanical properties^1^. One of the most studied classes of GAGs is heparin/heparan sulfate (HS) with backbones containing disaccharide repeats comprised of (i) a hexosamine residue, glucosamine (GlcN) with an *N*-acetyl (Ac) or *N*-sulfo group, and (ii) a uronic acid residue, either glucuronic acid (GlcA) or iduronic acid^2^. The specific biological roles of GAGs, such as modulating cell-cell interactions, enzyme activity, and cell proliferation during various processes are related to their backbone structure, postpolymerization modifications (e.g., position-specific sulfation, epimerization), chain size, and their cellular localization^3^.

GAGs are biosynthesized by the stepwise transfer of the monosaccharide units from uridine diphosphate (UDP) sugar donors to a saccharidic acceptor^4,5^. In the animal and microbial worlds, the two glycosyltransferases (a GlcA-tase and a GlcNAc-tase) required to biosynthesize the disaccharide repeating units of GAGs are often fused forming a bifunctional enzyme termed a synthase. A variety of GAG synthases have been discovered with distinct primary sequences, topological architectures, and catalytic properties^4^. In the past, we have found that the two synthases of the Gram-negative microbe *Pasteurella multocida* are robust in vitro catalysts capable of synthesizing heparosan ([→4)-α-D-GlcA-(1→4)-β-D-GlcNAc(1→]_*n*_), the unsulfated biosynthetic precursor to HS and heparin in animals. In these recombinant chemoenzymatic systems, unsulfated GAGs can be synthesized as long quasi-monodisperse chains with narrow size distributions of up to MDa sizes or defined oligosaccharides; these methods can also incorporate certain artificial sugar analogs with different functionalities on their pyranose rings^4,6^.

Thioglycosides, in which the oxygen of the glycosidic linkage is replaced with sulfur, are more catabolically stable than their natural *O*-glycoside counterparts due to much lower rates of both acid-catalyzed and enzymatic hydrolysis, making these good candidates as glycosidase inhibitors^7–10^. The prototypical example is the non-hydrolyzable *lac* operon transcriptional inducer, isopropyl thiogalactoside (IPTG), a simple monosaccharide-containing compound synthesized using organic chemistry and extensively applied in recombinant protein expression.

The C-O-C and C-S-C bond lengths and angles differ, and conformational studies on various thio-oligosaccharides show that a *S*-linkage provides a high degree of flexibility between glycosyl units, suggesting these glycans that can easily change their conformation to enable a better fit in the catalytic site when interacting with proteins^11^. Various chemical and enzymatic approaches have been developed to synthesize simple thioglycosides. Organic chemical synthesis of *S*-linked octasialic acid installed an α-*S*-glycosidic bond^12^. A β−1,4-galactosyltransferase was reported to be an efficient chemoenzymatic biocatalyst to prepare thio-linked disaccharide using a synthetic thiol-containing acceptor and a natural UDP-sugar donor^13^. During the submission stage of our work, another group reported the organic chemical synthesis of a disaccharide analog of the heparan sulfate with an *S*-linkage, GlcA-*S*-GlcNAc, sulfated at the C6 position^14^. This molecule was reported to be a competitive inhibitor of human heparanase with IC_50_ (concentration for 50% inhibition) of 15 µM.

In some of our previous efforts to prepare modified sugar nucleotides as potential substrates for glycosyltransferases, we reported two UDP-monosaccharide donors, UDP-4-fluoro (F) GlcNAc^15^ and UDP-4-azido (N_3_) GlcNAc^16^. Their successful enzymatic incorporation into GAGs, affording unnatural GAG analogs having defined sequence length, demonstrated that sugar nucleotide analogs are very useful tools to access functionalized GAGs. However, these analogs are terminators that were not polymerized into larger chains; the artificial functionality on the sugar ring (e.g., F, N_3_) installed on the terminus on a growing chain (the acceptor) cannot substitute for the naturally occurring hydroxyl group that normally attacks the incoming UDP-sugar (the donor) during extension of the nascent GAG polymer. In early 2022, UDP-6-SH-GlcNAc (6-thiol group) was synthesized and tested with the PgaCD enzyme that polymerizes the poly(1,6-β-GlcNAc) bacterial biofilm polymer but this artificial donor was a chain terminator, thus it was also not useful for forming thio-glycosidic (*S*-link) bonds in polysaccharides^17^.

**Fig. 1:**
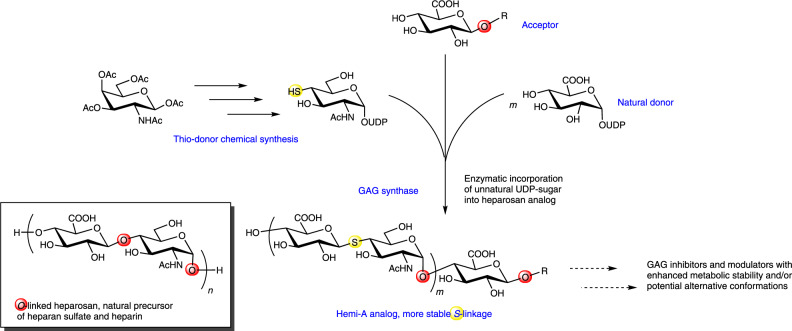
Enzymatic incorporation of 4-SH-*N*-acetylglucosamine into the growing heparosan backbone to form a *S*-linked heparosan extension. The heparosan synthase uses two sugar donors, UDP-GlcNAc or its 4-thiol analog and UDP-GlcA, to create the disaccharide repeats of the GAG backbone chain. (Yellow or red highlight boxes highlight critical sulfur or oxygen atoms in the glycosidic linkages, respectively; *n* = 1 to 2; *m* ~45; R = H, GlcNAc-GlcA-C_2_H_4_-NH_2_, GlcNAc-GlcA-C_2_H_4_-amido-benzaldehyde, or GlcNAc-GlcA-C_2_H_4_-thioamido-fluorescein as noted in Methods). Inset depicts the natural *O*-linked heparosan repeat for comparison.

In this study, we report the synthesis of UDP-4-SH-GlcNAc (4-thiol group) and test its ability to mimic one of the natural donor substrates, UDP-GlcNAc, required in the polymerization of heparosan (the unsulfated precursor of HS and heparin). We hypothesise that this thiol-sugar-containing UDP-donor would be utilized by synthases, affording heparosan chains with a non-reducing terminal 4-SH-GlcNAc, and that its thiol group would be useful in chain labeling or conjugation. Hydroxyl and sulfhydryl functionalities can both serve as nucleophiles, therefore, we also speculate that these 4-SH-GlcNAc-terminated heparosan chains might serve as substrates for the polymerization of heparosan polysaccharides and oligosaccharides with unnatural thio-glycosidic bonds in heparosan’s backbone GlcA-GlcNAc linkages (Fig. 1). Furthermore, we predict that certain *S*-linked HS analogs, after appropriate post-polymerization modification (e.g., *O*- and/or *N*-sulfation), would represent a distinct class of competitive inhibitors for the catabolic enzyme that processes HS in animals, heparanase, an important therapeutic target for cancer treatment^18,19^. Here we describe *S*-linked hemi-A heparosan [GlcA-*S*-GlcNAc]_*n*_, one of the three possible *S*-linked repeating structures (see Methods for discussion on all analogs), which is specifically targeted as a potential heparanase inhibitor.

## Results and discussion

### Synthesis of 4-thiol-*N*-acetyl-D-glucosamine uridine diphosphate (UDP-4-SH-GlcNAc)

**Fig. 2:**
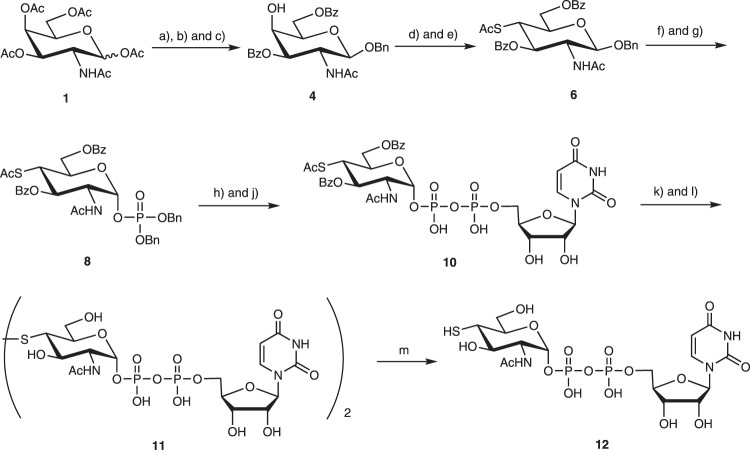
Chemical synthesis of the unnatural donor, UDP-4-SH-GlcNAc 12. Reagents and conditions: **a**) benzyl alcohol, ytterbium(III) trifluoromethanesulfonate, dichloromethane (DCM), reflux 10 h, to form intermediate **2**; **b**) sodium methoxide (NaOMe), methanol (MeOH), room temperature (RT) 12 h, to form intermediate **3**; **c**) Benzoyl chloride, pyridine (Py), -40°C 8 h, 71% for three steps; **d**) trifluoromethanesulfonic anhydride, Py, -10 °C, to form reactive intermediate **5**; **e**) potassium thioacetate, dimethylformamide, RT, 64% for two steps; **f**) FeCl_3,_ DCM, RT, to form reactive intermediate **7**; **g**) tetrabenzyl pyrophosphate, lithium diisopropylamide, tetrahydrofuran, -78°C, 66% for two steps; **h**) Pd/C, H_2_, MeOH, room temperature, to form reactive intermediate **9**; **j**) UMP-morpholidate, Tetrazole, Py, room temperature 3 d, 41% for two steps; **k**) NaOMe, MeOH, RT; **l**) O_2_, RT, 3 days, 52% for two steps; **m**) dithiothreitol in H_2_O, room temperature, 92%. Experimental details are in the Supplementary Information and the analytical data is in Supplementary Fig. 1–37. The reactive intermediate **5**, **7**, and **9** were used in the next step without purification (structures of intermediates **2**, **3**, **5**, **7** and **9** in Supplementary Fig. 1).

The synthesis of 4-SH-GlcNAc was initiated from commercially available D-galactosamine pentaacetate **1** (Fig. 2). A series of steps with excellent selectivity afforded the target benzoate-protected 4-*S*-Ac-*N*-acetyl-D-glucosamine-1-phosphate **9** in good yields (overall 27% for 8 steps). GlmU uridyltransferase was reported to be intolerant of any functional group on the C4 position larger than a hydroxyl group when forming UDP-sugar from its corresponding monophosphate^16,20^. Therefore, we turned to a chemical approach to prepare the unnatural UDP-sugar donor **12**. The protected sugar **8** was debenzylated with hydrogen gas to afford the protected sugar phosphate **9**, which then was converted to the pyridinium salt and reacted with UMP-morpholidate and tetrazole in pyridine for 3 days to afford modest yields (45%) of the protected UDP derivative **10**. Deprotection of **10** was carried out under standard Zemplén conditions followed by treatment with oxygen gas to afford the disulfide derivative **11**, which is more stable and easier to purify than UDP-4-SH-GlcNAc **12**. Incubation of disulfide **11** with the reductant dithiothreitol (DTT) in water afforded the corresponding donor UDP-4-SH-GlcNAc **12**.

### Chemoenzymatic synthesis of *S*-linked analogs

Mass spectrometry (MS)-based assays were used to monitor the incorporation of 4-SH-GlcNAc into heparosan analogs. Catalysis by various *Pasteurella* heparosan synthases (including PmHS1 or PmHS2 as well as chimeric constructs composed of segments of both isozymes^21^) were assessed in two types of reaction: (i) single sugar addition reactions with a GlcA-terminated oligosaccharide acceptor and either natural UDP-GlcNAc or synthetic UDP-4-SH-GlcNAc **12**, or (ii) polymerization reactions that were identical to those in (*i*) but also contained UDP-GlcA, the other sugar donor required to form the disaccharide repeating unit, and either UDP-hexosamine UDP-GlcNAc or UDP-4-SH-GlcNAc **12** with acceptor. The reaction mixtures were analyzed for the presence of elongated species by matrix-assisted laser desorption ionization-time of flight (MALDI-ToF) MS.

Donor sugar 4-SH-GlcNAc was successfully incorporated into a GlcA-terminated oligosaccharide acceptor (R’) to form the target heparosan with a free thiol group at the nonreducing end of the chain as evidenced by the additional mass from the sulfur atom (Table 1, GlcNAc-R’; Supplementary Fig. 38a). The only other unique chemical modification site on a natural GAG molecule is at the opposite end of the polymer chain, the reducing terminus, where a hemiacetal (aldehyde functionality) allows selective orthogonal reactivity without extraneous coupling events. The other GAG functionalities, carboxyl and hydroxyl groups, are present at multiple locations along the chain, thus, their selective singular modification is not possible. The new thiol group, introduced at the non-reducing terminus, allows for coupling with sulfhydryl-reactive reagents (e.g., maleimide, iodoacetate). A chain terminated with a 4-SH-GlcNAc residue can be employed for the facile construction of uniquely tagged GAG probes or in drug delivery conjugates.

**Table 1:** Mass spectral analysis of native and thio-linked hemi-A heparosan

Product	GlcNAc-R’	(GlcNAc-GlcA)_1_-GlcNAc-R’	(GlcNAc-GlcA)_2_-GlcNAc-R’
**Native (= 4-OH)**			
*Observed*	950.38 ± 0.11	1,329.50 ± 0.04	1,708.65 ± 0.05
*Predicted*	950.29	1,329.40	1,708.51
**4-SH Analog**			
*Observed*	966.29 ± 0.11	1,361.43 ± 0.05	1,756.56 ± 0.06
*Predicted*	966.27	1,361.36	1,756.44
Observed ∆ S-O	15.91	31.93	47.91

The observed and predicted masses (Da) are shown. The mass difference between the natural *O*-linked and the artificial *S*-linked species (last row of table) corresponds to the additional mass (~16 Da per S atom) associated with the replacement of oxygen with 1, 2, or 3 sulfur atoms (acceptor R’ = GlcA-GlcNAc-GlcA-C_2_H_4_-amido-benzaldehyde).

Furthermore, unlike the 4-F-GlcNAc or 4-N_3_-GlcNAc derivatives^15,16^ that are chain terminators in GAG biosynthesis, the 4-SH-GlcNAc-modified heparosan can serve as an acceptor during co-polymerization to form new thioglycosidic bonds. This exciting observation is demonstrated by the additional mass (~16 Da/disaccharide) in these thio-heparosan analogs in comparison to the natural, *O*-linked heparosan polysaccharide indicates that the sulfur atom is used in the new glycosidic linkages formed between GlcA and GlcNAc residues (Table 1, (GlcNAc-GlcA)_1-2_-GlcNAc-R’; Supplementary Fig. 38b). PAGE analysis also confirmed the formation of the new products in co-polymerization reactions (Supplementary Fig. 39).

The unnatural 4-SH-GlcNAc residue was slowly and inefficiently incorporated by the heparosan synthases (~0.8% incorporation compared to the natural GlcNAc). However, when the polymerization reactions took place over extended periods of time and these reactions were scaled-up, useful amounts of target *S*-linked polymer could be produced. A recombinant chimeric synthase, PmHS-B^21^, was better at analog incorporation than the two natural-sequence PmHS isozymes or other catalyst constructs. We explored many other reaction parameters (e.g., buffer pH, metal ions, temperature, donor/acceptor ratios; *see* Supplementary Information) to optimize incorporation with only a modest enhancement of product yield. Therefore, future improvements may require more highly engineered synthases capable of better accommodating and/or utilizing thiol-donors. The current *S*-linked heparosan analog had an average molecular weight of ~18 kDa (range ~13–22 kDa or ~65-110 monosaccharide units, Supplementary Fig. 40), a size quite useful for exploring the many heparin/HS bioactivities and these products are well beyond the range of simple thioglycosides and oligosaccharides reported in the literature^11–14^.

There are three possible *S*-linked heparosan repeating structures assuming that both 4-SH-UDP-sugar donors were available. Two species are possible with a single thio-glycosidic linkage, hemi-‘A’ GlcA-*S*-GlcNAc and hemi-‘B’ GlcNAc-*S*-GlcA, as well as one where both are thio-glycosidic linkages. Here we describe only the *S*-linked hemi-A heparosan which was specifically targeted as a potential heparanase inhibitor.

The *O*-linkage in *S*-linked hemi-A heparosan, GlcNAc-*O*-GlcA, can still be cleaved by bacterial heparin lyases (see below; Supplementary Fig. 39), suggesting that the *S*-linked hemi-A heparosan has a sufficiently similar overall structure (primary and secondary) to that of natural heparosan and heparin/HS GAGs^22,23^. Heparin lyase treatment of *S*-linked hemi-A heparosan and natural heparosan followed by disaccharide analysis using LC-MS^24^ showed 2-aminoacridone (AMAC)-labeled single disaccharide products of *m/z* 588 and *m/z* 572, respectively (Supplementary Fig. 41), corresponding to AMAC-labeled ΔUA-*S*-GlcNAc and ΔUA-*O*-GlcNAc (where ΔUA is 4-deoxy-α-L-*threo*-hex-4-enopyranosyluronic acid resulting from lyase-catalyzed β-elimination). These data are consistent with the structure of *S*-linked hemi-A heparosan shown in Fig. 1. One- and two-dimensional NMR analyses confirmed the structure of the *S*-linked hemi-A heparosan showing chemical shifts characteristic of the structural differences in the linkage region (^1^H 4.40 vs. 4.48 ppm and ^13^C 102.4 vs. 85.4 ppm for natural heparosan and *S*-linked hemi-A heparosan, respectively (Supplementary Fig. 42–48).

Based on reports that other thio-glycosidic linkages were resistant to glycosidase digestion, we hypothesized that the *S*-linked hemi-A heparosan once appropriately converted to an *S*-linked heparin/HS analog through sulfation would not be degradable by human heparanase. This enzyme is an endo-β-glucuronidase that cleaves the GlcA-*O*-GlcNAc linkage in heparin/HS. In the case of *S*-linked heparin/HS analogs, this would now be an unnatural thio-glycosidic (GlcA-*S*-GlcNAc) linkage forming the basis for a competitive inhibitor.

**Fig. 3:**
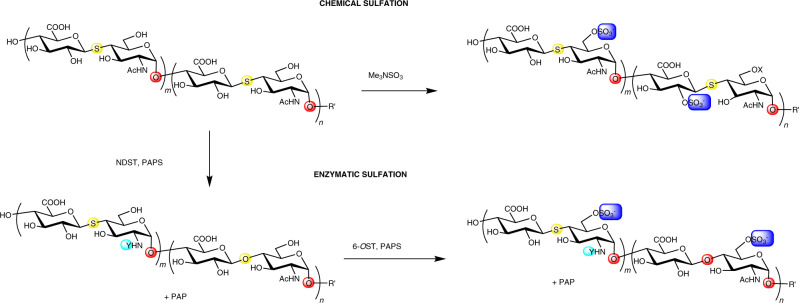
Synthesis of sulfo-4-thio-HS analogs. The starting hemi-A thio-heparosan analog (where *m* = ~45 and *n* = 0 or 1; R’ = H or GlcA-C_2_H_4_-thioamido-fluorescein) contains a hemi-A *S*-linked structure (boxed). Chemical *O*-sulfation (top pathway) results in 6-*O*-sulfo GlcNAc or 2-*O*-sulfo GlcA residues. Enzymatic *N*- and 6-*O*-sulfation, catalyzed by NDST and 6-OST using the sulfo-donor 3′-phosphoadenosine-5′-phosphosulfate (PAPS) (bottom pathway), results primarily in 6-*O*-sulfo and *N*S, 6-*O*-sulfo GlcNAc residues. (Highlighted atoms of the major structures: S, yellow; O, red; Y = acetyl or sulfo, aqua; X = sulfo or hydroxyl, blue. Experimental details and sulfation isomer levels are provided in the Supplementary Information).

Chemical sulfation of the *S*-linked hemi-A heparosan using sulfur trioxide-trimethyl amine complex in basic aqueous solution afforded an *O*-sulfated-4-thio-heparosan analog (Fig. 3). Following heparin lyase treatment, disaccharide analysis by LC-MS/MS demonstrates the presence of 2-*O*-sulfo-ΔUA-*S*-GlcNAc and ΔUA-*S*-6-*O*-sulfo-GlcNAc (Supplementary Fig. 49, 50). In a parallel synthesis, we examined whether *S*-linked hemi-A heparosan could be enzymatically converted to the *N-*sulfated-4-thio-HS analog using *N*-deacetylase, *N*-sulfotransferase (NDST) (Fig. 3). Disaccharide analyses showed that the two-step treatment with HS NDST and HS 6-O-sulfotransferase (6-OST) converted the GlcNAc residues into GlcN-sulfo and GlcN-*N*S,6-*O*S residues thus afforded the *N*- and *O*-sulfated-4-thio-HS analogs (Supplementary Fig. 51, 52). The *S*-linked hemi-A, *N*-sulfo and 6-*O*-sulfo HS analog afforded a disaccharide analysis that closely resemble porcine kidney HS (Supplementary Fig. 53). The chemically and enzymatically sulfated *S*-link HS analogs both possess the 6-*O*S-GlcNAc modification, a structure that appears to be recognized by human heparanase.

### *S*-linked analog: enzyme challenges and heparanase inhibition

**Fig. 4:**
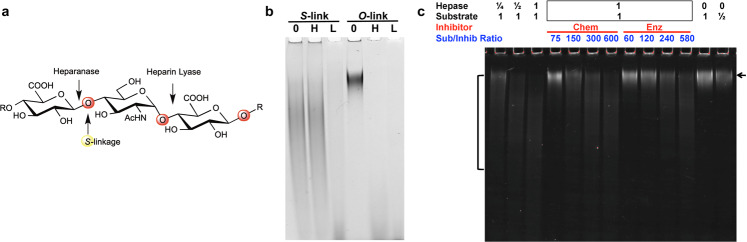
O-sulfo-S-linked heparosan analog enzyme challenges and inhibition. **a** Cleavage sites of the heparosan chain by the human heparanase or the bacterial heparin lyase enzymes, and the position of the sulfur atom substitution in the hemi-A linkage (for the various pendant sulfo-positions in the sulfated products, see Fig. 3). **b** The chemically *O*-sulfated *S*-link analog or natural *O*-link heparosan (**0**, starting polymers without enzyme) were tested for cleavage by overnight treatment with either the recombinant human heparanase (**H**) or a mixture of recombinant *Flavobacterium* heparin lyases I, II, and III (**L**) (*n* = 1; also see Supplementary Fig. 54 for an independent heparanase challenge). PAGE analysis (10% gel) with Stains-All detection showed that the *S*-link polymer is resistant to heparanase digestion at its thio-glycosidic linkage GlcA-*S*-GlcNAc sites but cleaved by the lyases at the GlcNAc-*O*-GlcA sites with natural glycosidic linkages. As expected, both types of enzymes can digest the natural glycosidic linkages of *O*-sulfo-*O*-linked HS. **c** PAGE analysis (20% gel) of the kinetics (20-min point) of heparanase enzyme (**Hepase**) cleavage of a fluorescent sulfo-*O*-linked heparosan substrate (starting material marked with arrow) with a titration of a *S*-linked analog inhibitor at decreasing concentrations from left to right (either chemically, Chem, or enzymatically, Enz, sulfated polymer; Sub/inhib, substrate molecules:inhibitor molecule molar ratio) (one of 7 titrations shown, each with 2 timepoints and 2 gels). Reaction controls: one quarter or half of the enzyme (2 leftmost lanes; the bracket denotes the approximate extent of fragments with ½× enzyme); half of the substrate without enzyme (rightmost lane).

All natural *O*-linked and unnatural *S*-linked hemi-A heparosan-based polymers (including *O*-sulfo, *N*-sulfo/6-*O*-sulfo HS analogs) were bacterial heparin lyase sensitive as expected (Supplementary Fig. 39, 41, 49–51). In contrast, recombinant human heparanase only cleaved the *O*-sulfo*-O*-linked heparosan and natural HS, but not the *O*-sulfo*-S*-linked analog (Fig. 4b, Supplementary Fig. 54). Furthermore, co-incubation of a sulfo-*S*-linked HS analog with the *O*-sulfo-*O*-linked heparosan inhibited the digestion of the *O*-linked substrate (Fig. 4c, Supplementary Fig. 55). Similarly, the *O*-sulfo*-S*-linked polymer acted as an inhibitor protecting natural HS from human heparanase digestion (Supplementary Fig. 56b), thus demonstrating that this analog acts as a competitive heparanase inhibitor with potential oncology applications. Excitingly, the sulfated *S*-linked hemi-A HS analogs were at least ~200- to 300-fold more active on a molar basis than the structurally comparable *O*-linked substrate (i.e. only difference is the substitution of the oxygen with a sulfur atom) based on its IC_50_ in the 5–20 nM range. This finding suggests tighter heparanase binding to the *S*-linked analogs than polysaccharides with only natural *O*-linkages. For comparison to a known heparanase inhibitor, the commercially available compound suramin was tested in our heparanase assay yielding an IC_50_ of ~0.5 µM (Supplementary Fig. 57), a value in the approximate range of other reports using different substrates and conditions. Therefore, the sulfated *S*-linked polymers are ~100-fold better on a molar basis than the highly sulfonated suramin, which is also known to bind or inhibit many proteins in the body.

### Molecular dynamics of *S*-linked analog conformations

**Fig. 5:**
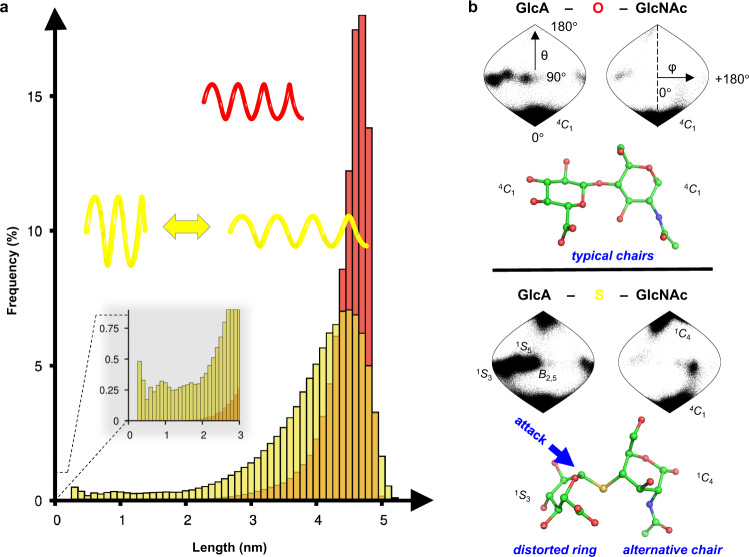
Theoretical polysaccharide chain lengths and ring pucker conformers as predicted by molecular dynamics simulations. **a** Histograms show the end-to-end length relationship of decasaccharide models of mono-6*OS*-sulfated *O*-linked (*red*) chains or *S*-linked (yellow) hemi-A HS chains (see Supplementary Fig. 58 for other sulfation isomers) as a function of frequency (~500,000 data points/run). The cartoon depicts the wider range of predicted conformations exhibited by the *S*-linked HS analog showing both collapse (inset) and extension like a Slinky®. **b** The hypothetical pucker conformational states of the most interior GlcA ring and GlcNAc ring of the decasaccharide models for *O*- versus *S*-linked heparosan chains (unsulfated polymers here; sulfated versions in the SI) are shown as sinusoidal plots. The azimuthal angle θ discriminates boats from chairs (θ = 0° is a perfect ^4^*C*_1_-chair and is at the south pole), while the meridional angle φ determines the pseudo-rotation of the boat conformations (in this definition φ = 0° represents the ^O,3^*B*-boat). Both rings of natural *O*-link chains typically reside in the chair conformation ^*4*^*C*_*1*_ state, but the rings of *S*-link chains are predicted to occupy alternative states more frequently (see Supplementary Fig. 61 for other details). One hypothetical disaccharide conformer in the hemi-A *S*-linked unit, ^*1*^*S*_*3*_−^*1*^*C*_*4*_, depicts a GlcA sugar with a distorted ring that is observed in some enzyme active sites and an axial glycosidic bond; the enzyme’s nucleophile could potentially attack this target scissile bond with optimal in-line geometry. However, the *S*-link is uncleavable thus results in competitive inhibition of the enzyme.

The *S*-linked HS analogs have different bond characteristics than the natural polysaccharide species (C-O-C ~116° angle, C-O ~0.14 nm length versus C-S-C ~99° angle, C-S ~0.178 nm length)^25^ that can potentially alter the polysaccharide solution conformation. We employed multi-µs explicit aqueous molecular dynamics simulations of decasaccharides (models of the polysaccharide) to investigate the theoretical structural differences spawned by the *S*-linkage in various heparosan chains with hemi-A structures compared to chains with all-natural *O*-links. The *S*-linked chain conformation is predicted to be floppier and can extend to greater lengths than the natural polymers, but the analog also has a great tendency to collapse (Fig. 5a, Supplementary Fig. 58) in a similar fashion to the Slinky® helical spring toy that can expand, bend and contract over a wide range.

Interestingly, the dynamic exchange of puckering of the sugar rings (i.e. 38 possible pyranose conformations including chairs, half-chairs, envelopes, skews, or boats^26^) captured on µs-timescale simulations was predicted to be very different for *O*-linked and hemi-A *S*-linked polysaccharides (Fig. 5b, Supplementary Fig. 59, 60, 62). The sulfation of the chains had some influence on the molecular dynamics, but the main driver of this unusual behavior was due to the *S*-links. Typically, both the GlcA and GlcNAc rings in natural *O*-linked heparosan chains are predicted to primarily adopt the low-energy ^*4*^*C*_*1*_ chair conformers. On the other hand, the GlcNAc rings of the *S*-linked analog are predicted to be much more likely to traverse the energy barrier and adopt the alternative chair ^*1*^*C*_*4*_(~40–85% vs ~0.01% for *S*- vs *O*-link, respectively).

Furthermore, the pyranose ring of the GlcA unit connected by hemi-A *S*-links (especially in chains with the 6-*O*S modification) is predicted to more readily adopt several interconvertible skew/boat conformations (primarily ^*1*^*S*_*5*_, ^*1*^*S*_*3*_, ^*1,4*^*B*, *B*_*2,5*_) in comparison to the *O*-linked chains (typically ~26–29% vs ~2.6-4.6% frequencies for this class of conformers in *S*- vs *O*-linked, respectively; Fig. 5b, Supplementary Fig. 59, 60, Supplementary Tables 1–4). Such distorted rings are known to occur in the transition states of glycosidases (the shorter C1-O5 distances, longer C1-O1 distances, and a more planar geometry mimic the postulated oxocarbenium cation mechanistic intermediate^26^. In addition, the glycosidic bond emerging from *S*-linked GlcA residue in these pucker states (as well as the *S*-linked GlcNAc residue ^*1*^*C*_*4*_) occupies a more axial position rather than the typical equatorial position found in the natural chains (Fig. 5b, Supplementary Fig. 61). Therefore, these conformers at the heparanase target scissile bond could align with the catalytic nucleophile in the enzyme active site as found in the canonical retaining glycosidase mechanism^26^. At this time,^13^C-enriched polysaccharide is not available to validate our various simulation-derived conformational hypotheses by NMR. A recent NMR study of a pair of less complex disaccharide mimics of HS concluded that the change from an *O*- to an *S*-linkage led to observable conformational shifts^27^, providing experimental evidence for the exploration of alternative backbone geometries, which is consistent with our modeling on the sugar pucker of *S*-linked polysaccharides.

With respect to the higher-than-expected levels of human heparanase inhibition exhibited by the sulfated *S*-linked polymers, we speculate that the C-S-C bonds spawn increased chain flexibility and/or ring puckering conformers that mimic some aspects of the transition state of the HS chain undergoing cleavage in the heparanase active site^28^. A chain bend is observed in this co-crystal structure, and the very flexible hemi-A heparosan analog chains (Fig. 5a) should be able to easily conform to the active site. Our simulations of pucker also suggest that the *S*-linked chains can mimic the transition state in important ways (i.e., axial linkage position of the target bond, distorted GlcA ring; Fig. 5b), as well as the intrinsically longer C-S-C bonds may resemble a C-O-C bond in the early stages of breaking. It is well known that transition state analogs or distorted structures are better inhibitors than strict substrate structural analogs with unperturbed bonds^11,26^. In comparison to other types of heparanase inhibitors in the clinic^18,29^, the hemi-A *S*-linked polymers appear to be a distinct class of therapeutic candidate that may employ altered conformations to interact favorably with the enzyme. Our *S*-linked polysaccharide is ~3 orders of magnitude more effective on a concentration basis than the recently reported *S*-linked disaccharide analog^14^; potential explanations for this enhancement include the long chains can exhibit higher avidity (i.e., multiple binding sites per chain) and/or bind more extensively to the heparanase active site (at least ~3-4 saccharide units^28^).

In summary, we report the synthesis of the UDP-4-SH-GlcNAc analog using an efficient chemical approach. The sugar from this unnatural donor was incorporated into heparosan using a polysaccharide synthase. Unlike fluoro- or azido-donors, the UDP-4-SH-GlcNAc not only played the role of glycosyl donor, but the resulting 4-SH-GlcNAc residue also performed as an acceptor to further participate in polymerization to form a thio-glycosidic bond. To the best of our knowledge, these are the first reported sulfur-linked polysaccharides. Similar biomacromolecule analogs are the peptide-nucleic acids, where the informational units (e.g., A, T, C, G, U bases) are connected through amide bonds that are recalcitrant to nuclease cleavage, thus, having added benefits compared to natural RNA and DNA chains^30^. Based on this study, it is likely that in the future other and more complicated thio-linked GAG analogs, such as *S*-linked anticoagulant heparin, could be prepared using thiol-modified nucleotide sugars.

*S*-linked polysaccharide structures may either enhance or decrease interactions with various GAG-binding proteins^3,31^, depending on the protein and polysaccharide studied. Exploration of the *S*-linked GAG chemical space promises altered selectivity as the natural HS-based sugars bind to a plethora of proteins (e.g., heparin binds to over 200 different proteins in human plasma) resulting in a multitude of biological activities. The utility of alternative sugar conformations and resistant bonds affords opportunities to expand the possibilities for precise and improved therapeutics.

## Methods

All reagents were purchased from Sigma-Aldrich or other commercial vendors unless otherwise noted and were used without further purification.

### Synthesis and analysis of UDP-4-SH-GlcNAc donor analog

#### General information

Flash chromatography (FC) was performed using silica gel (200 − 400 mesh; Millipore Sigma, Burlington, MA, USA) according to standard protocols. Reactions were monitored by thin-layer chromatography (TLC) on silica gel F254 plates (20 cm × 20 cm, Millipore Sigma. Mass data were acquired by MALDI-ToF-MS (matrix-assisted laser desorption/ionization time of flight mass spectroscopy) or electrospray ionization (ESI)-high-resolution (HR)-MS on an LTQ-Orbitrap XL FT-MS spectrometer ^1^H, ^13^C, ^1^H-^1^H correlation spectroscopy (COSY) and ^1^H-^13^C heteronuclear single quantum coherence (HSQC) nuclear magnetic resonance spectroscopy (NMR) spectra were recorded on an 800 MHz (200 MHz for ^13^C NMR) or a 600 MHz (150 MHz for ^13^C NMR) spectrometer. See Supplementary Methods section for the preparation of compounds **1**–**8**.

#### Synthesis of disodium uridine 5’-(2-acetamido-2,4-dideoxy-4-acetylthio-3,6-di-O-benzoyl-α-D-gluco-pyranosyl) diphosphate (**10**)

The protected phosphate **8** (10 mg, 0.013 mmol) was dissolved in methanol (2 ml). Pd/C (10 wt % on activated carbon) (6 mg) was added and the mixture was stirred under a hydrogen atmosphere for 1 h. The palladium was then filtered off and the solvent was removed *in vacuo* to produce crude **9** in 92% yield, which was used in the next step without further purification.

A solution of monophosphate **9** (7 mg, 0.012 mmol) in methanol (2 ml) was treated with triethylamine (9 µl, 0.07 mmol), and then concentrated to yield crude bis(triethylammonium) phosphate. Without purification, this crude material was repeatedly co-evaporated with dry pyridine (3 × 3 ml). Uridine 5′-monophosphomorpholidate 4-morpholine-*N*,*N*′-dicyclohexylcarboxamidine salt (14 mg, 0.02 mmol) was co-evaporated with pyridine (3 × 3 ml) in a separate vessel, then transferred in 2 ml pyridine through a cannula into the reaction flask. The combined reagents were co-evaporated with pyridine (2 × 2 ml), then 1H-tetrazole (4 mg, 0.05 mmol) in pyridine (2 ml) was added, and the reaction mixture was stirred at room temperature for 3 days. The reaction mixture was then concentrated *in vacuo* and was converted into Na^+^ form by passing through a Dowex (Na^+^) column. The resulting fraction was concentrated in vacuum and the residue was loaded onto a Bio Gel P2 column (1 × 120 cm; BioRad) eluted with water. Fractions were collected, and those containing the product as determined by mass spectrometry were combined and freeze-dried to afford **10** as a white powder (5 mg, 45%). ^1^H NMR (600 MHz, D_2_O) *δ*: 8.01-7.93 (m, 1H, H-Ar), 7.86-7.77 (m, 3H, H-Ar, uridine-H”-6), 7.66-7.54 (m, 2H, H-Ar), 7.52-7.38 (m, 5H, H-Ar), 5.81-5.74 (m, 2H, rib-H’-1, uridine-H”-5), 5.69-5.63 (m, 1H, H-1), 4.73-4.71 (m, 1H, H-6), 4.56 (d, *J* = 11.93, 1H, rib-H’-6), 4.47 (d, *J* = 9.93, 1H, H-2), 4.38 (d, *J* = 11.93, 1H, H-6’), 4.29-4.11 (m, 7H, H-3, H-4, H-5, rib-H’-2, rib-H’-4, rib-H’-3, rib-H’-6’), 2.02 (s, 3H, methyl H-SAc), 1.78 (s, 3H, methyl H-NHAc); ^13^C NMR (150 MHz, D_2_O) *δ* 200.9 (carbonyl C-SAc), 177.8 (carbonyl C-NHAc), 171.3 (carbonyl-C-Bz), 171.0 (carbonyl-C-Bz), 169.2 (rib-C”-4), 154.7 (rib-C”-2), 144.7 (uridine-C”-6), 137.6 (C-Ar), 137.4 (C-Ar), 132.8 (C-Ar), 132.8 (C-Ar), 132.7 (C-Ar), 132.7 (C-Ar), 132.2 (C-Ar), 131.4 (C-Ar), 105.6 (uridine-C”-5), 98.0 (d, *J* = 4.8, C-1), 92.5 (rib-C’-1), 86.2 (rib-C’-5), 77.5 (C-3), 74.8 (C-4), 73.1(rib-C’-2), 72.6 (rib-C’-3), 70.6 (C-5), 69.6 (C-6), 68.6 (rib-C’-5), 56.2 (C-2), 33.1 (methyl C-SAc), 25.1 (methyl C-NHAc).^31^P NMR (243 MHz, D_2_O) *δ* -10.79 (d, *J* = 24.3, 1 P), -13.56 (d, *J* = 24.3, 1 P). ESI-HRMS calcd. for C_33_H_37_N_3_O_19_SP_2_([M - H] ^-^) 872.1144, found 872.1141. (Supplementary Fig. 20–25)

#### Synthesis of uridine 5’-(2-acetamido-2,4-dideoxy-4-thio-α-D-gluco-pyranosyl) diphosphate disulfide (**11**)

Sodium methoxide solution (20 µl, 5.5 M in methanol) was added to a solution of **10** (6 mg, 0.007 mmol) in anhydrous methanol (1 ml) and the resulting solution left to stir overnight. The solution was then neutralized with pre-washed and acidified Amberlyte IR-120 hydrogen ion exchange resin and the reaction mixture filtered through cotton wool to remove the resin. The mixture was stirred under an oxygen atmosphere for 3 days. The reaction mixture was concentrated under reduced pressure and the residue was purified by high-performance liquid chromatography with an YMC-Triart C_18_ column (10 mm × 250 mm, 5 µm), using 50 mM pH 5.5 ammonium acetate aqueous solution. The resulting fraction was concentrated under vacuum and the residue was loaded onto a Bio Gel P2 column (1 × 120 cm) eluted with water to give **11** as a white solid (4.4 mg, 52%). ^1^H NMR (800 MHz, D_2_O) δ 7.88 (d, J = 8.1 Hz, 2H, uridine-H”-6), 5.91 – 5.85 (m, 4H, uridine-H”-5, rib-H’-1), 5.46 (dd, J = 7.3, 3.2 Hz, 2H, H-1), 4.26 (dt, J = 20.9, 5.0 Hz, 4H, rib-H’-2, rib-H’-3), 4.22 – 4.14 (m, 4H, rib-H’-4, rib-H’-5a), 4.13 – 4.08 (m, 2H, rib-H’-5b), 4.04 (dt, J = 11.1, 3.0 Hz, 2H, H-3), 3.96 – 3.90 (m, 4H, H-2, H-5), 3.89 – 3.86 (m, 2H, H-6a), 3.80 (t, J = 10.3 Hz, 2H, H-6b), 2.87 (t, J = 10.9 Hz, 2H, H-4), 1.99 (s, 6H, methyl H-NHAc); ^13^C NMR (200 MHz, D_2_O) δ 174.8 (carbonyl C-NHAc), 166.2 (uridine-C”-4), 151.7 (uridine-C”-2), 141.6 (uridine-C”-6), 102.6 (uridine-C”-5), 94.6 (C-1), 88.7 (rib-C’-1), 82.99 (d, J = 10, rib-C’-4), 73.9 (rib-C’-2), 72.8 (C-3), 69.3 (rib-C’-3), 67.8 (C-2), 64.8 (rib-C’-5), 60.9 (C-6), 54.8 (C-4), 54.2 (C-4), 22.1 (methyl C-NHAc)^31^.P NMR (243 MHz, D_2_O) *δ* -11.43 (d, *J* = 21.9, 1 P), -13.13 (d, *J* = 21.9, 1 P). ESI-HRMS calcd. for C_34_H_52_N_6_O_32_S_2_P_4_([M-H]^-^) 1243.0945, found 1243.0959. (Supplementary Fig. 26–31).

#### Synthesis of uridine 5’-(2-acetamido-2,4-dideoxy-4-thio-α-D-gluco-pyranosyl) diphosphate (**12**)

Dithiothreitol (3 mg) was added to a solution of disulfide **11** (4.4 mg, 0.0035 mmol) in water (1 ml) and the resulting solution was stirred at room temperatrue for 10 min. The reaction mixture was concentrated under reduced pressure and the residue was loaded onto a Superdex 30 Increase 10/300 GL column and eluted with 0.2 M ammonium bicarbonate aqueous solution. The resulting fraction was lyophilized to give **12** as a white solid. (4 mg, 92%). ^1^H NMR (600 MHz, D_2_O) δ: 7.89 (d, J = 8.25, 1H, uridine-H”-6), 5.91-5.86 (m, 2H, uridine-H”-5, rib-H’-1), 5.51-5.45 (m, 1H, H-1), 4.31-4.23 (m, 2H, rib-H’-2, rib-H’-3), 4.22-4.14 (m, 2H, rib-H’-4, rib-H’-5a), 4.14-4.08 (m, 1H, rib-H’-5b), 4.06-4.02 (m, 1H, H-3), 3.96-3.86 (m, 3H, H-2, H-5, H-6a), 3.84-3.78 (m, 1H, H-6b), 2.85 (t, J = 10.87, 1H, H-4), 1.99 (s, 3H, methyl H-NHAc); ^13^C NMR (150 MHz, D_2_O) δ 178.2 (carbonyl C-NHAc), 169.6 (uridine-C”-4), 155.17 (uridine-C”-2), 144.9 (uridine-C”-6), 105.9 (uridine-C”-5), 98.1 (d, *J* = 3, C-1), 92.0 (rib-C’-1), 86.4 (rib-C’-4), 77.1(rib-C’-2), 76.1(C-3), 72.7 (rib-C’-3), 71.1 (d, J = 30, C-2), 68.2 (rib-C’-5) 64.3 (C-6), 58.1 (C-4), 25.4 (methyl C-NHAc). ^31^P NMR (243 MHz, D_2_O) *δ* -11.42 (d, J = 21.9, 1 P), -13.11 (d, J = 21.9, 1 P). ESI-HRMS calcd. for C_17_H_27_N_3_O_16_SP_2_([M-H]^-^) 622.0514, found 622.0510. (Supplementary Fig. 32–37).

### Polysaccharide backbone synthesis

There are three possible *S*-linked heparosan repeating structures assuming that both of the 4-SH-UDP-sugar donors were available (only UDP-4SH-GlcNAc as reported here has been synthesized to our knowledge). Two polymeric species are possible with a single thio-glycosidic linkage, hemi-A GlcA-*S*-GlcNAc and hemi-B GlcNAc-*S*-GlcA, as well as one species where both are thio-glycosidic linkages. Here we describe only the *S*-linked hemi-A heparosan polymer which was specifically targeted as a potential heparanase inhibitor.

#### Demonstration of thio-sugar addition and production of hemi-A S-link heparosan

##### Single sugar addition and polymerization reactions

An acceptor, an aldehyde-containing glycoside of heparosan trisaccharide (GlcA-GlcNAc-GlcA-C_2_H_4_-amido-benzaldehyde^32^; 0.2 mg/ml, 0.27 mM), was incubated with: (i) UDP-hexosamine sugar (either natural UDP-GlcNAc or UDP-4-SH-GlcNAc analog) alone (for single sugar addition), (ii) UDP-hexosamine sugar and UDP-GlcA (for polymerization), or (iii) UDP-GlcA alone (negative control), at 5 molar excess over the acceptor concentration, with a recombinant heparosan synthase chimeric construct, PmHS-G^21^ (2 mg/ml, 18 µM), in 1 mM MnCl_2_, 50 mM 4-(2-hydroxyethyl)-1-piperazineethanesulfonic acid (HEPES) buffer at pH 7.2, for 20 h at 30 °C. For the preparation of UDP-4-SH-GlcNAc monomer, the UDP-thio-sugar donor dimer **11** was first reduced by treatment with 5 mM dithiothreitol in water at 22 °C for 20 min. Reaction products were diluted 1:10 in 50% acetonitrile, 0.1% trifluoroacetic acid, and a portion was mixed in a 1:1 ratio with the matrix solution of 6-aza-2-thiothymine (5 mg/ml in 50% acetonitrile, 0.1% trifluoroacetic acid) and dried with a stream of air. The spot was assessed by matrix-assisted laser desorption/ionization time of flight (MALDI-ToF) mass spectrometry (reflector negative mode using an Ultraflex II instrument, Bruker Daltonics, Billerica, MA)^21^ (Supplementary Fig. 38). A ladder of defined HA oligosaccharides was employed as the mass calibrants (Hyalose, LLC, Oklahoma City, OK).

#### Polymerization reactions and analysis

##### Proof of concept test

Heparosan tetrasaccharide ([GlcA-GlcNAc]_2_; final 30 ng/µL or 41.8 µM) was incubated with 10 mM GlcA and either 10 mM 4-thiol- or authentic UDP-GlcNAc, with PmHS-G (0.5 mg/ml, 4.4 µM), 1 mM MnCl_2_, and 50 mM HEPES buffer at pH 7.2, for 20 h at 30 °C. Here, the thio-donor dimer **11** was first reduced by treatment with 10 mM dithiothreitol in water at 22 °C for 1 h. The reactions were then diluted in water and split into 2 aliquots, an untreated control reaction and a heparin lyase III treated (1 unit/ml in 50 mM ammonium acetate, pH 7.0, for 20 h at 30 °C) reaction. The samples were then analyzed on a 6% 1X tris(hydroxymethyl)aminomethane (Tris)-borate-ethylenediaminetetraacetic acid (TBE) buffer polyacrylamide gel electrophoresis (250 V, 15 min) and stained with 0.05% Alcian Blue followed by silver staining (BioRad Silver Stain kit; Hercules, CA)^21^ (Supplementary Fig. 39). Reaction conditions optimization was pursued as described below.

##### S-link polymerization optimization

In trials to optimize the *S*-link heparosan synthesis and to conserve the UDP-4-SH-GlcNAc precursor, a sequential series of sparse matrix reaction trials were employed to assess which conditions improved the chain polymerization based on radiochemical sugar incorporation assays. Basically, the co-polymerization of the thio-sugar donor with [^3^H]GlcA from UDP-GlcA, the other donor of the heparosan repeating disaccharide structure synthesis, into polysaccharide using a heparosan tetrasaccharide-amine acceptor (GlcA-GlcNAc-GlcA-C_2_H_4_-NH_2_) was monitored. As a control, we performed the identical reactions with the natural UDP-GlcNAc donor in parallel. Typical reaction times were 6 to 48 h. Long polysaccharides (>10–20 monosaccharide units) were separated from the unincorporated, low molecular weight donors by descending paper chromatography^33^. The parameters of pH (pH 4.4–9), metal cation (Mn^++^, Mg^++^, Co^++^, and combinations at 1–10 mM), temperature (22–42 °C), additives (including 29% ethanol, 15–20% methanol, and/or reducing agents, such as 2–20 mM dithiothreitol or 0.1 mM sodium 2-mercaptoethanesulfonate), heparosan synthase catalyst (PmHS1, PmHS2, or their chimeras A, B, D, G, I, or J^21^ were studied. Then promising combinations of the optimal conditions of these groups of factors were re-tested by radioassay. It is noteworthy that the optimal conditions for the natural UDP-GlcNAc precursor did not always match those determined for the *S*-link polysaccharide production.

Finally, independent trials using reactions with higher levels of UDP-sugar donors in various ratios (thio-donor/UDP-GlcA 10:1, 3:1, 2:1, 0.5:1) were tested and analyzed by polyacrylamide gel electrophoresis visualized with Alcian Blue staining. In addition, we employed a fluorescent heparosan-trisaccharide acceptor to increase sensitivity on gels. In some reaction trials, sequential feeding (e.g., add 33% of the total in steps over time) of the UDP-sugars was explored to limit donor hydrolysis and prevent of potential substrate cross-inhibition effects. The molecular weight and band intensity were utilized to compare reaction performance. The final reaction conditions for the hemi-A *S*-linked heparosan polymer are detailed in the relevant method section below.

##### Preparative S-link heparosan synthesis

For larger scale preparations of *S*-linked heparosan, we employed a fluorescent version of the heparosan trisaccharide-NH_2_ glycoside acceptor (GlcA-GlcNAc-GlcA-C_2_H_4_-NH_2_^32^) that was tagged using fluorescein isothiocyanate to serve as a fluorescent acceptor (GlcA-GlcNAc-GlcA-C_2_H_4_-thioamido-fluorescein) to facilitate tracking of the polymer. Approximately one out of every two to three chains was tagged with fluorophore; the untagged versions were spawned by de novo initiation of the chains (no acceptor, *n* = 0 in Fig. 1) that occurs in the prolonged reactions^33^. In these reactions, 4.5 mM UDP-4-SH-GlcNAc (14 mg, 22.5 µmol) and 1.5 mM UDP-GlcA (4.25 mg, 7.5 µmol) were co-polymerized with 12.5 mg PmHS-B in 5 ml of 50 mM HEPES buffer at pH 7.2, 1 mM MnCl_2_, at 30 °C for 2 days with an extra 20% enzyme addition after one day.

After the second day, the polymer was purified by centrifugal clarification (18,000× *g*, 10 min) followed by ultrafiltration in a spin unit with 3 kDa molecular weight cut-off (MWCO) membrane (Amicon Ultra; Millipore), and extraction of the retentate with *n*-butanol, re-clarified by centrifugation, and a second ultrafiltration step, then finally passed through a hydrophobic resin (ZipTipC18; Millipore). This final *S*-linked heparosan preparation was estimated to be ~80 µg based on PAGE analysis with a titration of *O*-linked heparosan standard. Using *O*-linked heparosan molecular weight standards and comparing the log of molecular weight to migration distance, the average size of the unsulfated *S*-linked polymer was determined to be ~18 kDa (range ~13–22 kDa or ~65–110 monosaccharide units; R^2^ 0.99975, Supplementary Fig. 40).

### Polysaccharide sulfation

Two independent methods for sulfation, using either a chemical reagent or recombinant sulfotransferase enzymes (*see* Fig. 3), were employed.

#### Synthesis of heparosan sulfate analogs using a chemical route

##### Aqueous O-sulfation optimization trials

Most recent chemical-based *O*-sulfation reactions with carbohydrates are performed in anhydrous conditions due to the reactivity of typical reagents such as sulfur trioxide or chlorosulfonic acid with water. However, there is precedent for the use of aqueous conditions at high pH^34,35^. As a model polymer, *O*-linked heparosan was tested with a matrix of reaction parameters including pH (e.g., 1 to 4 M NaOH), equivalents of sulfur trioxide-trimethylamine complex (ranging from 2:1 to 55:1 reagent:polysaccharide (*w*:*w*)), temperature (4–30 °C), and reaction time (0.5–48 h). The test reactions were assessed for sulfation levels by agarose gel electrophoresis (2% gels, 1X tris acetate-ethylenediaminetetraacetic acid (EDTA) (TAE) buffer with Stains-All detection^36^. This method reveals sulfation level status by two outputs: band migration (i.e. adding sulfate groups increases charge density, thus increases migration speed) and color (i.e. the heparosan band’s color changes from its initial blue to purple to yellow with increasing levels of sulfation).

It was noted that when using saturated sulfur trioxide-trimethylamine 1 M NaOH at 4 °C for 16–24 h as well as for longer times, the sulfated heparosan moved to a consistent faster position on the gel with a shift from blue to purple; longer times did not cause further migration of a shift to yellow (as observed in reactions treating heparosan with the aggressive agent, chlorosulfonic acid, in anhydrous conditions). Furthermore, the heparosan subjected to these high pH/low-temperature conditions without sulfur trioxide reagent did not break down or stain differently than the starting heparosan polysaccharide. We did note that the parallel reaction mixtures when incubated at 37 °C for 20 min also produced a similar sulfation product to the 4 °C overnight reactions based on band migration and color on agarose/Stains-All gels. However, we wanted to avoid both potential de-acetylation (and subsequent *N*-sulfation) and chain fragmentation that are known to occur in natural *O*-linked GAGs under basic conditions. As the stability of GAGs with thio-glycosidic bonds was hitherto unknown, we pursued the milder conditions using lower temperatures.

##### Preparative chemical sulfation of heparosan polymers

A solution of *O*- or *S*-linked heparosan polymer dissolved in water on ice was adjusted with ice-cold 6 M NaOH to achieve 1 M NaOH and 5 mg/ml carbohydrate final concentrations. This solution was chilled on ice for 10 min then solid sulfur trioxide:trimethylamine complex (Aldrich) was added to 55:1 reagent/polymer *w*/*w* with vortex mixing. The saturated suspension was chilled ~ 5 min on ice, then the reaction was rotated at 4 °C for 16–20 h. The remaining solid sulfation reagent was then removed by centrifugation (10–20,000 × *g*, 5 min at 4 °C), and the solution was neutralized with HCl with mixing. The material was then subjected to 6-7 rounds of ultrafiltration against water using a spin unit (3 kDa MWCO) at 4 °C and then the resulting concentrate was harvested.

#### Synthesis of heparan sulfate analogs using an enzymatic sulfation route

A two-step method (*N*-deacetylation/*N*-sulfation, then 6-*O*-sulfation) was employed to transform heparosan into heparan sulfate.

##### Expression and purification of N-deacetylase/N-sulfotransferase (NSDT)

A secreted form of recombinant NDST (V62-R882) expression was obtained from insect cells (SF9; Cat. # 12-659-017, Gibco) using the baculovirus expression approach^37^. The enzyme was purified from the spent medium using a heparin-Sepharose column (GE Health).

##### Enzymatic sulfation of heparosan polymers

*S*-linked heparosan (~40 μg) or *O*-linked heparosan (~40 μg) were incubated with ~1 mg NDST in a 2.5 ml reaction volume containing 50 mM MES buffer, pH 7.0, 400 mM 3’-phosphoadenosine 5’-phosphosulfate (PAPS) at 37 °C for 4 h. Fresh NDST (1 mg) and 10 μl (100 mM) PAPS were added into the systems at 4, 8, and 12 h. Reactions were stopped after 24 h. Small molecules and salts were removed with 3 kDa MWCO spin unit. The lyophilized crude material was suspended in 400 μl water, proteolyzed at 55^◦^C with 20 μg actinase E for 24 h. The small molecules were removed by 3 K MWCO spin column again. Actinase E was then inactivated by incubation at 95 °C for 30 min. The mixture was lyophilized, then used for the 6-*O*-sulfation modification directly.

The 6-*O*-sulfation of thio*-*linked *N*-sulfo heparosan was performed using 6-*O*-sulfotransferase isoform 3 (6-OST-3)^38^. Briefly, thio-linked *N*-sulfo heparosan (~30 μg) was incubated with 6-OST-3 (1 mg/ml) in 50 mM Tris buffer pH 7.2 and PAPS (12 mM) in a total of 50 μl. The reaction was incubated at 37 °C for two days, then followed by a DEAE Sepharose column purification. The reaction mixtures were mixed with 1 ml of 0.01% Triton X-100 buffer at pH 5.0 containing 150 mM NaCl, 50 mM NaOAc, 3 M urea, 1 mM EDTA, then were loaded on a DEAE Sepharose column (200 µl). The column was washed with 1 ml of the same buffer, and further washed with 1 ml of 0.25 M NaCl, then eluted with 1 ml of 1 M NaCl. The product was dialyzed against water using a 3.5 kDa MWCO membrane (Spectrum) and dried for the subsequent analysis.

### Polysaccharide analyses

#### Quantitation

The amounts of polymers were measured by the carbazole assay with a GlcA standard^32^; every other sugar residue in heparosan-based chains is GlcA.

#### Liquid chromatography/Mass spectrometry-based disaccharide compositional analysis

Polysaccharide samples (~2–50 μg) were desalted and dried before digestion with heparin lyases. An 85 μl aliquot of enzymatic buffer (100 mM sodium acetate/2 mM calcium acetate buffer, pH 7.0) and 15 μl of enzyme cocktail containing 5 mg/ml each of heparin lyase I, II, and III was added to digest samples at 37 °C overnight. The digestion solution was boiled at 100 °C for 10 min and centrifuged to remove the denatured proteins. The disaccharides in the supernatant were recovered and freeze dried. The lyophilized disaccharides were labeled with 2-aminoacridone (AMAC) by adding 10 μl of 0.1 M AMAC in DMSO/acetic acid (17:3 *v*/*v*) incubating at room temperature for 15 min, followed by adding 10 μl of 1 M aqueous sodium cyanoborohydride and incubating for 1 h at 45 °C.

After the AMAC-labeling reaction, the samples were centrifuged, and each supernatant was recovered and analyzed by an optimized version of a previously reported method^39^. High-performance liquid chromatography (HPLC) was performed on an Agilent 1200 LC system at 45 °C using an Agilent Poroshell 120 ECC18 (2.7 μm, 3.0 × 50 mm) column. Mobile phase A and B were 50 mM ammonium acetate aqueous solution or methanol, respectively. The gradient was 0–10 min, 5–45% B; 10-10.2 min, 45–100% B; 10.2-14 min, 100% B; 14–22 min, 100-5% B, all with a flow rate of 300 μl/min. Injection volume was 5 μl. A triple-quadrupole mass spectrometry system equipped with a heated electrospray source (Thermo Fisher Scientific) was used as a detector. The on-line mass spectrometry (MS) analysis used the multiple reaction monitoring (MRM) mode. The ion source parameters were as follows: negative ionization mode with a spray voltage of 3,500 V, sheath gas 60 Arb, Aux gas 30 Arb, and a capillary temperature of 350 °C. The data analysis was performed through Xcalibur software.

MS disaccharide analysis showed that chemical *O*-sulfation of both *O*-linked and the hemi-A thio-linked heparosan resulted in 2-*O*-sulfo-GlcA (13.9-14.7%) and 6-*O*-sulfo-GlcNAc (4.5-8.4%) (Supplementary Figs. 49, 50). Enzymatic sulfation of the *S*-linked heparosan yielded *N*S,6-*O*-sulfo-GlcN (11.3%), *N*S-GlcN (3.8%), and 6-*O*-sulfo-GlcNAc (16.3%) (Supplementary Fig. 51, 52).

#### Polyacrylamide gel electrophoresis

Acrylamide gels (29:1 monomer/bis; BioRad) in 1X TBE were used for polysaccharide analysis; the acrylamide percentage is noted specifically in figures and methods but typically was 6–10% for intact polysaccharides and 10–20% for enzymatic digests including the heparanase kinetic assays. Runs were performed at 250 V for 15–20 min or 30–45 min for the low or high-percentage gels, respectively. Gels of polymers with fluorescent components were imaged (ChemiDoc MP; BioRAD) with the required excitation/emission settings for the dye used, and the total polymer species were then detected with either Alcian Blue (1 h) or Stains-All (15 min) staining as noted followed by de-staining in water.

#### Nuclear magnetic resonance spectrometry analysis of polysaccharides

*O*-linked heparosan (70 μg) or *S*-linked heparosan (70 μg) was dissolved in 80 μl D_2_O and transferred to Shegemi 3 mm NMR microtubes. ^1^H, ^13^C, ^1^H-^1^H COSY and ^1^H-^13^C HSQC NMR spectra were recorded on an 800 MHz (200 MHz for ^13^C NMR) spectrometer (Supplementary Fig. 42–48).

### Enzyme inhibition assays

#### Heparanase enzymatic assays and inhibition studies

A gel-based assay was used to monitor the catalytic action of recombinant human heparanase (R&D Systems; Minneapolis, MN) on polysaccharide substrates. In these assays, fluorescent chains were monitored for cleavage; the disappearance of the parental substrate band is readily noticeable. Each chain contains multiple, overlapping heparanase cut sites so there is a gradual decrease in molecular weight over time. This simple, defined assay directly monitors the digestion of polysaccharides (i.e. glycans > ~20 monosaccharide units) that approximate naturally occurring substrates.

Two types of substrates were employed to follow heparanase activity: (i) a fluorescein end-labeled synthetic *O*-sulfated *O*-linked heparosan or (ii) polyacrylamide gel electrophoretically-purified rhodamine-tagged HS (‘P’ in Supplementary Fig. 56a; see Supplementary Methods section for preparation of both substrates). Heparanase (1.3 ng/μl final) was incubated with substrate (synthetic, 57 ng/μl; HS, 3.3 ng/μl) for various times (typically ~0.2–5 h) in 50 mM sodium acetate, pH 5, 1 mg/ml acetylated bovine serum albumin (Promega; Madison, WI) at 30 °C. Aliquots of reactions were analyzed by polyacrylamide gel electrophoresis (2 μl of reaction/lane; 20% gels, 1X TBE) and fluorescence imaging (5–120 s exposures; ChemiDoc MP). Then the overall size range of the polysaccharide fragments was followed by Stains-All detection.

For the inhibition studies, the two sulfated *S*-linked heparosan preparations were titrated at various molar ratios to the fluorescent substrate. Parallel control digest reactions with 25% or 50% of the enzyme, or gels with 50% of the substrate loaded per lane were used to help calibrate the progress of reactions or level of inhibition. Results from 3 (enzymatic sulfation) or 7 (chemical sulfation) independent titrations of the *S*-linked polymers were used to estimate the IC_50_ (concentration for 50% inhibition of cleavage) for human heparanase; each titration used two time-points that were analyzed on PAGE gels. The ratio of substrate molecules per inhibitor molecule (*Sub/Inhib*) was employed to simply compare the effectiveness of the *S*-linked inhibitors on a molar basis. Control reactions containing half of the enzyme were used as a comparison to assess the IC_50_ of the experimental samples. For comparison to a known heparanase inhibitor, the commercially available compound suramin (Sigma) was titrated (3 titrations, 2 time points/titration) in parallel in the heparanase assays.

#### *Experimental details for* Fig. 4*: O-sulfo-S-linked heparosan analog enzyme challenges and inhibition*

**Panel b**: The chemically *O*-sulfated *S*-link analog or natural *O*-link heparosan (starting materials, **0**; ~0.5 μg/lane) were tested for cleavage by overnight treatment with either the recombinant human heparanase (**H**; 73 ng or 1.3 pmoles/lane) or a mixture of recombinant *Flavobacterium* heparin lyases I, II, and III (**L**; 0.017 units each/lane). *Note*: small sugar fragments do not fix/stain well in this system and run near the dye front.

**Panel c**: PAGE analysis of the kinetics of heparanase enzyme (**Hepase**; load 1 = 0.12 pmoles) cleavage of a fluorescent sulfo-*O*-linked heparosan substrate (load 1 = 3.3 pmoles; marked with arrow) with a titration of the *S*-linked analog inhibitor (either chemically or enzymatically sulfated polymer). The 20-min time point is shown in Fig. 4c; the 45-min samples are shown in Supplementary Fig. 55.

### Polymer molecular simulations

Molecular dynamics simulations of various *O*- or *S*-linked heparosan decasaccharides (non-reducing end [GlcA-GlcNAc]_5_) or *S*-linked heparosan pentasaccharides (non-reducing end [GlcA-GlcNAc]_2_GlcA or GlcNAc-[GlcA-GlcNAc]_2_) with various sulfation patterns (as noted in the figure legends) were performed using the ACEMD software (Acellera Ltd, Stanmore, UK) with GLYCAM force-field topologies and parameters for carbohydrates and glycosaminoglycan extensions^40^. While the standard natural glycosidic linkage (*O*-link) HS residues were part of GLYCAM, the thio-linked (*S*-link) HS analog residues were geometry optimized at HF/6–31 G* QM level using Gaussian09^41^. New partial charges were calculated close to the linkage using RESP^42^ and electrostatic potentials calculated from Gaussian, such that they would correctly amalgamate with the other GLCYAM residues. These calculated atomic partial charges were as follows: S4 -0.46*e*, C1 0.49*e*, O5 -0.56*e*, C2 0.31*e*, C4 0.20*e*, C3 0.32*e*, C5 0.21*e*. All other partial charges were those from GLYCAM to ensure overall integer molecular charges as the sugars were oligomerized in silico.

The glycosidic thio atom was based on the generalized AMBER force-field (GAFF)^43^ “ss” atom type and the bond and angle molecular modeling parameters were based on Gaussian optimization: C-S mean length was 1.8392 Ǻ (force constant 215.9 kcal mol^−1^ Ǻ^−2^) and C-S-C mean angle was 99.24° (force constant 60.2 kcal mol^−1^ rad^−2^). Other missing parameters and dihedrals for “ss” were integrated into GLYCAM from GAFF as required. The “ss” atom type represents a general sp^3^ sulfur with two lone pairs and is designed to be compatible with existing AMBER parameters. Modern force-fields, such as GAFF, are based on quantum mechanical calculations and would be expected to adequately model electronic repulsion such as the exo-anomeric effect. Each decasaccharide was built using the AMBER12 tool tleap^44^ and then added to cubic boxes of TIP3P water^45^, resulting in box side lengths in the range 6.2 − 7.2 nm (~12,000 water molecules). Solute atoms were positioned at least 1.2 nm from the solvent box edge and charge-neutral assemblies were achieved by adding the appropriate number of explicit Na^+^ ions.

Following initial conjugate-gradient energy minimization (1000 steps), each assembly was heated to 298 K and equilibrated in the NPT ensemble for 20 ns prior to 5.3 μs unbiased NVT production molecular dynamics, performed using ACEMD software. The initial 300 ns were discarded, and atomic coordinate data were saved at 10 ps intervals for the remaining 5 µs for subsequent analyses. End-to-end lengths (O1 of the reducing sugar to O4 of the non-reducing terminus), glycosidic angles, and Cremer-Pople ring puckering coordinates^46^ θ and φ (azimuthal and meridional angles, respectively), were computed from complete 5 μs trajectories (i.e., ~500,000 data sets) as described previously^47^. Sinusoidal (equal area) plots were used to depict the pucker conformational data. To more closely approximate the behavior of the polysaccharide (i.e. limit chain-end effects), only the data on the most internal disaccharide is presented in Supplementary Fig. 59, 60. The behavior of the decasaccharides was very similar to that of the pentasaccharides (Supplementary Fig. 62).

### Reporting summary

Further information on research design is available in the Nature Portfolio Reporting Summary linked to this article.

## Supplementary information

Supplementary Information

Reporting Summary

## Data Availability

Supplementary Information is available for this paper. Data is available from the corresponding authors upon request.
