# Supplementary material for: Chemoenzymatic synthesis of sulfur-linked sugar polymers as heparanase inhibitors
Source: Nat Commun. 2022 Dec 2;13:7438. doi: 10.1038/s41467-022-34788-3 (PMC9718760; doi:10.1038/s41467-022-34788-3)
Supplement: Supplementary file 1 — Supplementary Information [file 41467_2022_34788_MOESM1_ESM.pdf]

Supplementary Information for

Chemoenzymatic synthesis of sulfur-linked sugar polymers as heparanase inhibitors

Authors: Peng He,[1†] Xing Zhang,[2†] Ke Xia,[1] Dixy E. Green,[3] Sultan Baytas,[1,4] Yongmei Xu,[5] Truong Pham,[5] Jian Liu,[5] Fuming Zhang,[1] Andrew Almond,[6] Robert J. Linhardt,[1*] Paul L. DeAngelis[3*]

Correspondence to: paul-deangelis@ouhsc.edu & linhar@rpi.edu

Table of Contents

Supplementary Methods

Supplementary Figures:

Fig. 1. Structures of intermediates 2, 3, 5, 7 and 9.

Fig. 2. ^{1}H NMR spectrum of compound **3**.
Fig. 3. ^{13}C NMR spectrum of compound **3**.
Fig. 4. 2D COSY NMR spectrum of compound **3**.
Fig. 5. 2D HSQC NMR spectrum of compound **3**.

Fig. 6. ^{1}H NMR spectrum of compound **4**.
Fig. 7. ^{13}C NMR spectrum of compound **4**.
Fig. 8. 2D COSY NMR spectrum of compound **4**.
Fig. 9. 2D HSQC NMR spectrum of compound **4**.

Fig. 10. ^{1}H NMR spectrum of compound **6**.
Fig. 11. ^{13}C NMR spectrum of compound **6.**
Fig. 12. 2D COSY NMR spectrum of compound **6**.
Fig. 13. 2D HSQC NMR spectrum of compound **6.**

Fig. 14. ^{1}H NMR spectrum of compound **8.**
Fig. 15. ^{13}C NMR spectrum of compound **8.**
Fig. 16. 2D COSY NMR spectrum of compound **8**.
Fig. 17. 2D HSQC NMR spectrum of compound **8**.
Fig. 18. 2D HMBC NMR spectrum of compound **8**.
Fig. 19. ^{31}P NMR spectrum of compound **8**.

Fig. 20. ^{1}H NMR spectrum of compound **10.**
Fig. 21. ^{13}C NMR spectrum of compound **10**.
Fig. 22. 2D COSY NMR spectrum of compound **10**.
Fig. 23. 2D TOCSY NMR spectrum of compound **10**.
Fig. 24. 2D HSQC NMR spectrum of compound **10**.
Fig. 25. ^{31}P NMR spectrum of compound **10**.

Fig. 26. ^{1}H NMR spectrum of compound **11.**
Fig. 27. ^{13}C NMR spectrum of compound **11**.
Fig. 28. 2D COSY NMR spectrum of compound **11**.
Fig. 29. 2D TOCSY NMR spectrum of compound **11**.
Fig. 30. 2D HSQC NMR spectrum of compound **11**.
Fig. 31. ^{31}P NMR spectrum of compound **11**.

Fig. 32. ^{1}H NMR spectrum of compound **12**.
Fig. 33. ^{13}C NMR spectrum of compound **12**.
Fig. 34. 2D TOCSY NMR spectrum of compound **12**
Fig. 35. 2D COSY NMR spectrum of compound **12**.
Fig. 36. 2D HSQC NMR spectrum of compound **12**.
Fig. 37. ^{31}P NMR spectrum of compound **12**.

Fig. 38. MALDI-ToF MS analysis of *O*-linked and hemi-A *S*-linked heparosan in extension reactions.

Fig. 39. PAGE gel of the reaction products made by a heparosan synthase with various UDP-sugar donors and an acceptor, heparosan tetrasaccharide.
Fig. 40. Determination of hemi-A *S*-linked heparosan analog average molecular weight.
Fig. 41. Cleavage of *S*-linked and *O*-linked heparosan with bacterial heparin lyases.

Fig. 42. ^{1}H NMR spectrum of the natural *O*-linked heparosan standard.
Fig. 43. ^{13}C NMR spectrum of *O*-linked heparosan standard.
Fig. 44. ^{1}H NMR spectrum of *S*-linked heparosan polysaccharide.
Fig. 45. ^{13}C NMR spectrum of *S*-linked heparosan polysaccharide.
Fig. 46. 2D COSY NMR spectrum of *S*-linked heparosan polysaccharide
Fig. 47. 2D HSQC NMR spectrum of *S*-linked heparosan polysaccharide.
Fig. 48. NMR analysis of *O*-linked heparosan standard and hemi-A *S*-linked heparosan.

Fig. 49. LC-MS/MS analysis of AMAC-labeled eight heparan sulfate disaccharides of chemically *O*-sulfated heparosan.
Fig. 50. MS disaccharide analysis of the chemically *O*-sulfated heparosans.
Fig. 51. MS disaccharide analysis of NDST-treated *O*- and *S*-linked heparosan.
Fig. 52. MS disaccharide analysis of NDST- and 6*O*-ST-treated *O*- and *S*-linked heparosan.
Fig. 53. Disaccharide composition of enzymatically synthesized *S*-linked HS analog compared to a natural HS extracted from porcine kidney cortex.

Fig. 54. Human heparanase challenge of chemically or enzymatically sulfated *S*-linked polysaccharides.
Fig. 55. Inhibition of human heparanase by sulfated *S*-linked analogs.
Fig. 56. Inhibition of human heparanase digestion of a natural heparan sulfate substrate by sulfated *S*-linked heparan sulfate analogs.
Fig. 57. Inhibition of human heparanase digestion of a fluorescent, chemically sulfated *O*-linked heparosan substrate by the chemically sulfated hemi-A *S*-linked analog and suramin.

Fig. 58. Molecular dynamics predictions of the distribution of end-to-end chain lengths for various heparosan-based decasaccharides.
Fig. 59. Sinusoidal plots of molecular dynamics simulations predictions of the sugar ring pucker conformers of the internal GlcA or GlcNAc residues in various heparosan-based decasaccharide polymers.

Fig. 60. Quantification of azimuthal sugar ring pucker of the internal glucuronic acid residue from multi-µs molecular dynamics simulations of various heparosan-based dodecamers.
Fig. 61. Prevalent hypothetical sugar ring pucker conformers extracted from multi-µs molecular dynamics simulations of various *O*-link and *S*-link heparosan-based decasaccharides.
Fig. 62. Quantification of azimuthal sugar ring pucker of the internal GlcA and GlcNAc residues from multi-µs molecular dynamics simulations of heparosan-based, hemi-A *S*-linked pentamers.

Supplementary Tables:

Tables 1-4. Quantification of the internal glucuronic acid (GlcA) meridional ring pucker.

Supplementary Methods

Synthesis and Analysis of Compounds 1-8 Used for UDP-4-SH-GlcNAc Donor Analog Production

Synthesis of benzyl 2-acetamido-3,6-di-O-benzoyl-2-deoxy-β-D-galacto-pyranoside, **4**

Commercially available D-galactosamine pentaacetate **1** (1.0 g, 2.6 mmol) was refluxed with benzyl alcohol (0.85 ml, 8.2 mmol) and ytterbium(III) trifluoromethanesulfonate (200 mg, 0.3 mmol) in dichloromethane (20 ml) at 60°C for 3 h. The solution was allowed to cool to room temperature and then washed three-times with water. The organic layer was concentrated under reduced pressure and the product was recrystallized from methanol and hexanes to afford **2** as clear crystal in 95% yields.

Sodium methoxide solution (4.82 ml, 0.5 M in methanol) was added to a solution of **2** (350 mg, 0.8 mmol) in anhydrous methanol (10 ml) at room temperature and the resulting solution was stirred for 2 h. The solution was then neutralized with pre-washed and acidified Amberlite IR-120 Hydrogen ion exchange resin and the reaction mixture was filtered through cotton wool to remove the resin. The eluent was then concentrated under reduced pressure to give compound **3** as white solid in 96% yield. The resulting solid was used in the next step without further purification. ^{1}H NMR (800 MHz, methanol-d4) δ 7.30 – 7.25 (m, 4H, H-Ar), 7.23 – 7.20 (m, 1H, H-Ar), 4.84 (d, J = 12.2 Hz, 1H, Ha-OCH$_2$Ph), 4.57 (d, J = 12.1 Hz, 1H, Hb-OCH$_2$Ph), 4.39 (d, J = 8.4 Hz, 1H, H-1), 3.94 (dd, J = 10.7, 8.4 Hz, 1H, H-2), 3.82 – 3.75 (m, 2H, H-4, H-6a), 3.71 (dd, J = 11.4, 5.2 Hz, 1H,, H-6b), 3.54 (dd, J = 10.7, 3.3 Hz, 1H, H-3), 3.45 (ddd, J = 6.6, 5.2, 1.1 Hz, 1H, H-5), 1.90 (s, 3H, methyl H-NHAc). ^{13}C NMR (200 MHz, methanol-d4) δ 172.63 (carbonyl-C-NHAc), 137.89 (C-Ar), 127.90 (C-Ar), 127.44 (C-Ar), 127.22 (C-Ar), 100.77 (C-1), 75.42 (C-5), 71.73 (C-3), 70.04 (C-OCH$_2$Ph), 68.30 (C-4), 61.20 (C-6), 52.89 (C-2), 21.61 (methyl C-NHAc). ESI-HRMS calcd. for $C_{15}H_{22}NO_6$ ([M + Na] $^+$) 312.1442, found 312.1451. (Supplementary Fig. 1-4)

Compound **3** (240 mg, 0.77 mmol) was dissolved in pyridine (4 ml) and the solution cooled to -40°C in a dry ice/ethyl acetate bath. Benzoyl chloride (196 µl, 1.69 mmol) was added dropwise and the solution was allowed to warm to room temperature overnight. The pyridine was removed under vacuum and the resulting crude mixture was purified by column chromatography (hexanes/ethyl acetate 3:1) to yield compound **4** as white solid (307 mg, 78%). ^{1}H NMR (600 MHz, CDCl$_3$) δ 8.14-8.03 (m, 4H, H-Ar), 7.66-7.57 (m, 2H, H-Ar), 7.54-7.43 (m, 4H, H-Ar), 7.39-7.29 (m, 5H, H-Ar), 5.51 (d, J = 8.96, 1H, H-NHAc), 5.35 (dd, J = 2.81, 11.14, 1H, H-3), 4.94 (d, J = 12.14, 1H, H-OCH$_2$OBn), 4.74-4.61 (m, 4H, H-1, H-6, H'-OCH$_2$Ph), 4.51-4.44 (m, 1H, H-2), 4.25 (d, J = 2.47, 1H, H-4), 4.01 (t, J = 6.39, 1H, H-5), 1.87 (s, 3H, methyl H-NHAc); ^{13}C NMR (150 MHz, CDCl$_3$) δ 170.5 (carbonyl-C-NHAc), 166.5 (carbonyl C- Bz), 166.4 (carbonyl C- Bz), 137.1 (C- Ar), 133.7 (C- Ar), 133.5 (C- Ar), 130.3 (C- Ar), 130.1 (C- Ar), 129.9 (C- Ar), 129.8 (C- Ar), 129.3 (C- Ar), 128.7 (C- Ar), 128.6 (C- Ar), 128.3 (C- Ar), 128.2 (C- Ar), 99.8 (C-1), 73.4 (C-3), 72.5 (C-5) , 70.5 (C-OCH$_2$OBn), 67.1 (C-4), 63.0 (C-6), 51.2 (C-2), 23.5 (methyl C-NHAc). ESI-HRMS calcd. for $C_{29}H_{29}NO_8$ ([M + Na] $^+$) 542.1785, found 542.1760. (Supplementary Fig. 5-8)

Synthesis of benzyl 2-acetamido-4-acetylthio-3,6-di-O-benzoyl-2,4-dideoxy-β-D-glucopyranoside, **6**

The dibenzoylated compound **4** (520 mg, 1.0 mmol, 1.0 equiv) was dissolved in pyridine (5 ml) solvent system. The solution was cooled to -10°C, trifluoromethanesulfonic anhydride (0.34 ml, 2.0 mmol, 2.0 equiv) was added dropwise, and the resulting mixture was stirred at 0°C for 3 h.

The mixture was diluted with 20 ml ethyl acetate and washed with water and dried over anhydrous Na_2SO_4. After filtering, the solvent was removed under vacuum to afford the triflate derivative **5** as yellow oil, which was used without further purification.

The triflate derivative **5** from above was dissolved in dimethylformamide (5 ml) and potassium thioacetate (342 mg, 3.0 mmol, 3.0 equiv) was added and stirred at room temperature overnight. The solvent was removed under vacuum and the residue was dissolved in 20 ml ethyl acetate and washed with water. After drying over anhydrous $MgSO_4$, the solvent was removed and the resulting crude mixture was purified by column chromatography (hexanes/ethyl acetate 1:2) to yield **6** as white solid (369 mg, 64%, two steps). ^{1}H NMR (800 MHz, $CDCl_3$) δ: 8.12-8.09 (m, 2H), 7.97-7.94 (m, 2H), 7.60-7.57 (m, 1H, H-Ar), 7.55-7.53 (m, 1H, H-Ar), 7.49-7.46 (m, 2H, H-Ar), 7.42-7.39 (m, 2H, H-Ar), 7.31-7.24 (m, 5H, H-Ar), 5.45 (d, J = 9.14, 1H, H-NHAc), 5.43-5.39 (m, 1H, H-3), 4.87 (d, J = 12.11, 1H, H'-OCH$_2$Ph), 4.69-4.65 (m, 3H, H-6, H-1, H'-OCH$_2$Ph), 4.63-4.60 (m, 1H, H-3), 4.55-4.52 (m, 1H, H-6'), 4.20-4.16 (m, 1H, H-2), 3.99-3.94 (m, 2H, H-4, H-5), 2.16 (s, 3H, methyl H-SAc), 1.81 (s, 3H, methyl H-NHAc); ^{13}C NMR (200 MHz, $CDCl_3$) δ 192.6 (carbonyl C-SAc), 170.1 (carbonyl C-NHAc), 166.6 (carbonyl C- Bz), 166.3 (C- Bz), 137.0 (C-Ar), 133.5 (C-Ar), 133.2 (C-Ar), 130.0 (C-Ar), 129.8 (C-Ar), 129.8 (C-Ar), 128.9 (C-Ar), 128.5 (C-Ar), 128.4 (C-Ar), 128.1 (C-Ar), 128.1 (C-Ar), 128.0 (C-Ar), 99.4 (C-1), 72.9 (C-2), 72.4 (C-5), 70.3 (C-OCH$_2$OBn), 64.1 (C-6), 55.8 (C-3), 44.7 (C-4), 30.7 (methyl C-SAc), 23.3(methyl C-NHAc). ESI-HRMS calcd. for $C_{31}H_{31}NO_8S$ ([M + H] $^+$) 578.1843, found 578.1852 (Supplementary Fig. 9-12).

Synthesis of 1-di-O-benzyl- phospho-2-acetamido-4-acetylthio-3,6-di-O-benzoyl -α-D-glucopyranoside, **8**

$FeCl_3$ (136 mg, 0.84 mmol) was added to a solution of **6** (219 mg, 0.38 mmol) in dry dichloromethane (5 ml) under N_2 protection. After stirring for 3 h, the reaction was quenched by the addition of aq. NH_4Cl. The aqueous layer was extracted with dichloromethane (3 × 15 ml). The combined organic phase was washed with brine, dried over Na_2SO_4 and concentrated under vacuum. The resulting crude product **7** was used in the following step without further purification.

Crude **7** was dissolved in dry tetrahydrofuran (5 ml) and the solution cooled to -78°C. Lithium diisopropylamide solution (2 M in tetrahydrofuran) (0.42 ml, 0.84 mmol) was added dropwise to this cooled solution. After 30 min, tetrabenzyl pyrophosphate (307 mg, 0.57 mmol) was added to the reaction mixture. The reaction was stirred at -78°C for 30 min and slowly warmed to 0°C over 3 h. The reaction was quenched by the addition of aq. NH_4Cl. The aqueous layers were extracted with ethyl acetate (3 × 15 ml), and the combined organic phase was washed with brine, dried over Na_2SO_4 and concentrated under vacuum. The crude was purified by flash chromatography (silica gel, hexanes/ethyl acetate 1:2) to give compound **8** as yellow oil (187 mg, 66%, two steps). ^{1}H NMR (800 MHz, $CDCl_3$) δ: 8.07-8.05 (m, 2H, H-Ar), 7.95-7.92 (m, 2H, H-Ar), 7.54 (t, J = 7.62, 2H, H-Ar), 7.44-7.39 (m, 5H, H-Ar), 7.38-7.31 (m, 9H, H-Ar), 5.79 (dd, J = 3.16, 5.91, 1H, H-1), 5.76 (d, J = 9.07, 1H, H-3), 5.38 (t, J = 10.75, 1H), 5.12-5.08 (m, 1H, H-OCH$_2$Ph), 5.07-5.02 (m, 3H, H-OCH$_2$Ph), 4.56-4.51 (m, 1H, H-2), 4.46-4.39 (m, 2H, H-6, H-6'), 4.22-4.15 (m, 1H, H-5), 4.15-4.10 (m, 1H, H-4), 2.17 (s, 3H, methyl H-SAc), 1.60 (s, 3H, methyl H-NHAc); ^{13}C NMR (200 MHz, $CDCl_3$) δ 191.9 (carbonyl C-SAc), 170.2 (carbonyl C-NHAc), 166.7 (carbonyl C-Bz), 166.2 (carbonyl C-Bz), 135.4 (C-Ar), 135.4 (C-Ar), 135.3 (C-Ar), 133.6 (C-Ar), 133.2 (C-Ar), 130.0 (C-Ar), 129.8 (C-Ar), 129.6 (C-Ar), 128.9 (C-Ar), 128.8 (C-Ar), 128.7 (C-Ar), 128.5 (C-Ar), 128.4 (C-Ar), 128.1 (C-Ar), 128.1 (C-Ar), 96.8 (d, J = 5.95, C-1), 70.8 (C-5), 70.1 (d, J = 5.20, C-OCH$_2$Ph) , 69.9 (d, J = 5.20, C-OCH$_2$Ph), 69.8 (C-OCH$_2$Ph), 63.2 (C-6), 53.2 (d, J = 7.51,

C-2), 43.6 (C-4), 30.6 (methyl C-SAc), 22.7 (methyl C-NHAc). ^{31}P NMR (243 MHz, D_2O) δ -2.35. ESI-HRMS calcd. for $C_{38}H_{38}NO_{11}SP$ ($[M - H]^-$) 746.1830, found 746.1815. (Supplementary Fig. 13-18).

Preparation of substrates for heparanase kinetic assays

Two different fluorescent substrates were employed in the heparanase kinetic assay (see main Methods section): a synthetic sulfated heparosan and a natural heparan sulfate preparation.

Fluorescein end-labeled sulfo *O*-link heparosan

A defined synthetic fluorescent *O*-linked heparosan that was structurally similar to the *S*-linked heparosan except for containing only natural *O*-glycosidic linkages was used to monitor heparanase digestion; this quasi-monodisperse polysaccharide was labeled with a single fluorescein on the reducing terminus resulting in a tight band on polyacrylamide gels making any chain cleavage facile to detect. A 40-kDa *O*-linked heparosan with an amino group at its reducing end (installed using the heparosan-trisaccharide-ethyl-amino acceptor) was prepared through chemoenzymatic synthesis[33] and then tagged using fluorescein isothiocyanate (50-fold excess) in 30% dimethyl sulfoxide (DMSO), 100 mM HEPES buffer at pH 7.2, overnight at room temperature with mixing. The reaction was then diluted 9-fold with water, adjusted to 0.4 M NaCl, and 3 volumes of ethanol was added to initiate precipitation. After 3 h at -20°C, the target polysaccharide was recovered by centrifugation. The resulting pellet was washed with 70% ethanol, 0.1 M NaCl and air-dried. After re-suspension in water, the residual free dye was removed by extensive ultrafiltration against water in a spin unit as above. The polysaccharide was then chemically sulfated as with the *S*-link polymer preparation as described above, then purified by ultrafiltration against water.

Rhodamine-labeled heparan sulfate

A well-characterized natural preparation of porcine intestinal heparan sulfate (HS, 12.9 kDa) derived from a pharmaceutical heparin production run (Celsus Pharmaceuticals, Franklin, OH) was tagged with rhodamine isothiocyanate on internal $GlcNH_2$ residues (~1 unsubstituted amino group/chain) with 50-molar excess reagent as in the process for preparing fluorescein-labeled heparosan. The polydisperse material runs as a broad smear, therefore, to facilitate gel-based analysis in heparanase assays, it was subjected to preparative polyacrylamide gel electrophoresis (7% gel, 1X TBE buffer) purification. Briefly, a section of an abundant material in the higher MW area of the smear was excised with a razor, pulverized with a micro-pestle, and the fragments extracted thrice with water (~2:1 water/gel *v*/*v*; end-over-end rotation at 4°C for ~5-16 h per extraction cycle). The gel pieces were removed by centrifugation (20,000 × *g*, 5 min). The eluates were concentrated and de-salted against water with an ultrafiltration spin unit (Supplementary Fig. 55A; labeled as ‘**P**’ on gel).

Supplementary Figures

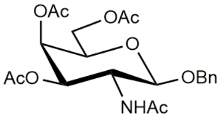

2

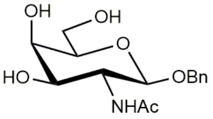

3

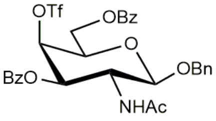

5

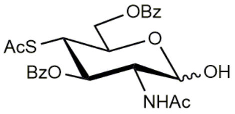

7

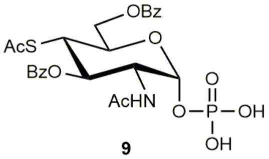

9

Supplementary Fig. 1. Structures of intermediates **2, 3**, **5**, **7** and **9**.

In the Main Fig. 2, the scheme of UDP-4SH-GlcNAc synthesis, three unstable intermediates (**5**, **7** and **9**) were used in impure form and processed directly without analysis

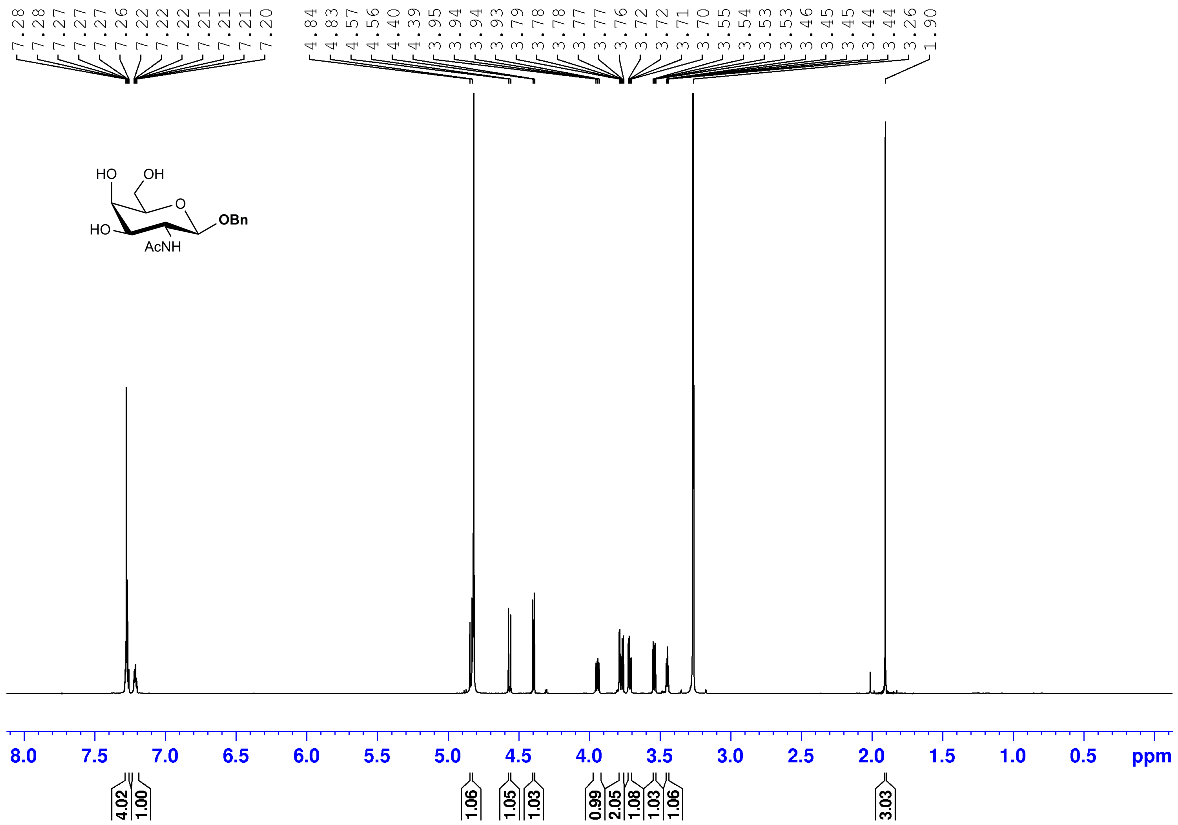

Supplementary Fig. 2. ^{1}H NMR spectrum (800 MHz, Methanol-d6) of compound **3**.

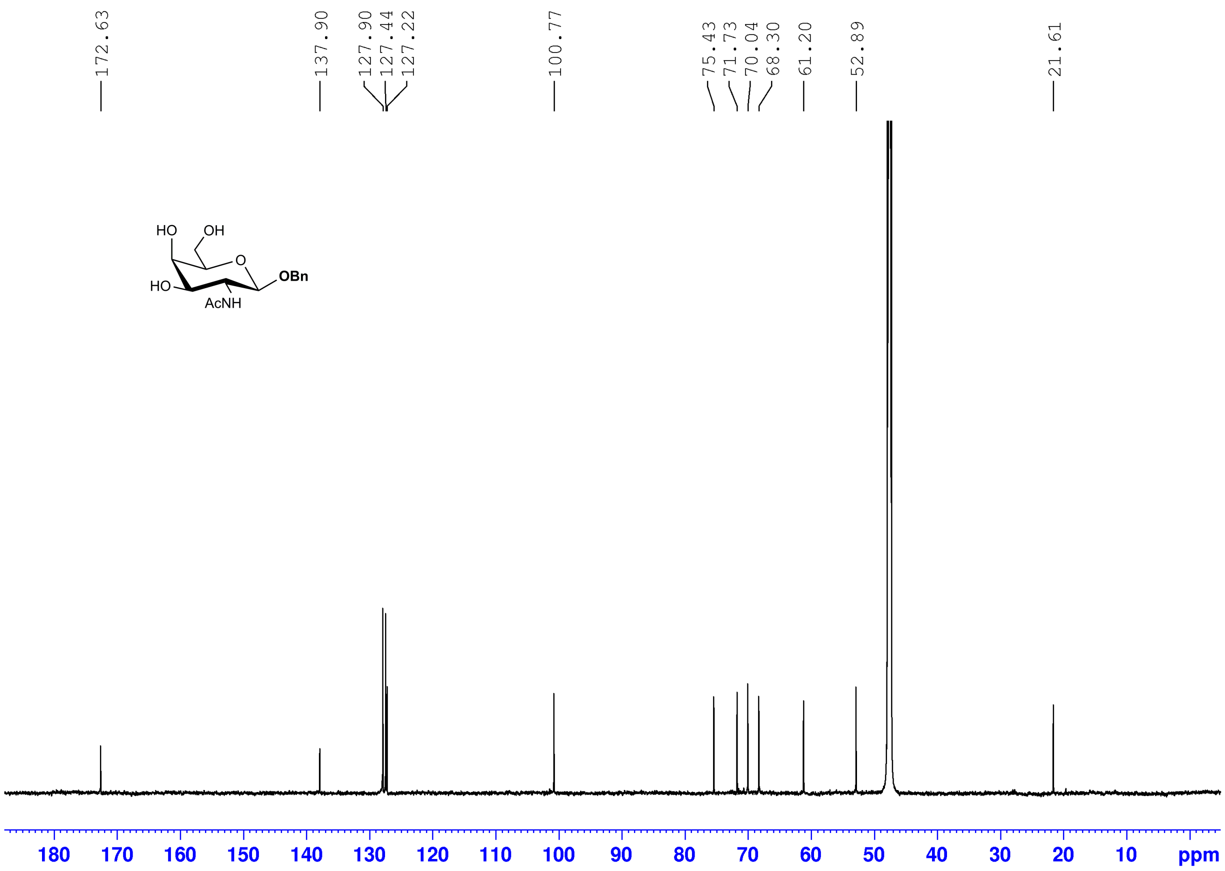

Supplementary Fig. 3. ^{13}C NMR spectrum (800 MHz, Methanol-d6) of compound **3**.

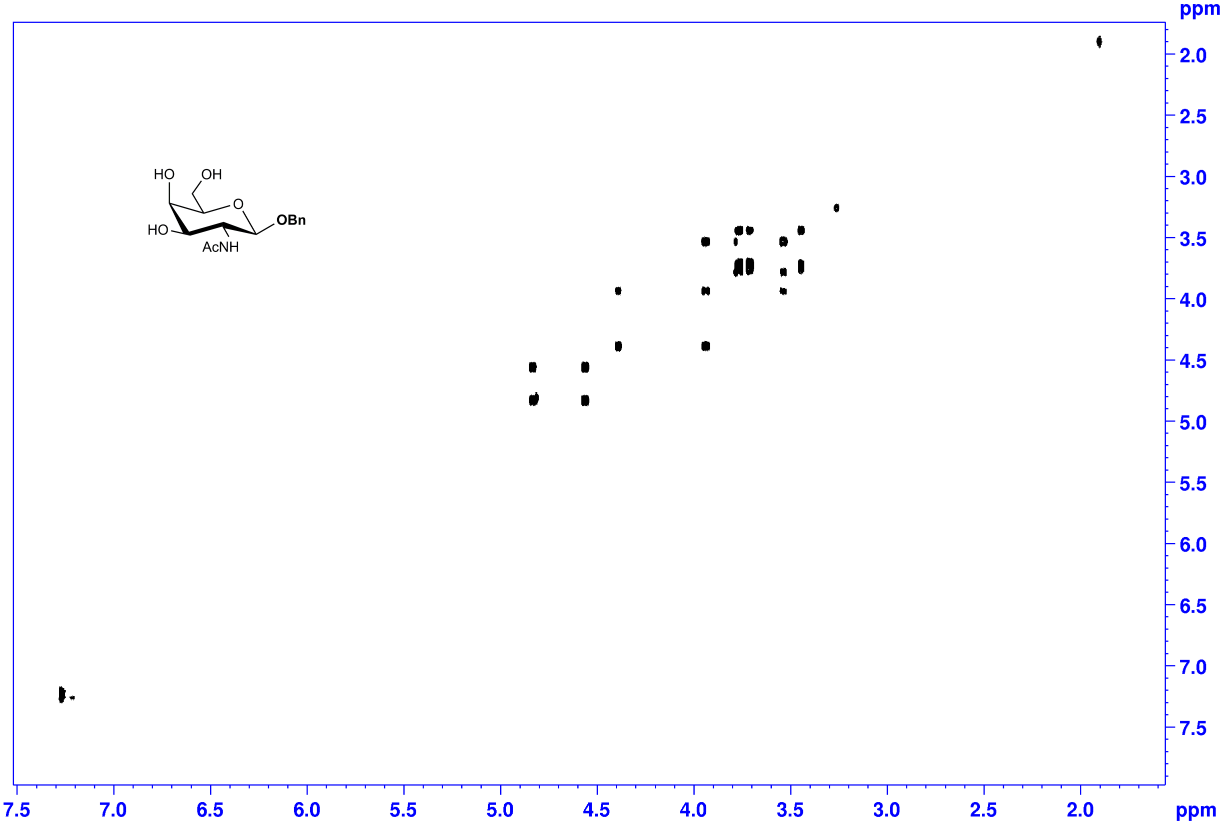

Supplementary Fig. 4. 2D COSY NMR spectrum (800 MHz, Methanol-d6) of compound **3**.

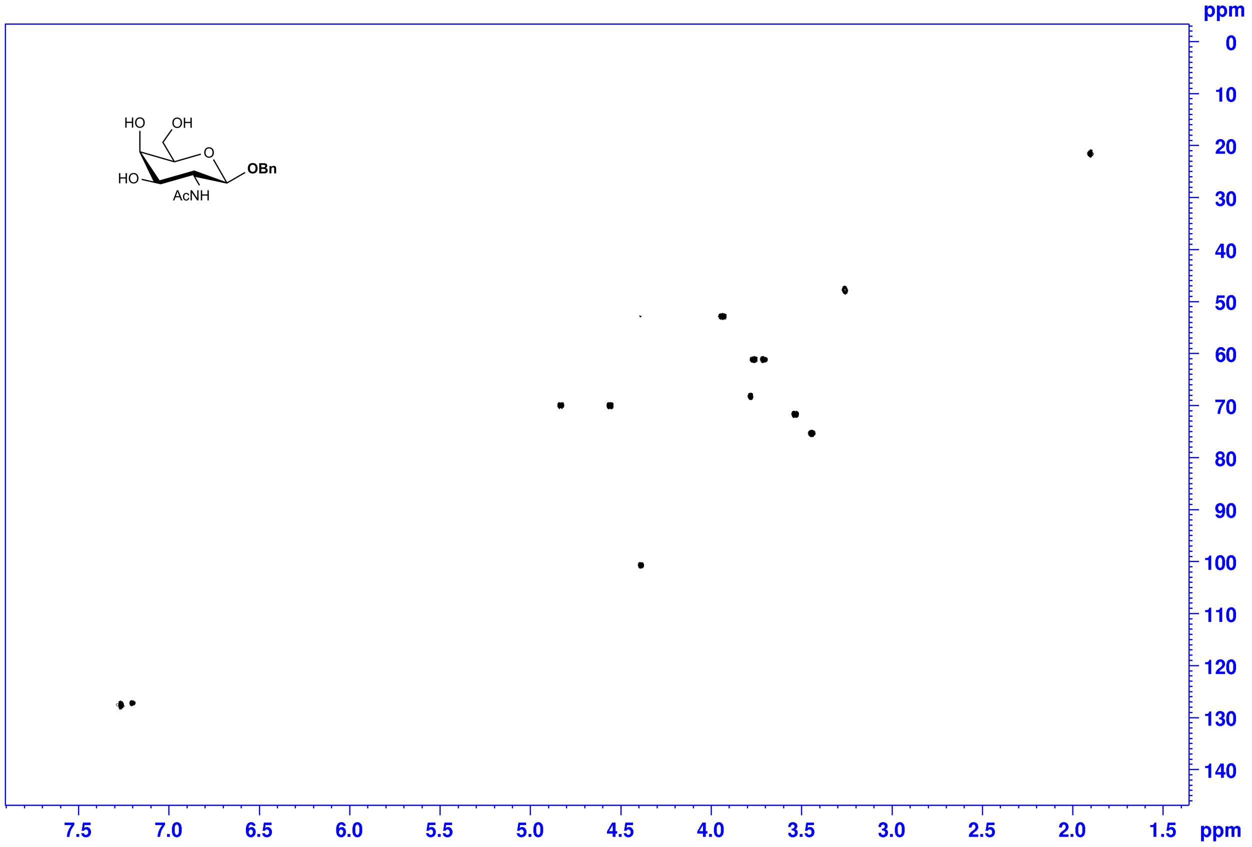

Supplementary Fig. 5. 2D HSQC NMR spectrum (Methanol-d6) of compound **3**.

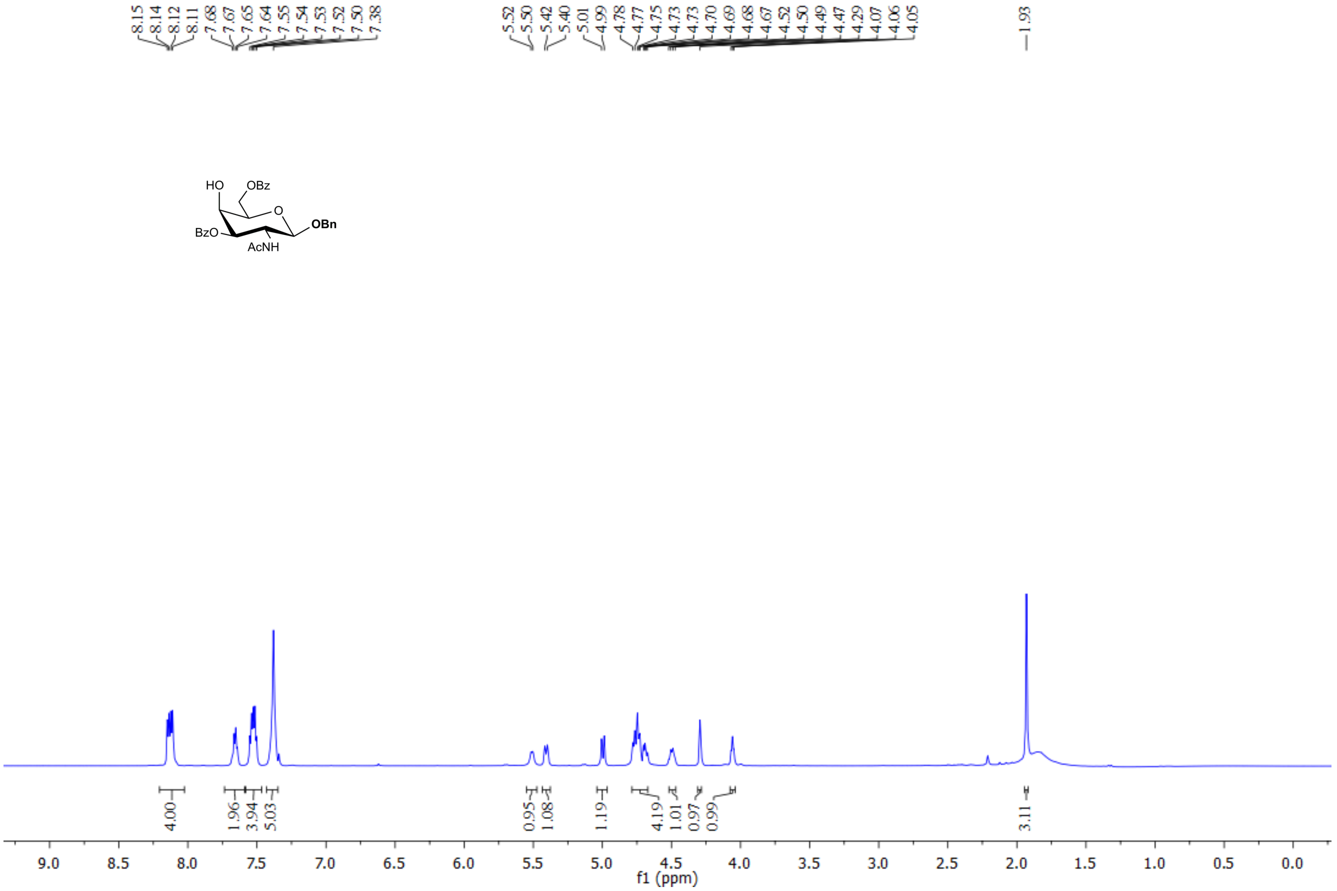

Supplementary Fig. 6. ^{1}H NMR spectrum (600 MHz, $CDCl_3$) of compound **4**.

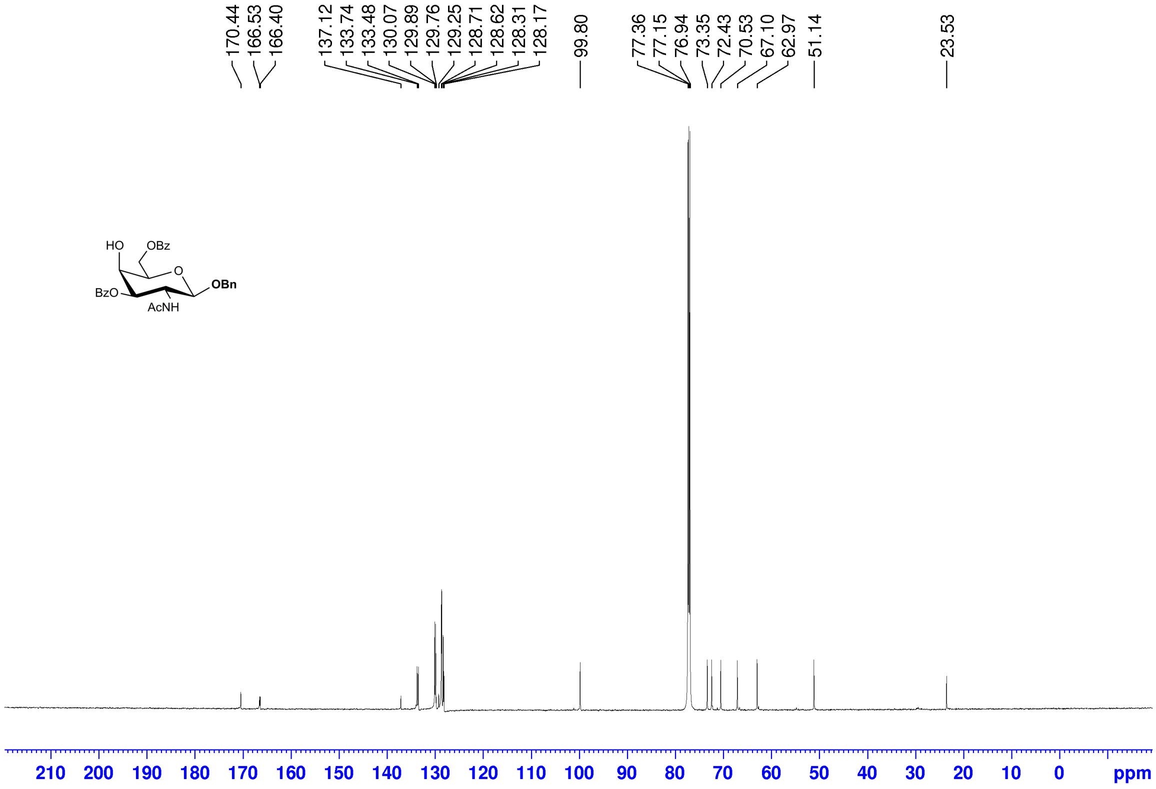

Supplementary Fig. 7. ^{13}C NMR spectrum (600 MHz, $CDCl_3$) of compound **4**.

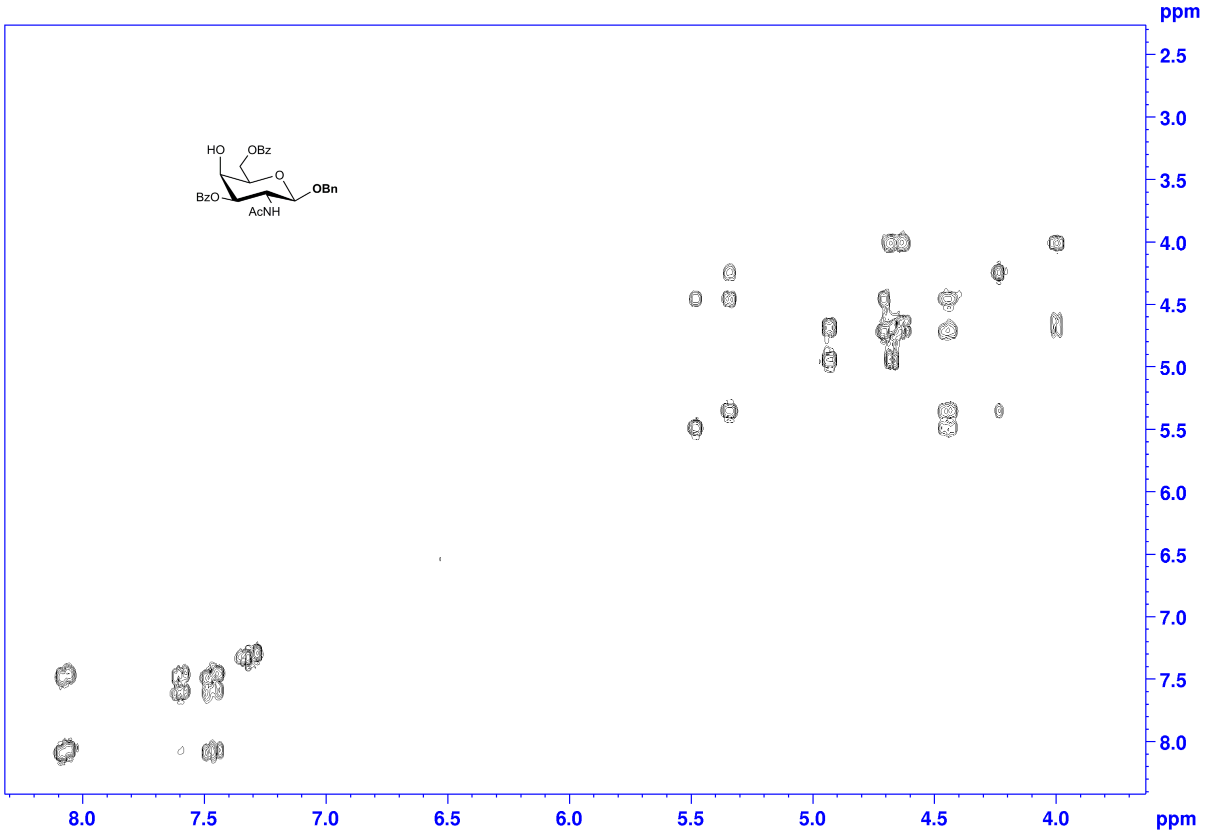

Supplementary Fig. 8. 2D COSY NMR spectrum (600 MHz, $CDCl_3$) of compound **4**.

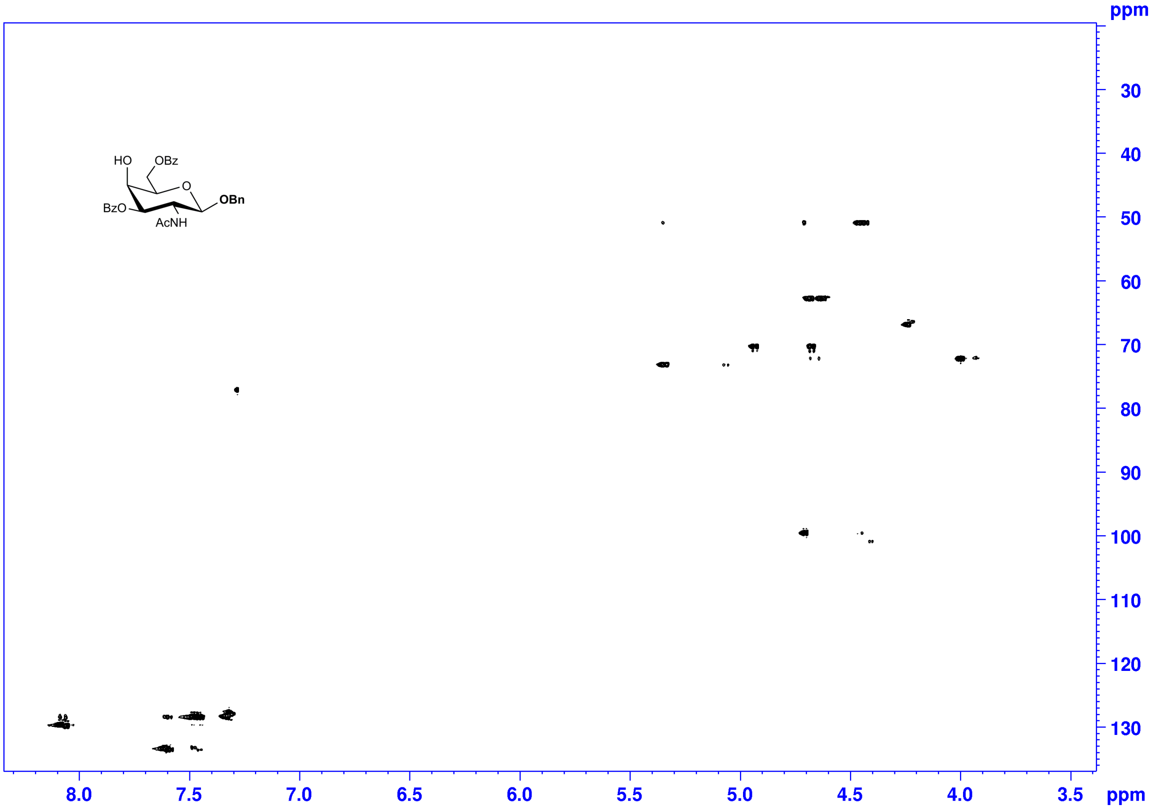

Supplementary Fig. 9. 2D HSQC NMR spectrum (600 MHz, $CDCl_3$) of compound **4**.

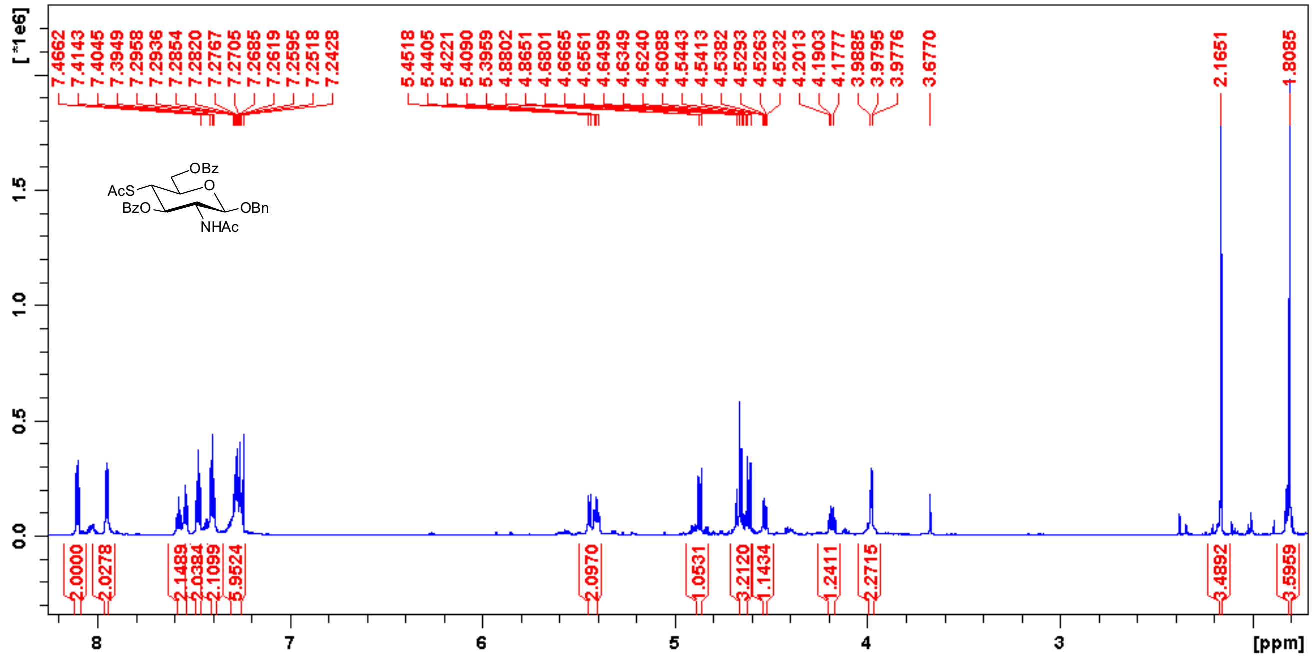

Supplementary Fig. 10. ^1H NMR spectrum (600 MHz, $CDCl_3$) of compound **6**.

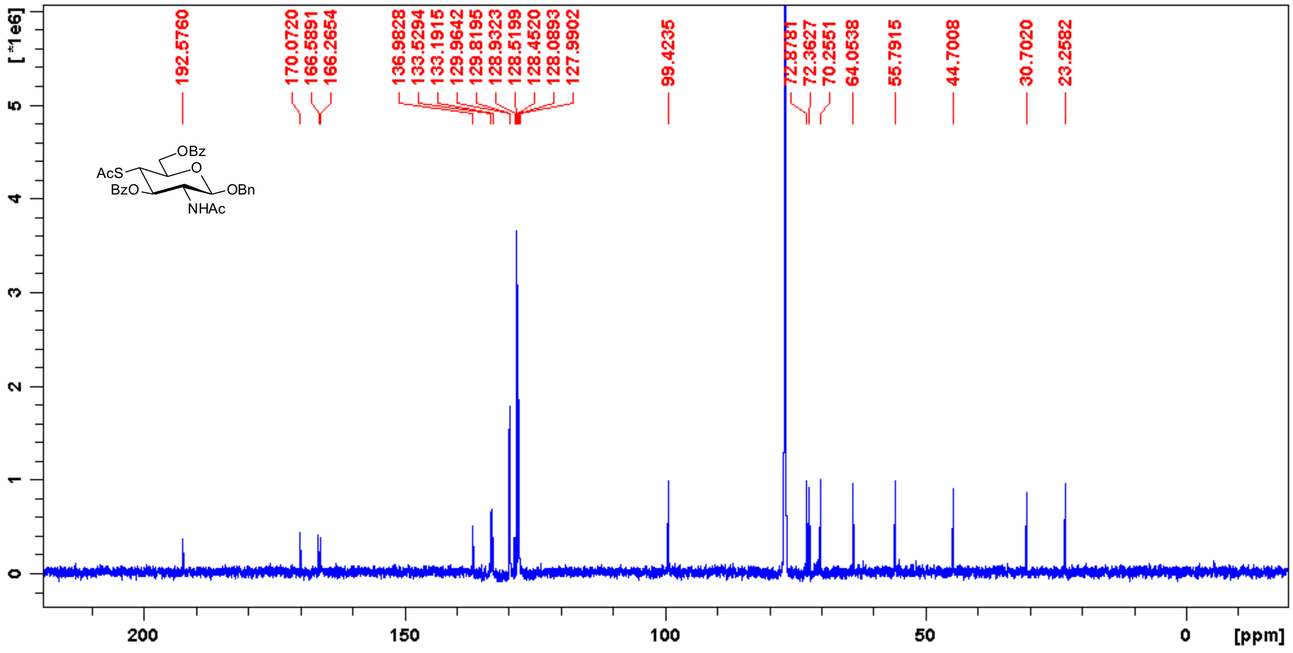

Supplementary Fig. 11. ^{13}C NMR spectrum (150 MHz, $CDCl_3$) of compound **6**.

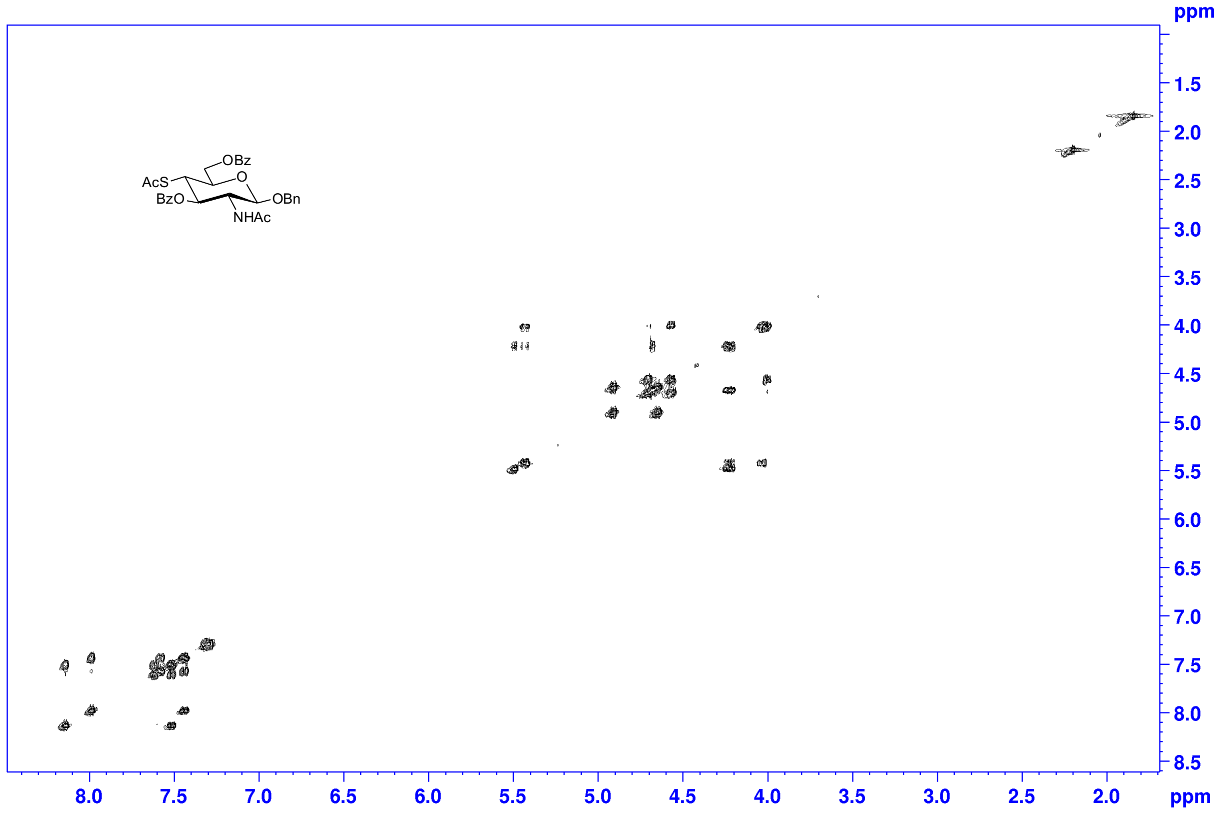

Supplementary Fig. 12. 2D COSY NMR spectrum (600 MHz, $CDCl_3$) of compound **6**.

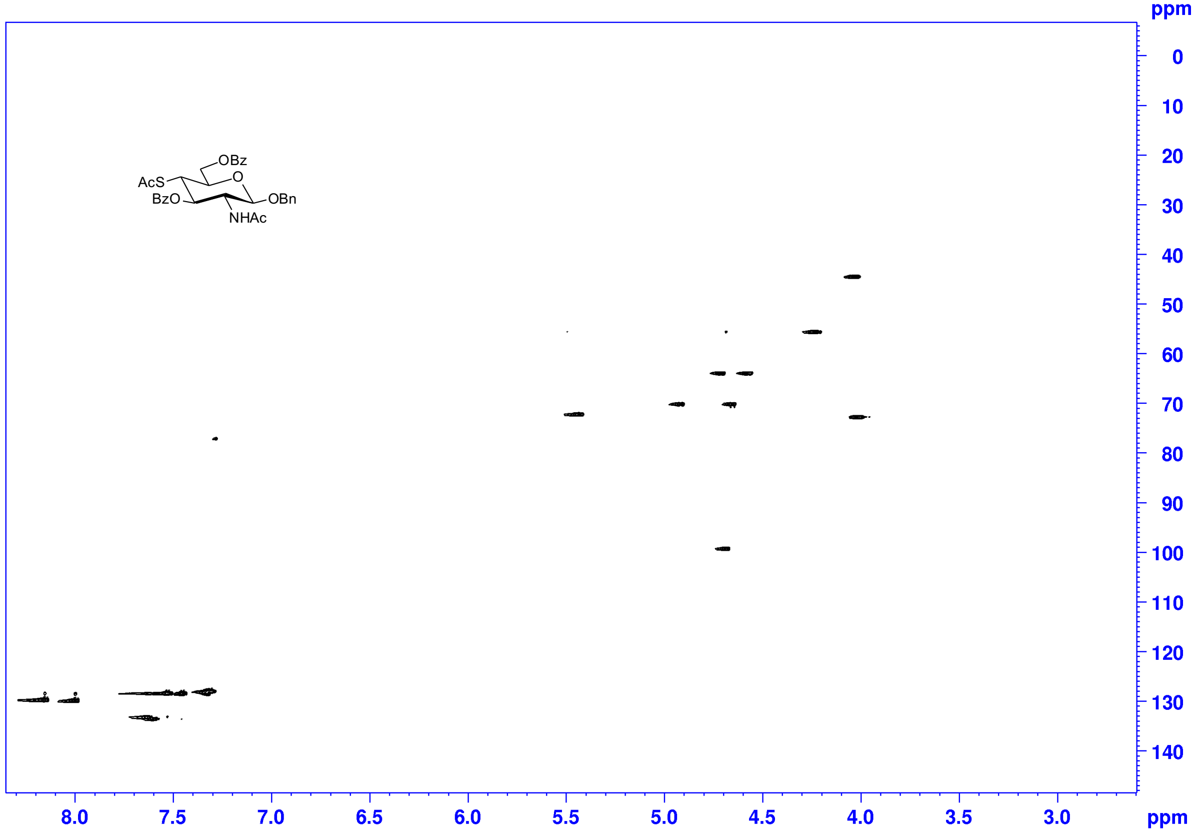

Supplementary Fig. 13. 2D HSQC NMR spectrum (600 MHz, $CDCl_3$) of compound **6.**

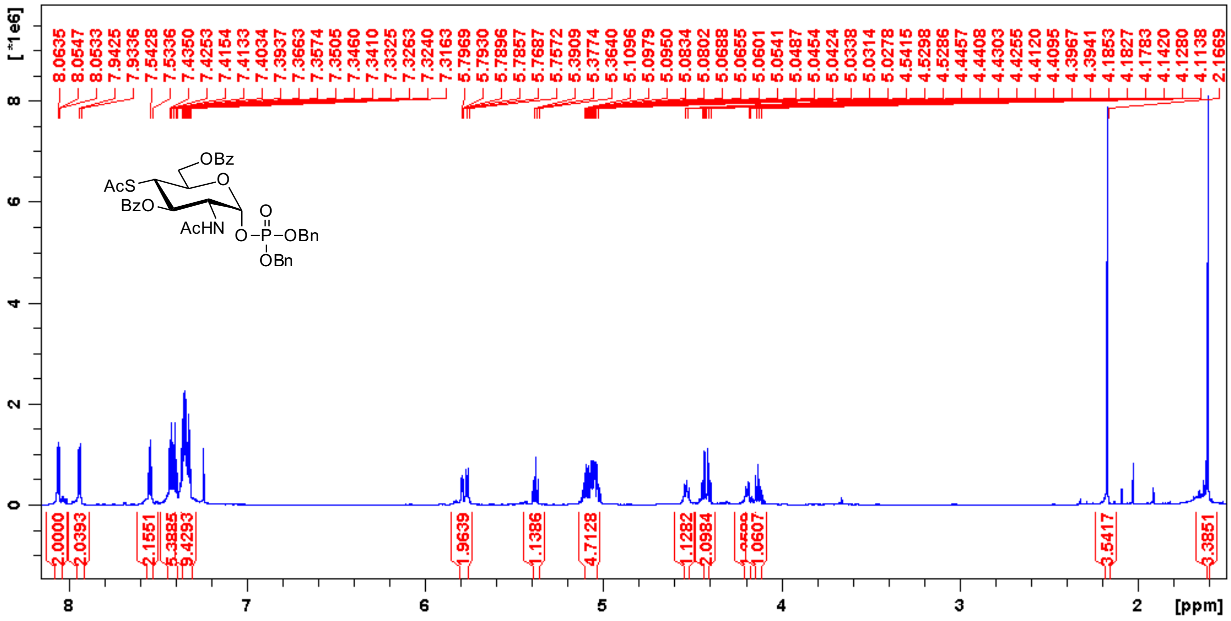

Supplementary Fig. 14. ^{1}H NMR spectrum (800 MHz, $CDCl_3$) of compound **8.**

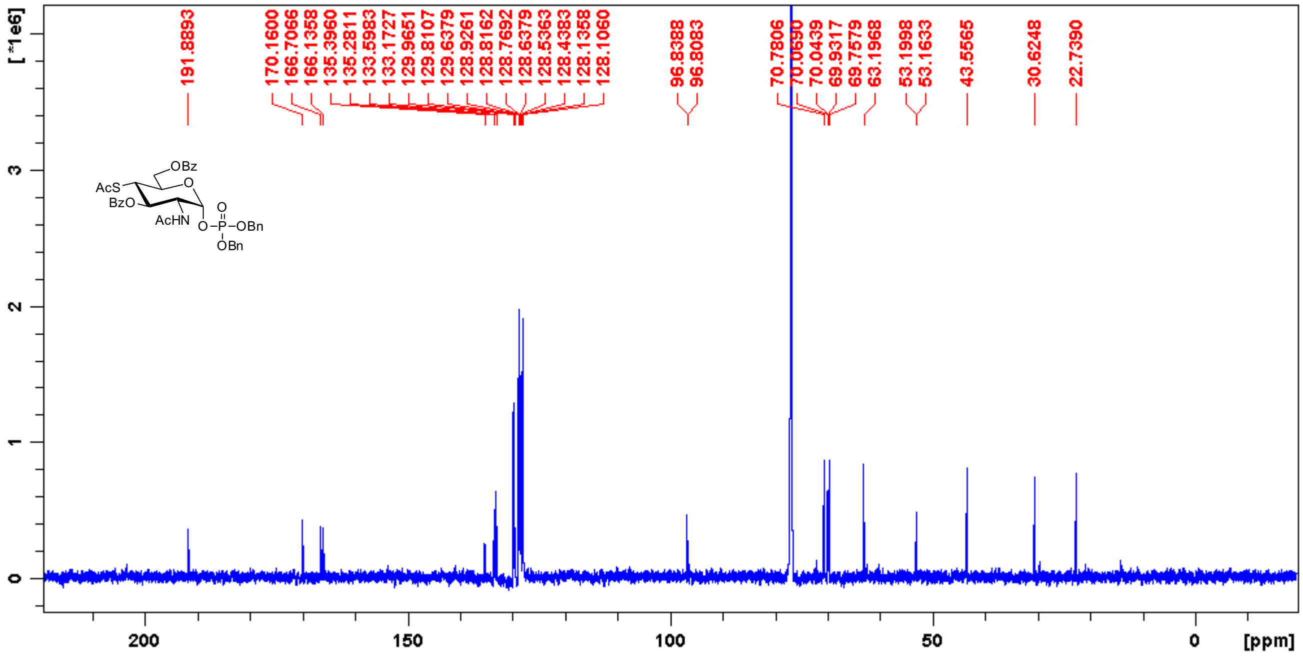

Supplementary Fig. 15. ^{13}C NMR spectrum (200 MHz, $CDCl_3$) of compound **8**.

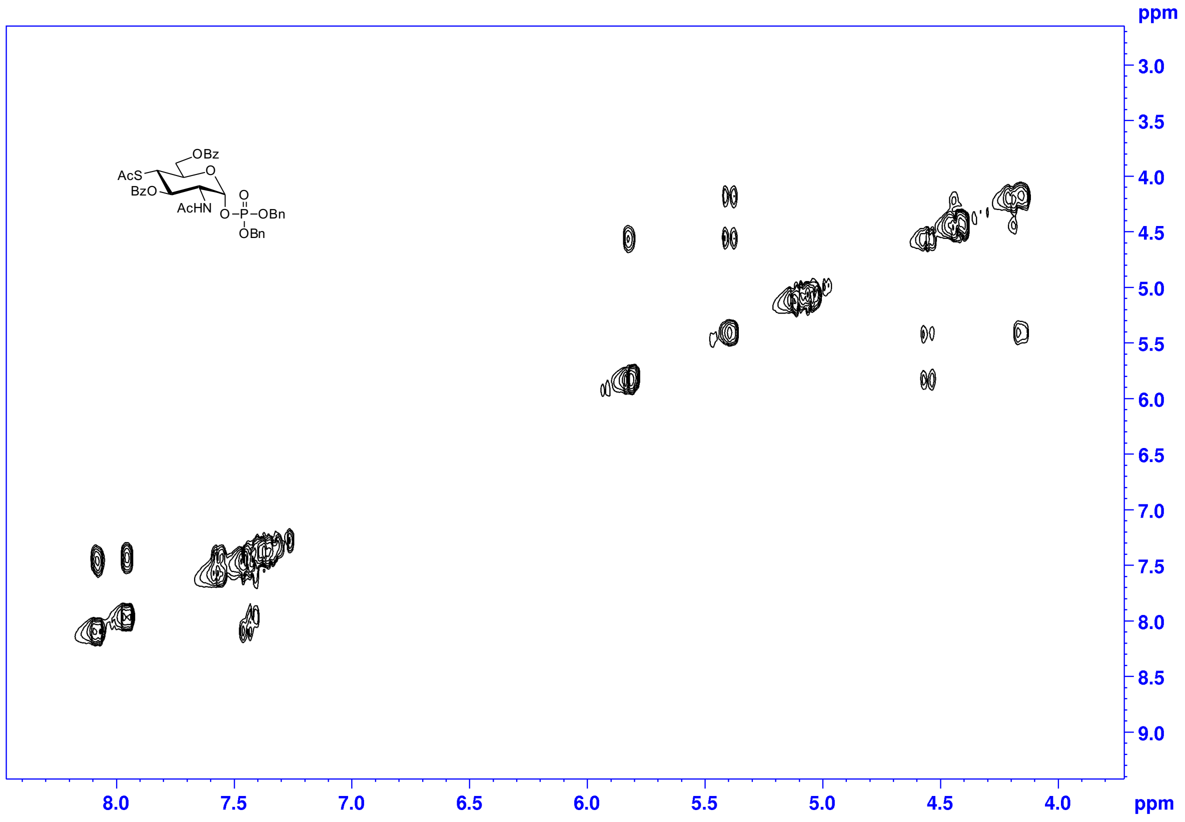

Supplementary Fig. 16. 2D COSY NMR spectrum (800 MHz, $CDCl_3$) of compound **8**.

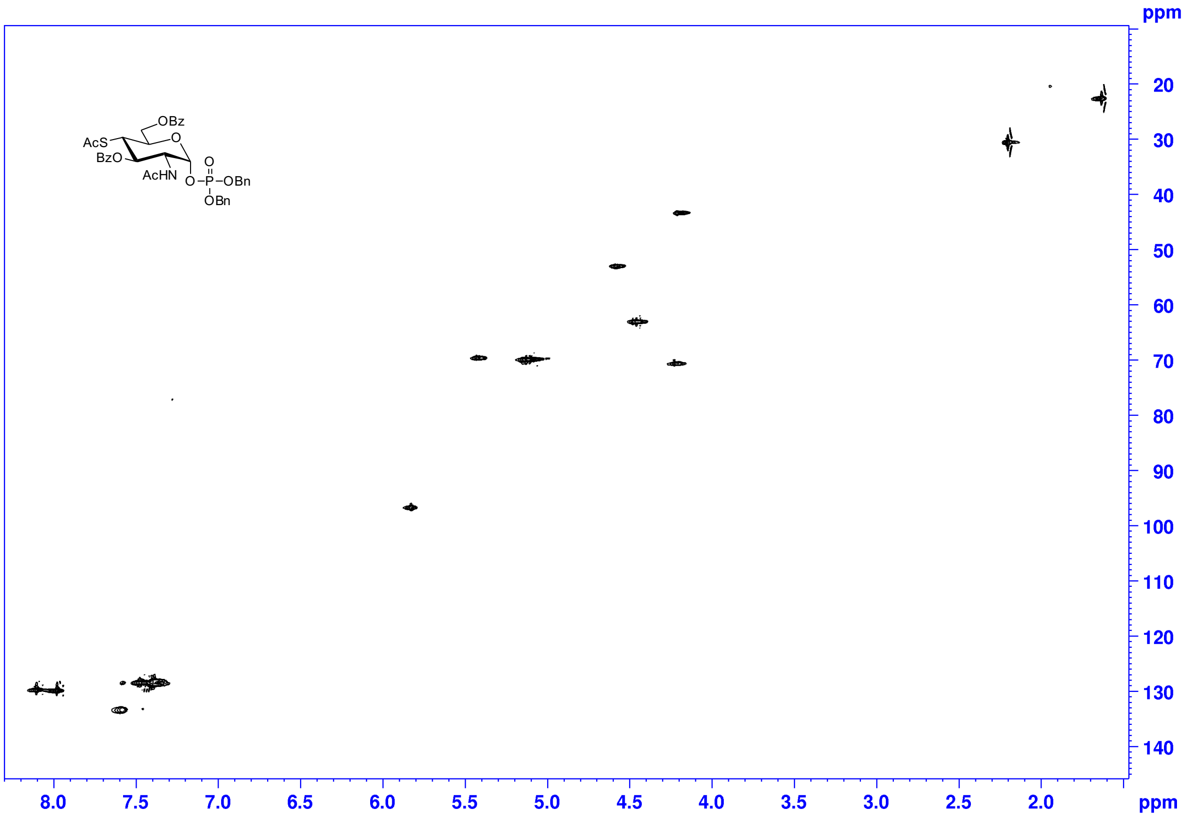

Supplementary Fig. 17. 2D HSQC NMR spectrum (800 MHz, $CDCl_3$) of compound **8**.

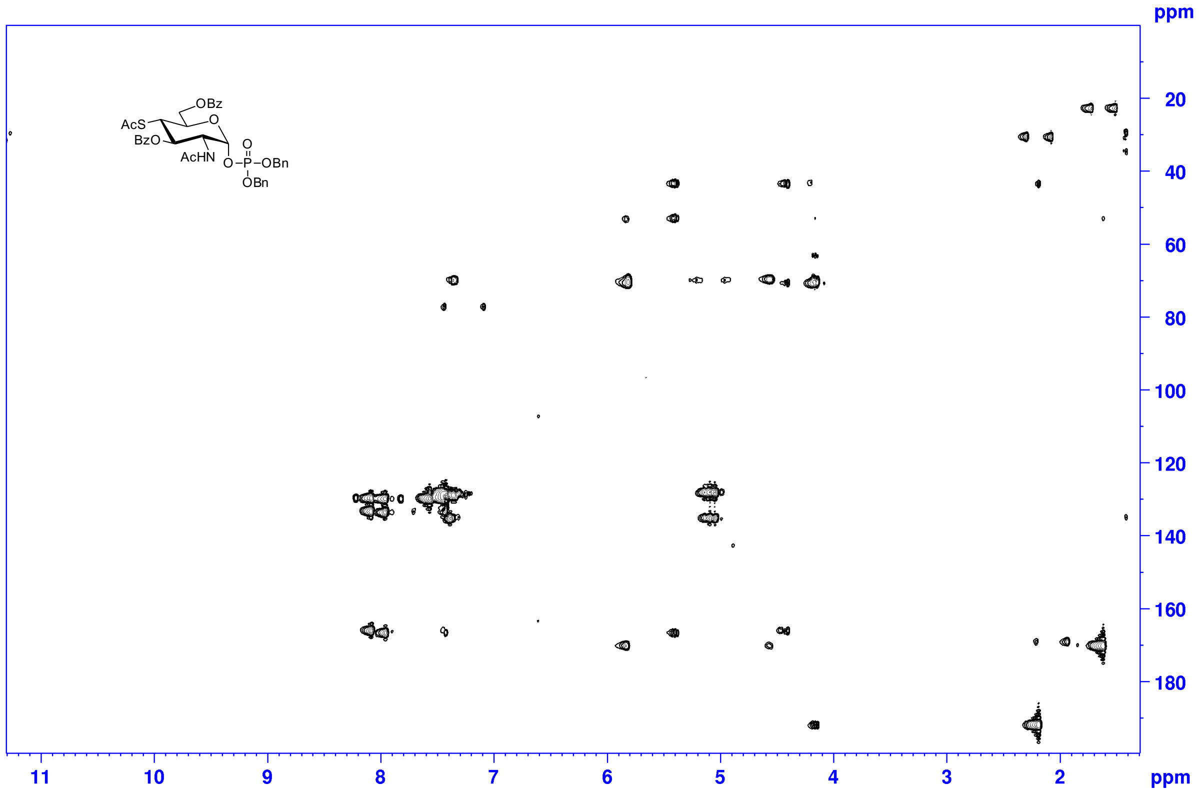

Supplementary Fig. 18. 2D HMBC NMR spectrum (800 MHz, $CDCl_3$) of compound **8**.

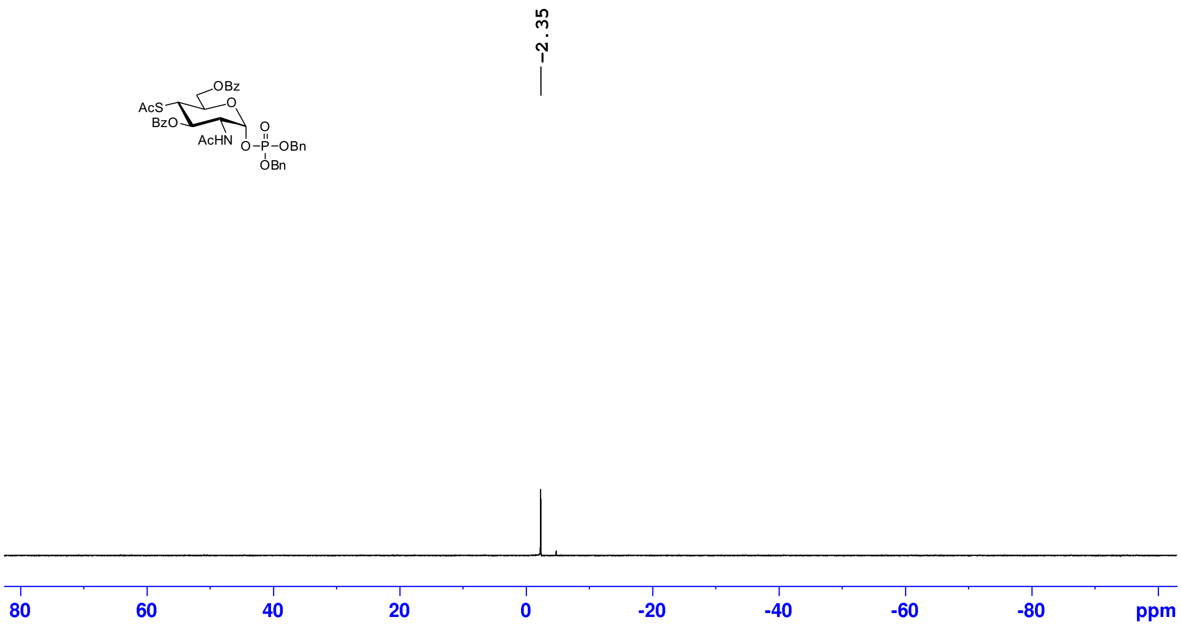

Supplementary Fig. 19. ^{31}P NMR spectrum (243 MHz, $CDCl_3$) of compound **8**.

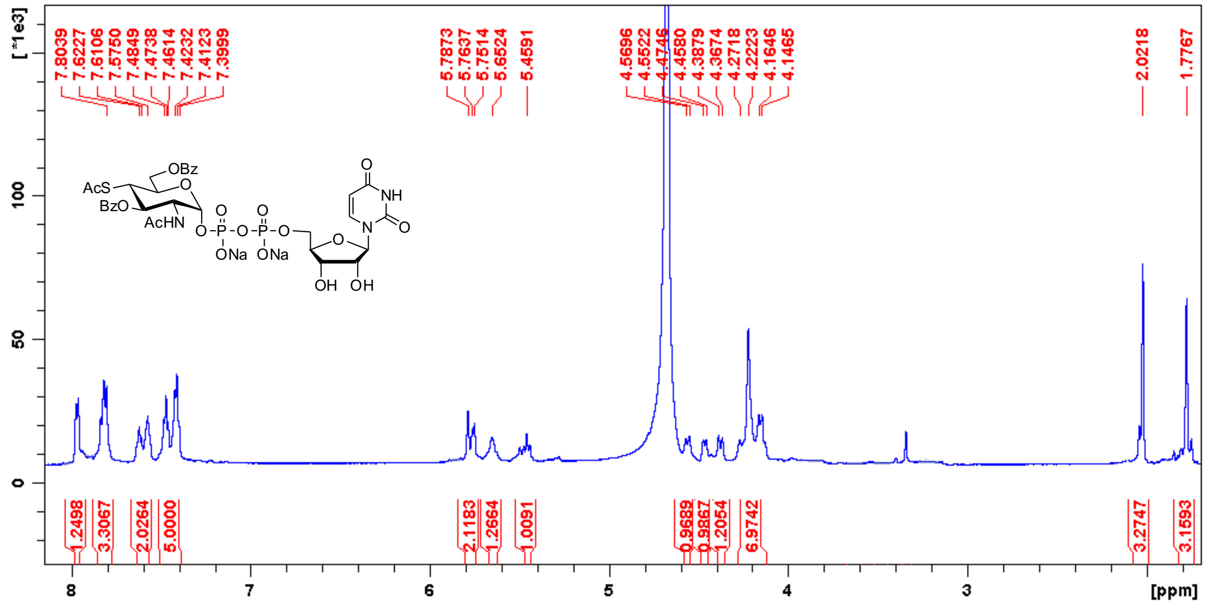

Supplementary Fig. 20. ^{1}H NMR spectrum (600 MHz, D_2O) of compound **10**.

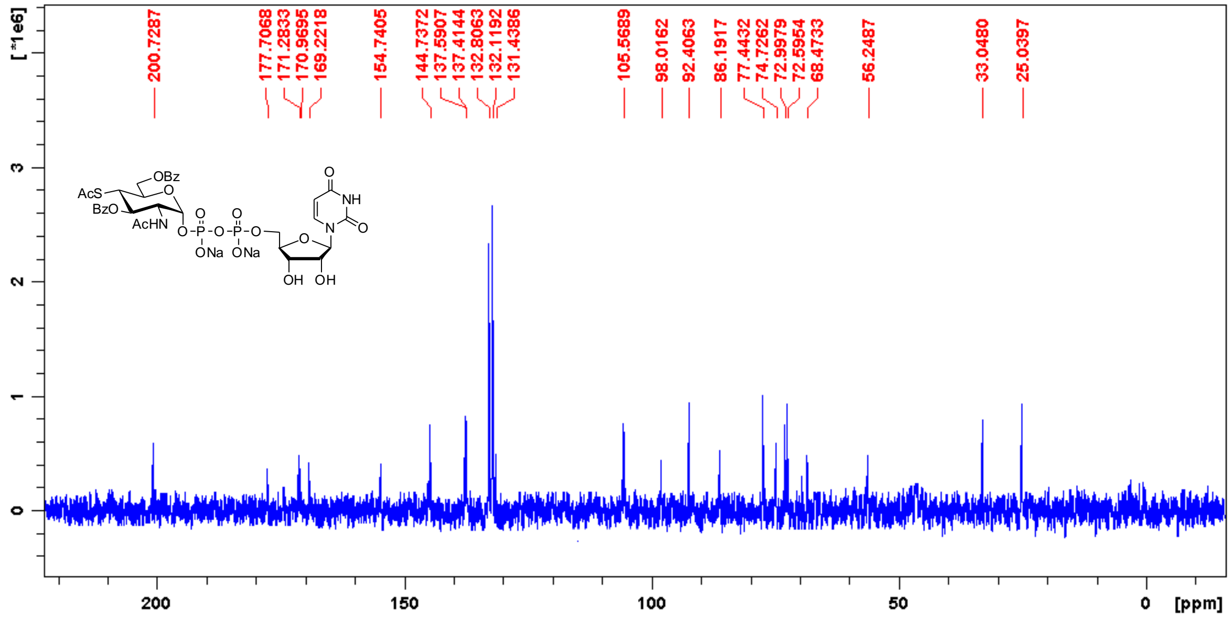

Supplementary Fig. 21. ^{13}C NMR spectrum (150 MHz, D_2O) of compound **10**.

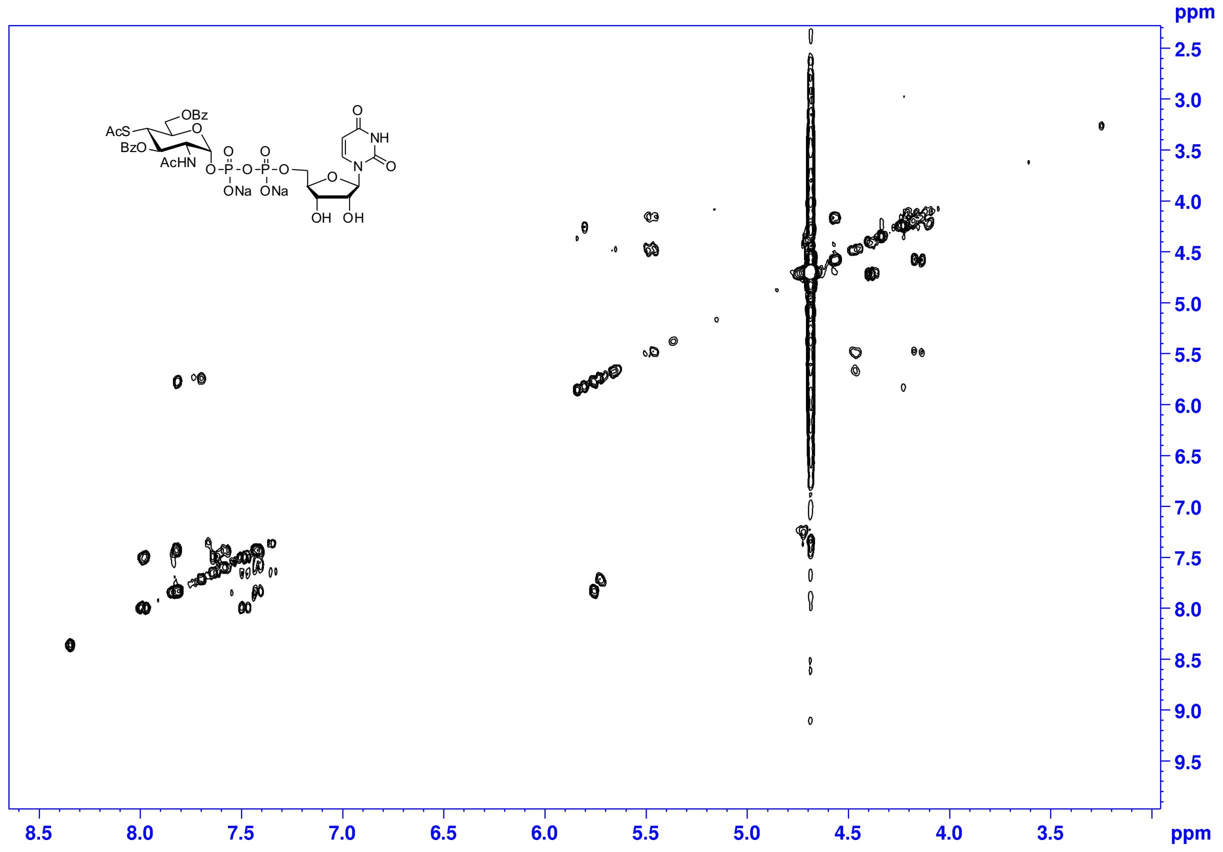

Supplementary Fig. 22. 2D COSY NMR spectrum (600 MHz, D_2O) of compound **10**.

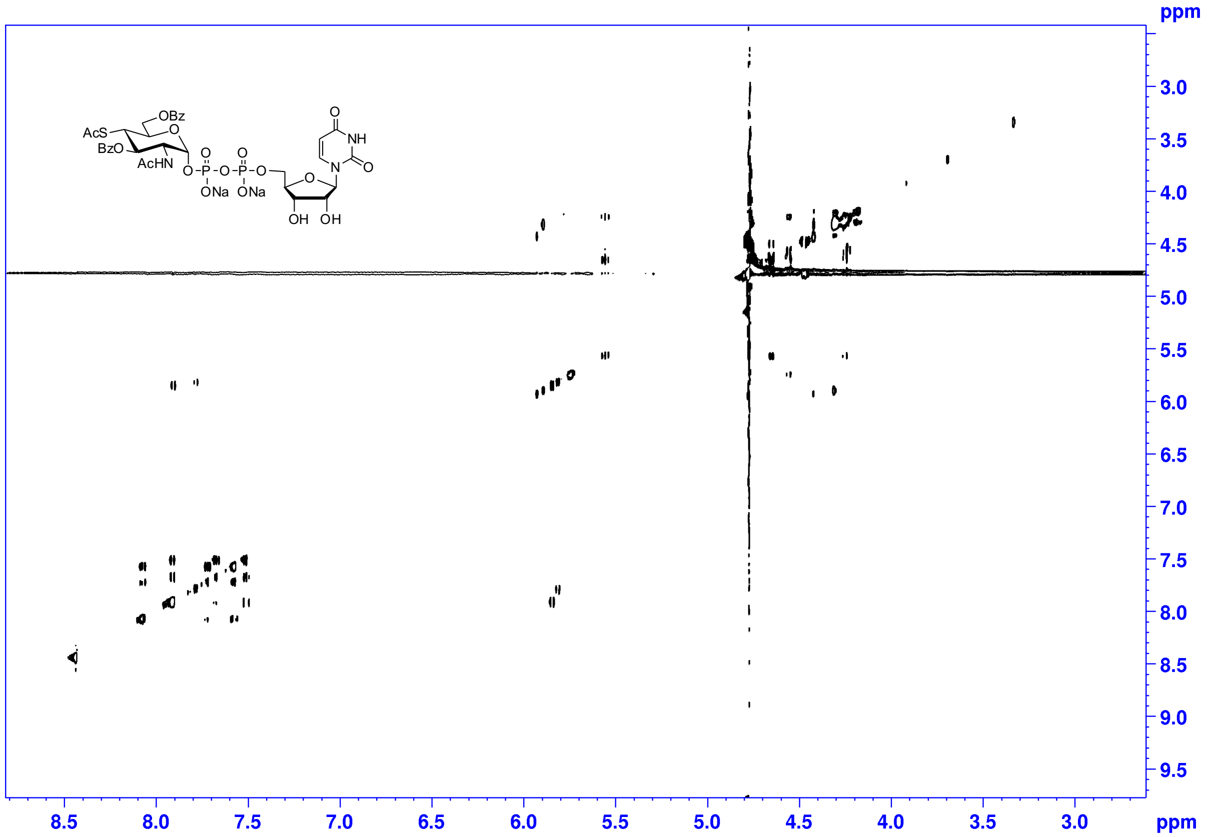

Supplementary Fig. 23. 2D TOCSY NMR spectrum (600 MHz, D_2O) of compound **10**.

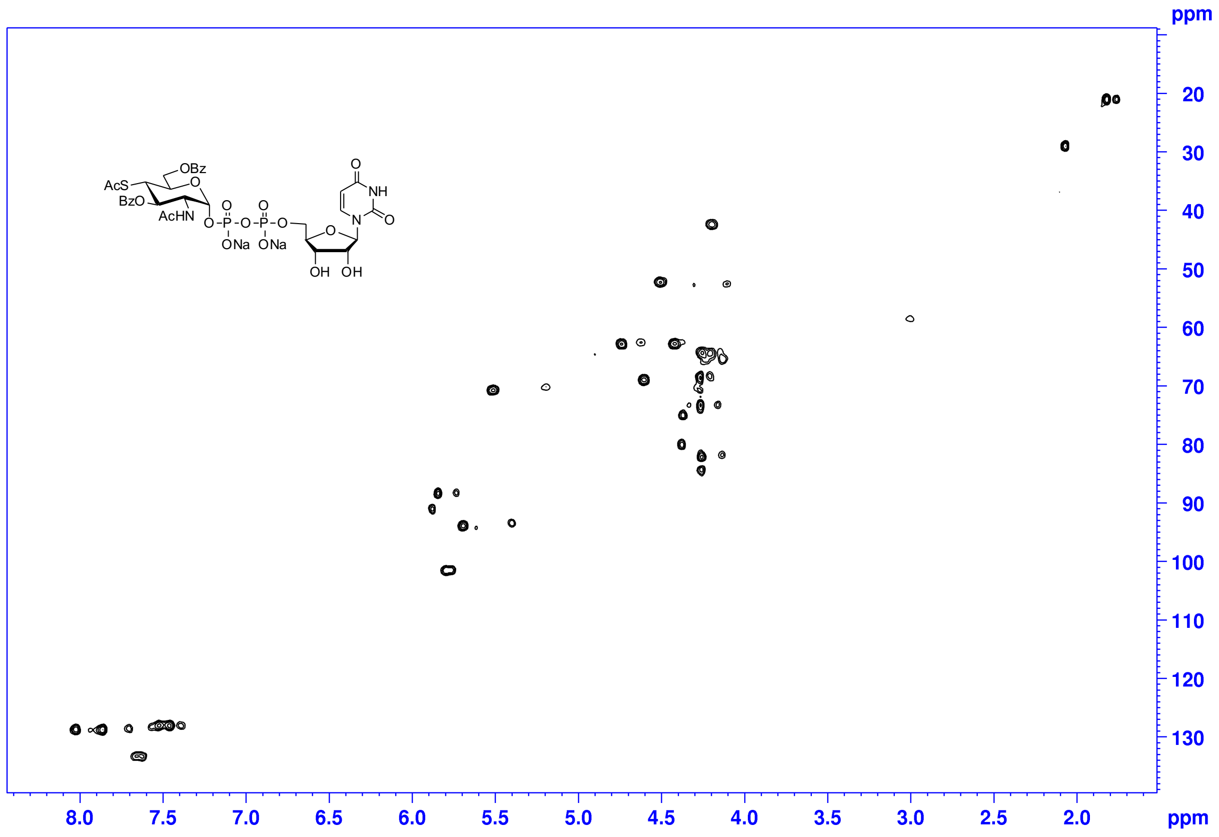

Supplementary Fig. 24. 2D HSQC NMR spectrum (D_2O) of compound **10**.

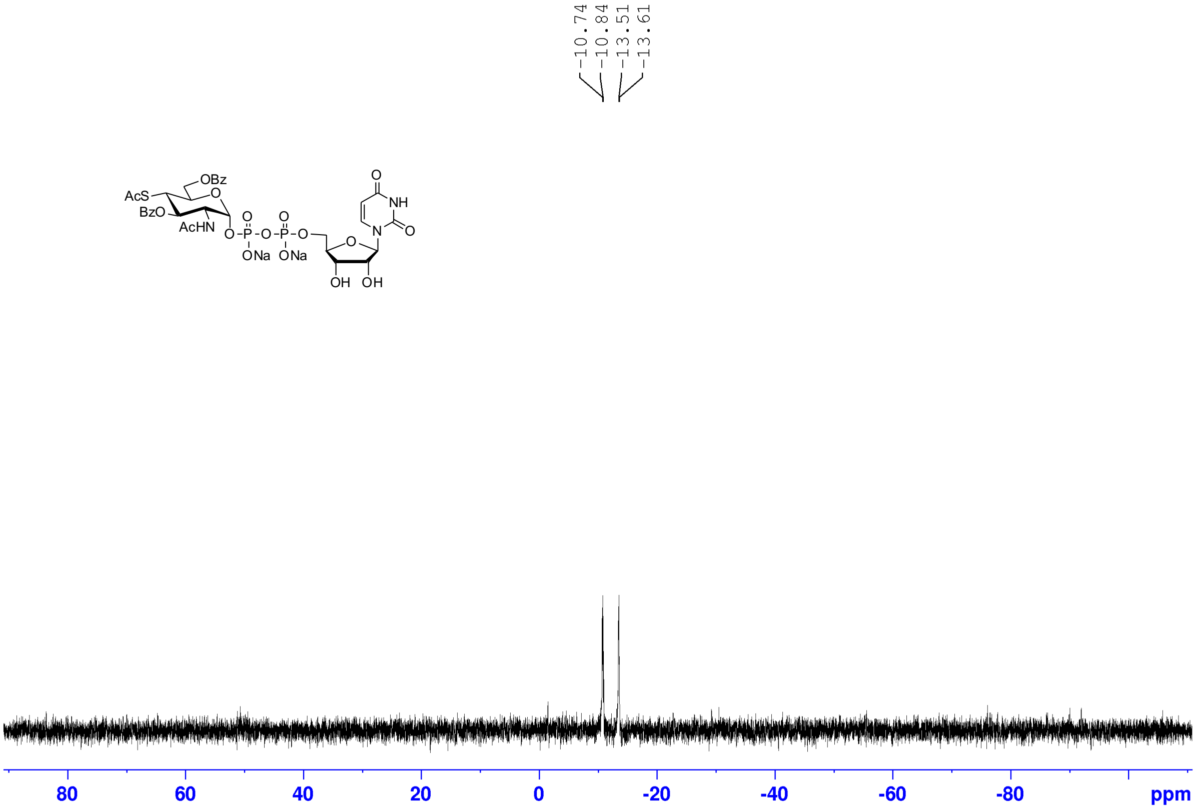

Supplementary Fig. 25. ^{31}P NMR spectrum (243 MHz, D_2O) of compound **10**.

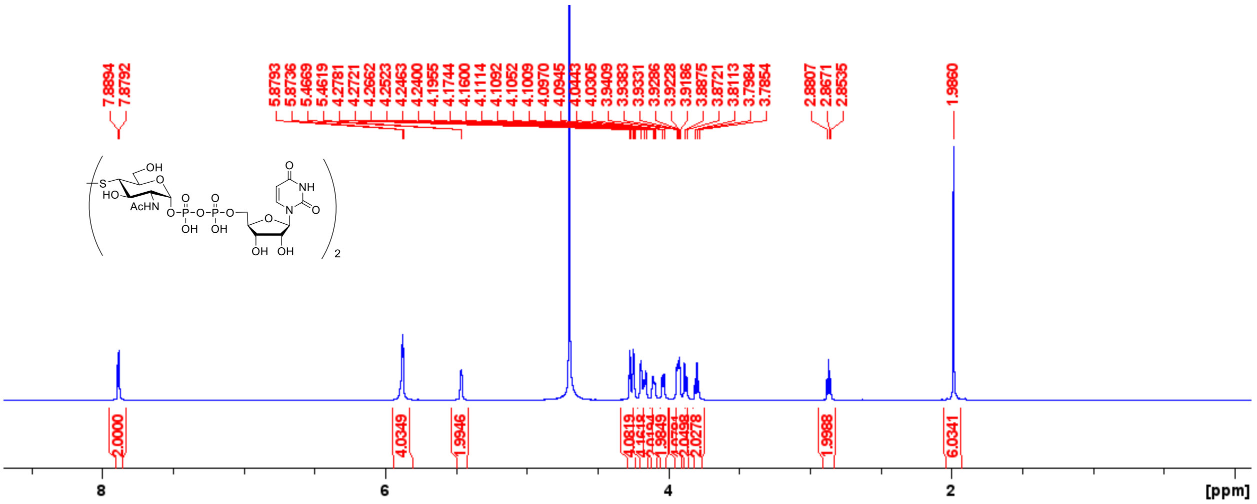

Supplementary Fig. 26. ^{1}H NMR spectrum (800 MHz, D_2O) of compound **11**.

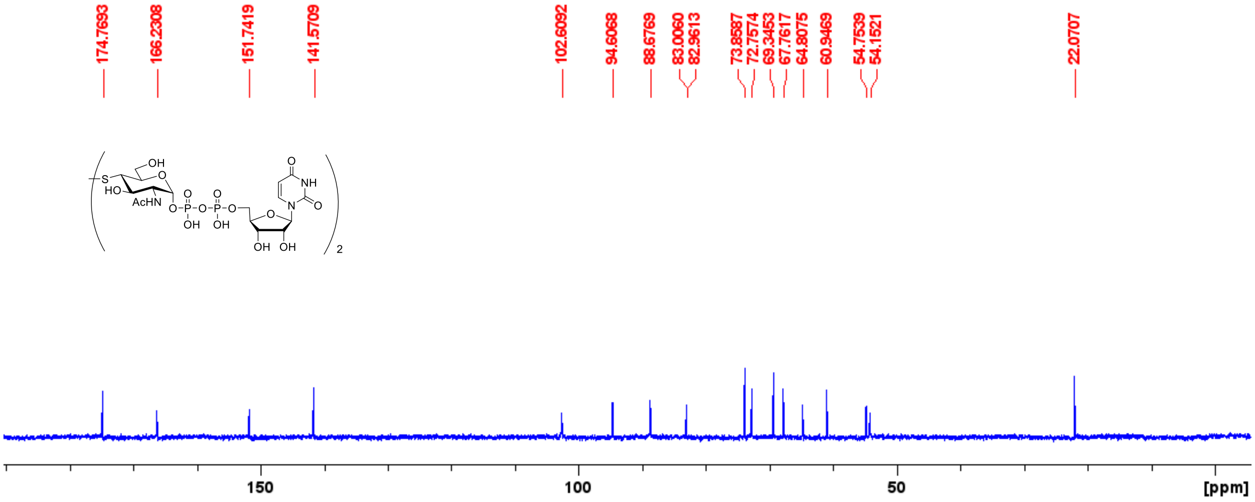

Supplementary Fig. 27. ^{13}C NMR spectrum (200 MHz, D_2O) of compound **11**.

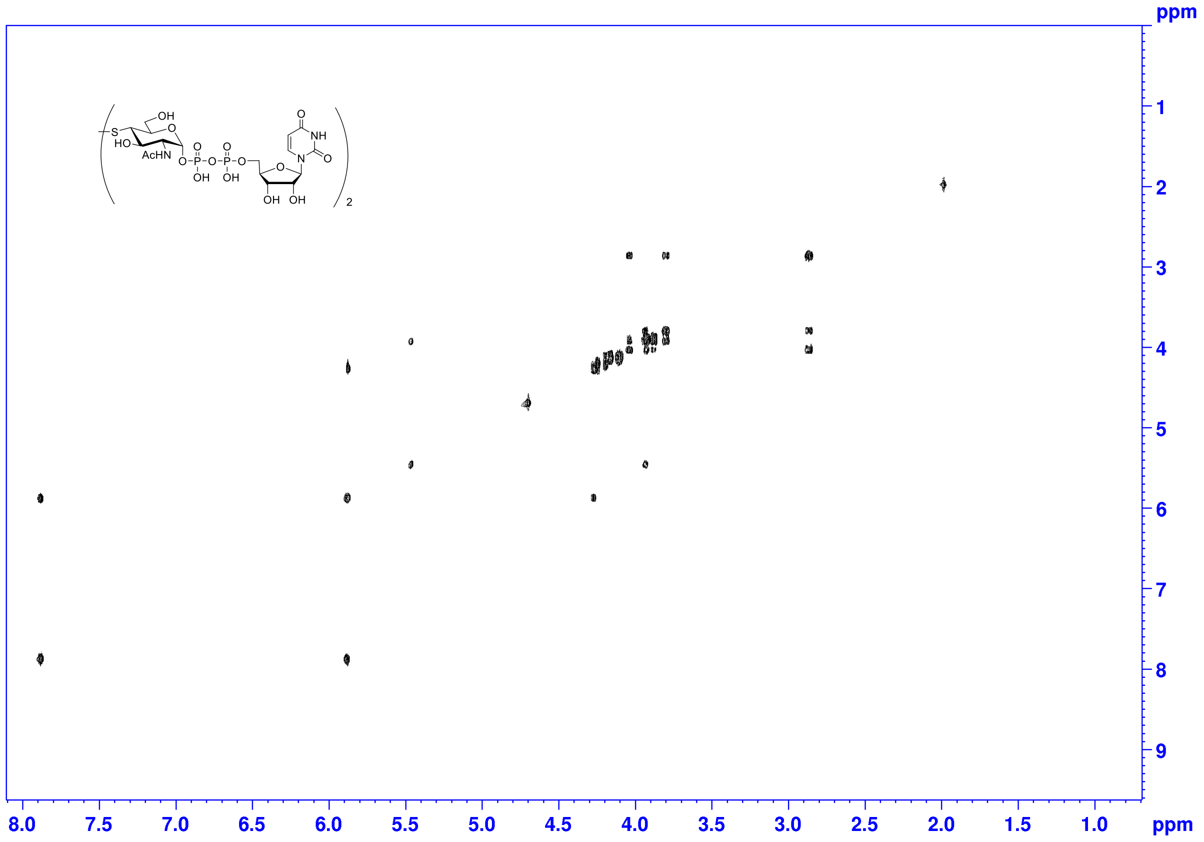

Supplementary Fig. 28. 2D COSY NMR spectrum (800 MHz, D_2O) of compound **11**.

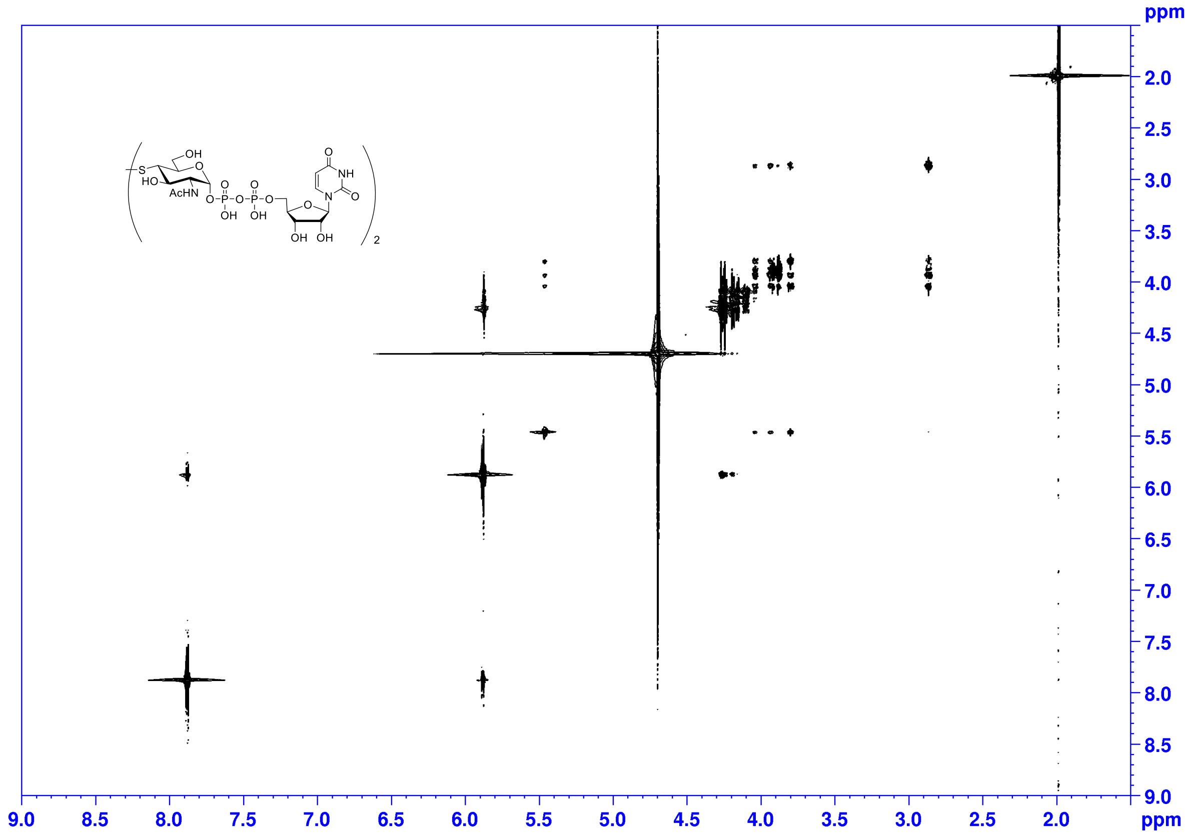

Supplementary Fig. 29. 2D TOCSY NMR spectrum (800 MHz, D_2O) of compound **11**.

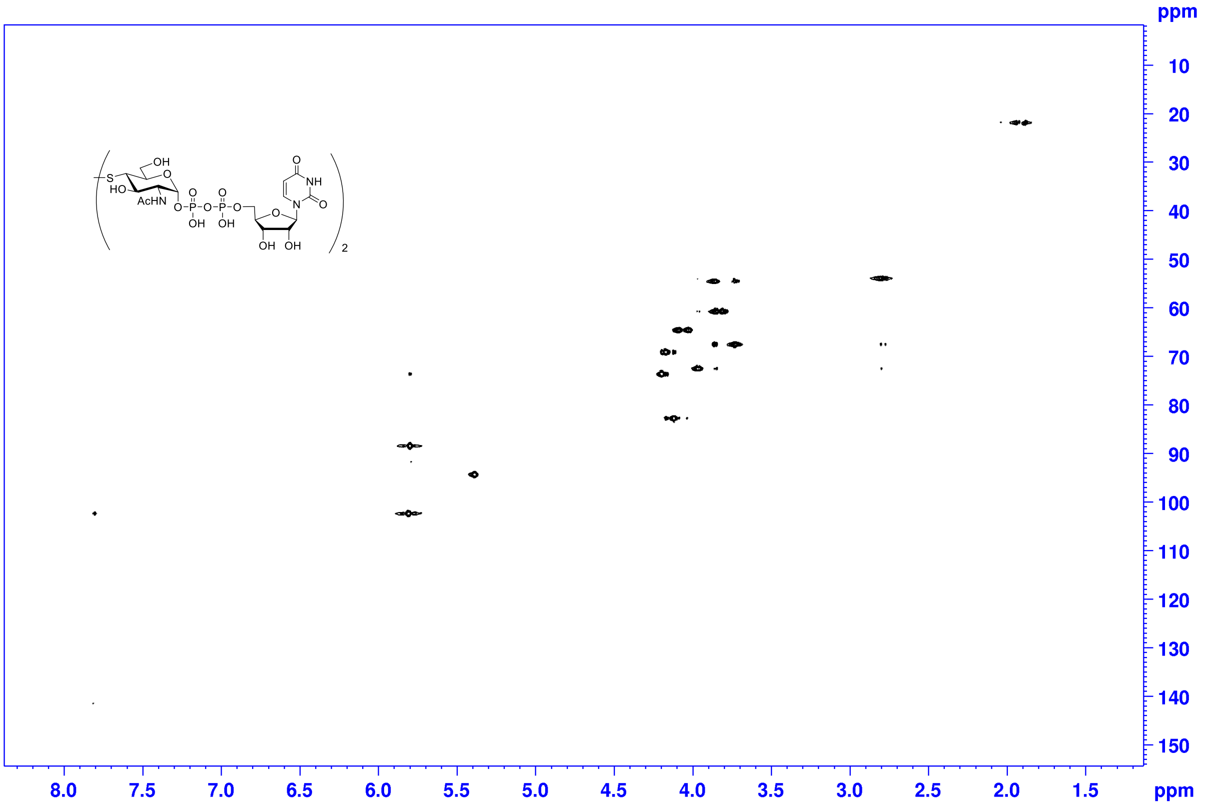

Supplementary Fig. 30. 2D HSQC NMR spectrum (D_2O) of compound **11**.

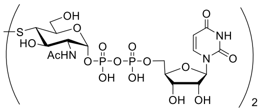

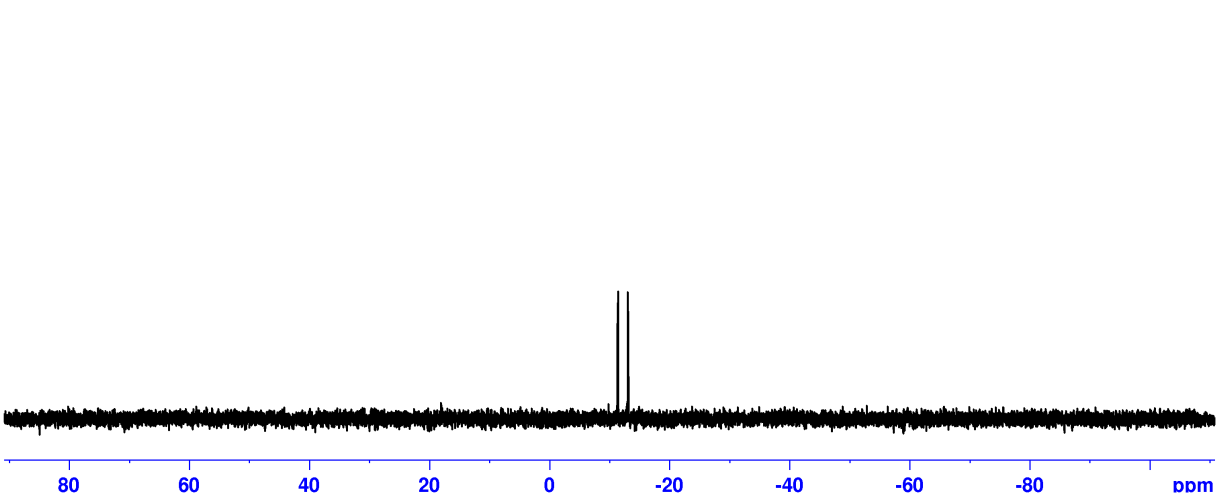

Supplementary Fig. 31. ^{31}P NMR spectrum (243 MHz, D_2O) of compound **11**.

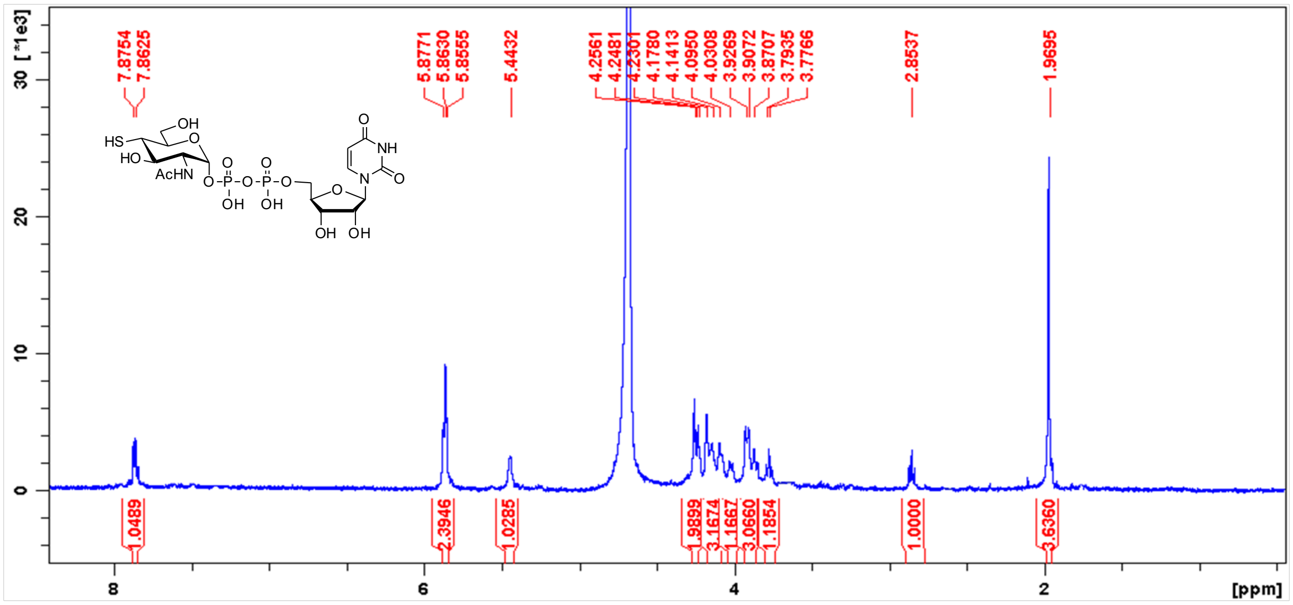

Supplementary Fig. 32. ^{1}H NMR spectrum (600 MHz, D_2O) of compound **12**.

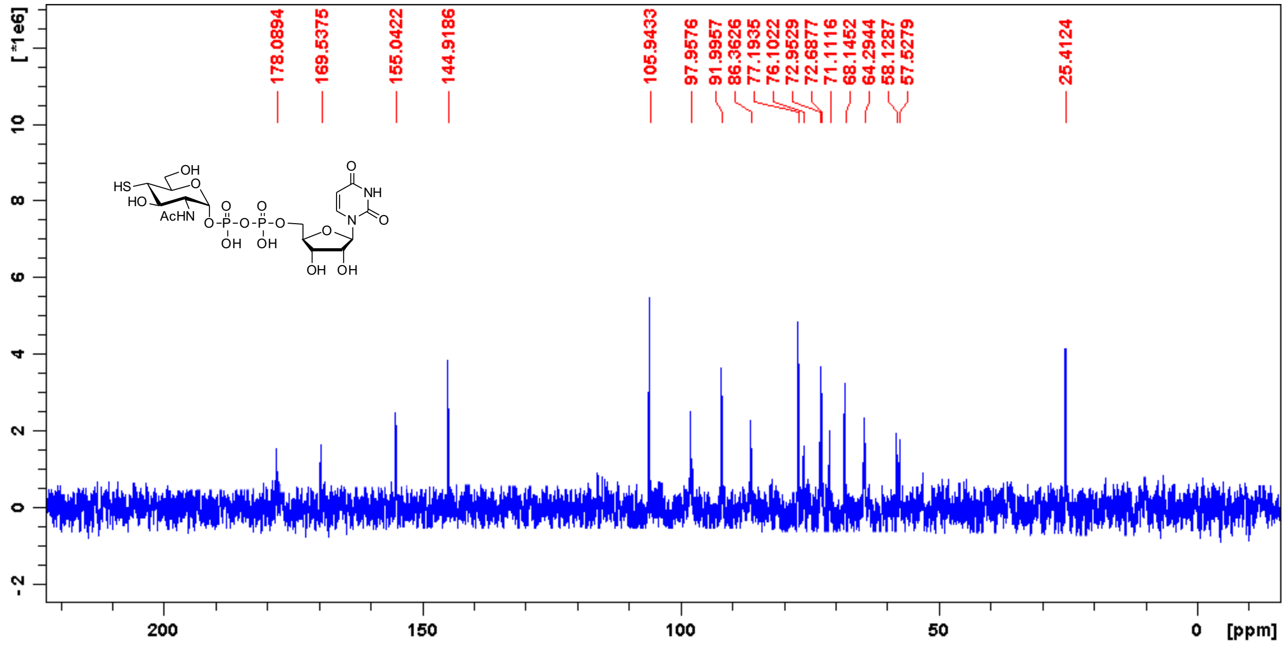

Supplementary Fig. 33. ^{13}C NMR spectrum (150 MHz, D_2O) of compound **12**.

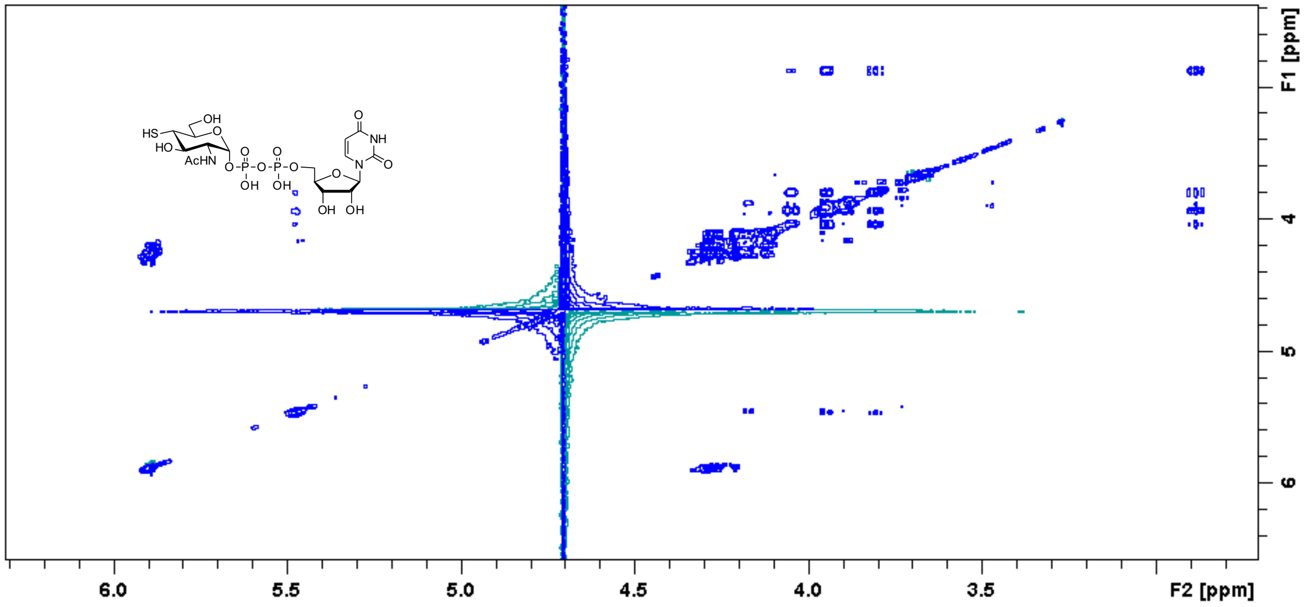

Supplementary Fig. 34. 2D TOCSY NMR spectrum (600 MHz, D_2O) of compound **12**.

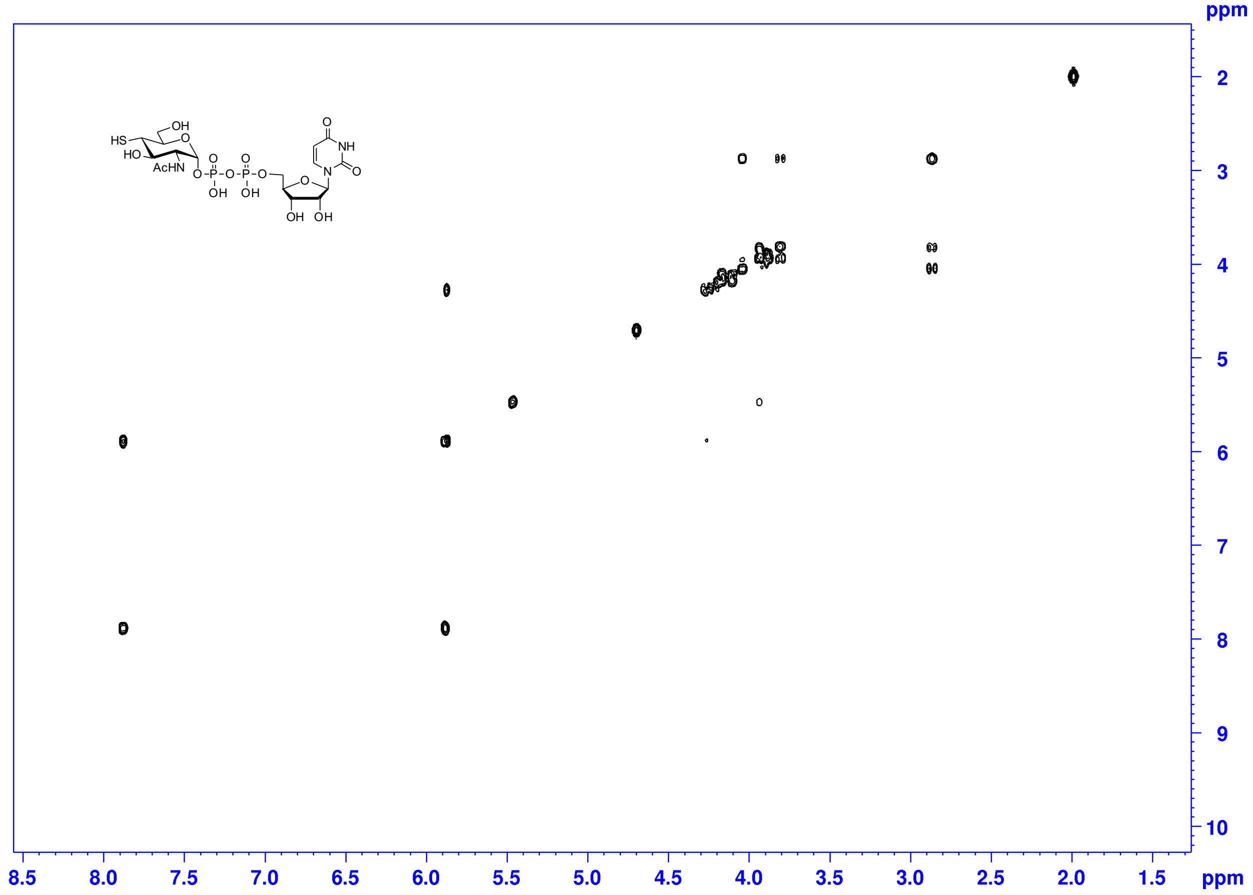

Supplementary Fig. 35. 2D COSY NMR spectrum (600 MHz, D_2O) of compound **12**.

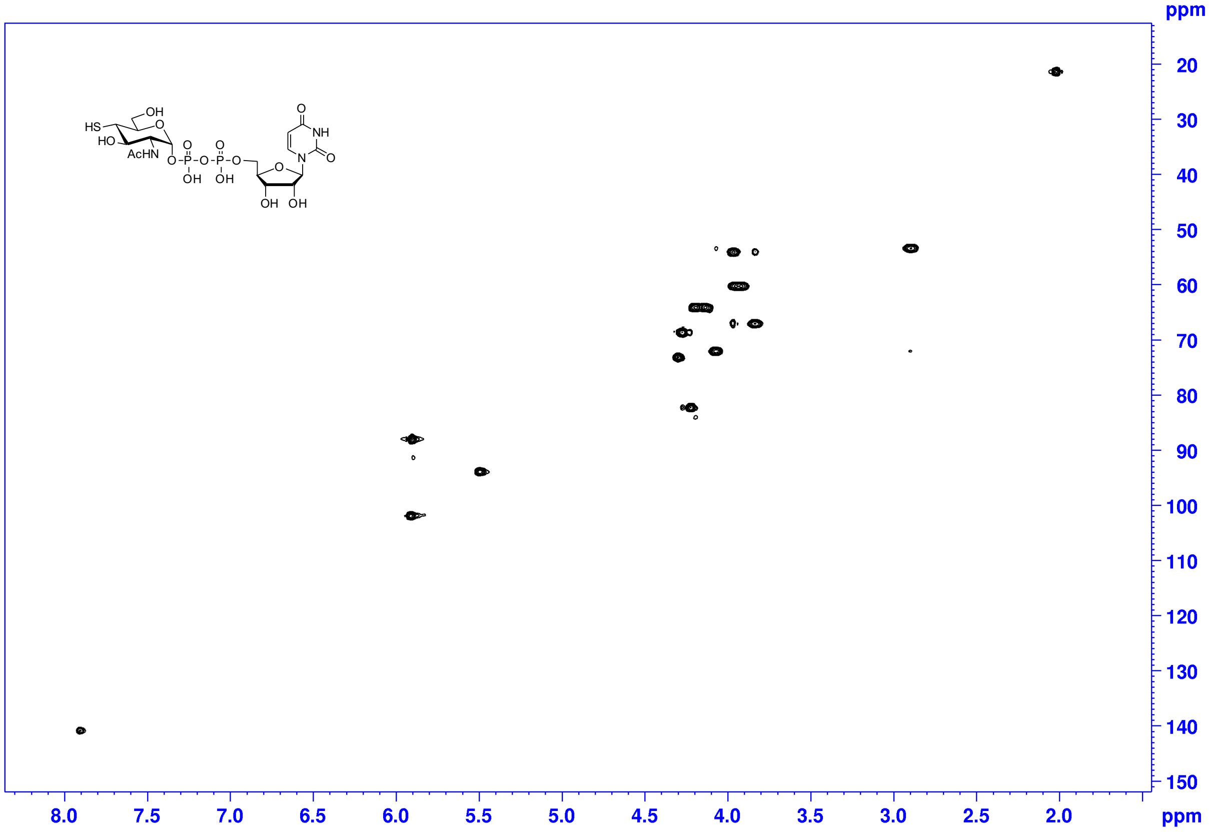

Supplementary Fig. 36. 2D HSQC NMR spectrum (D_2O) of compound **12**.

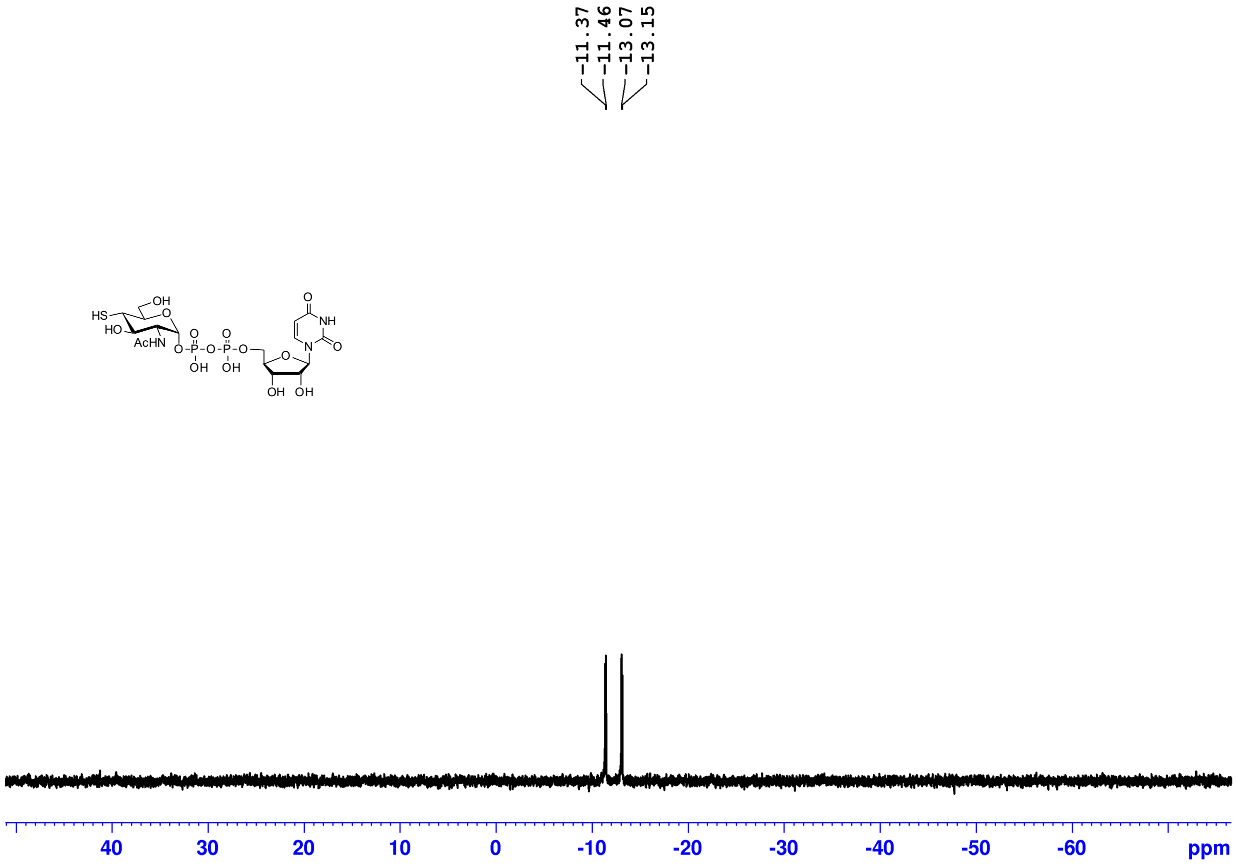

Supplementary Fig. 37. ^{31}P NMR spectrum (243 MHz, D_2O) of compound **12**.

a.

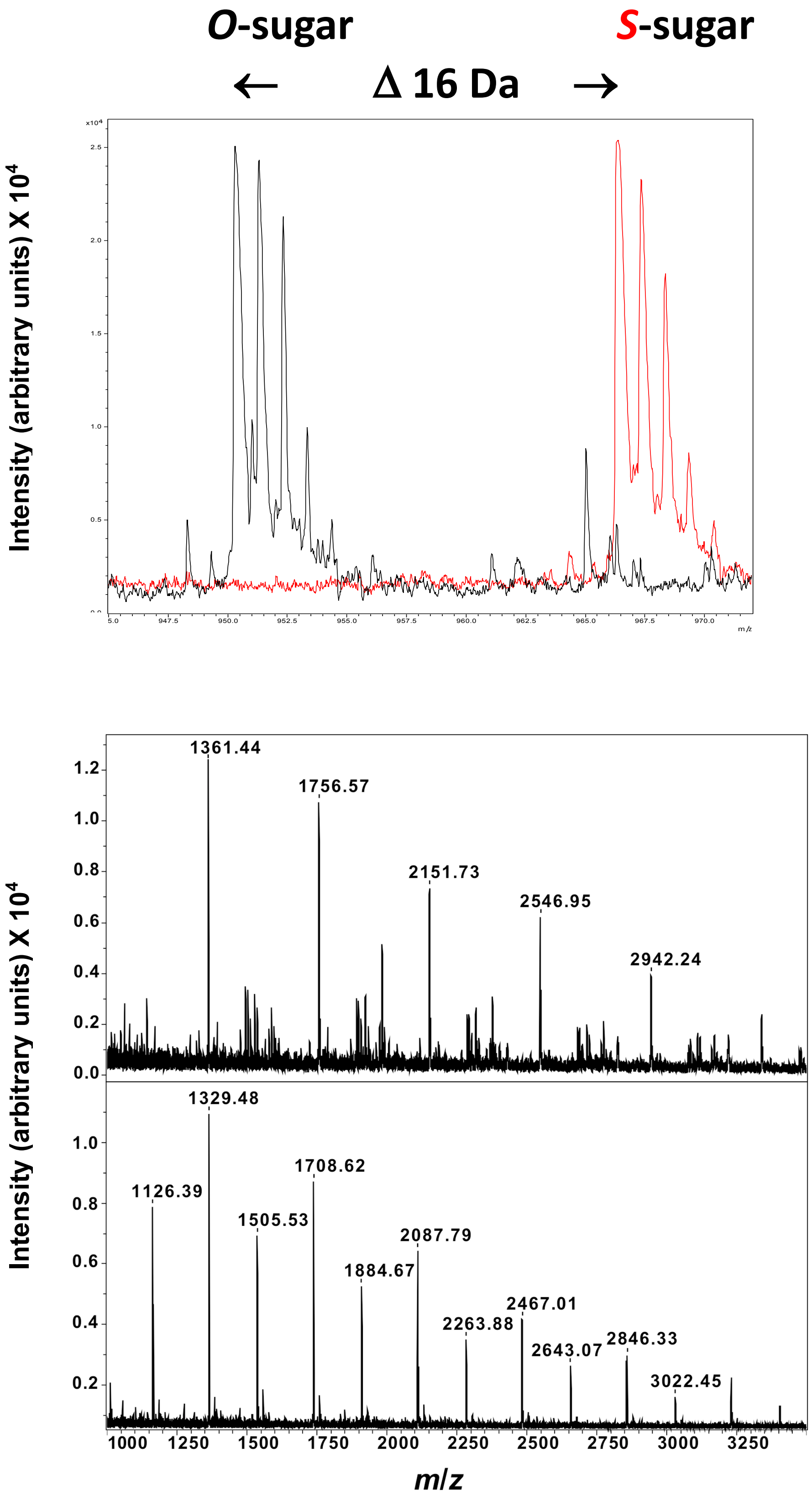
O-sugar
S-sugar
Δ 16 Da
Intensity (arbitrary units) X 10^4
1361.44
1756.57
2151.73
2546.95
2942.24
1329.48
1126.39
1505.53
1708.62
1884.67
2087.79
2263.88
2467.01
2643.07
2846.33
3022.45
m/z

Supplementary Fig. 38. MALDI-ToF MS analysis of *O*-linked and hemi-A *S*-linked heparosan in extension reactions.

a. Tetrasaccharides were made by extension of a heparosan-trimer (R'= GlcA-GlcNAc-GlcA-C_2H_4-amido-benzaldehyde) with either natural UDP-GlcNAc (*O*; *black*) or thiol-analog (*S*; *red*) as evidenced by the ~16 Da mass shift corresponding to the difference in sulfur versus oxygen.

b. The heparosan synthase created polymer chains when incubated with UDP-GlcA and either UDP-4-SH-GlcNAc analog (*top*) or native UDP-GlcNAc (*bottom*). The *S*-linked polymers have ~16 Da extra mass per disaccharide unit.

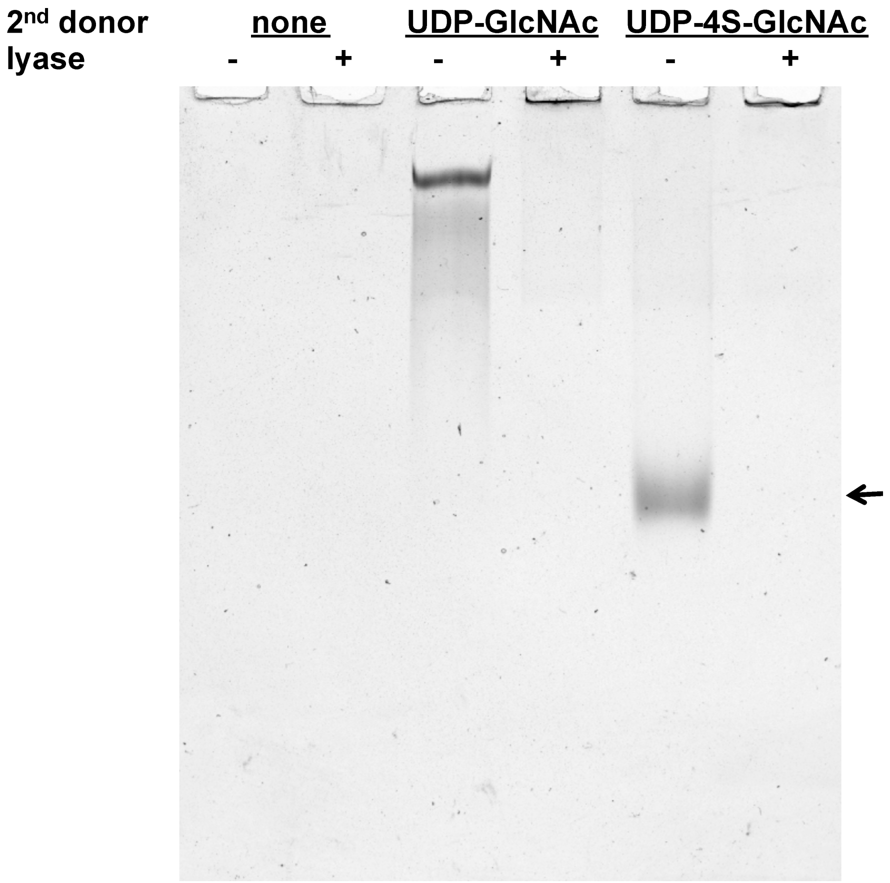

Supplementary Fig. 39. PAGE gel (6%) with Alcian Blue/silver staining of the reaction products made by a heparosan synthase with various UDP-sugar donors and an acceptor, heparosan tetrasaccharide ([GlcA-GlcNAc]$_2$).

As a negative control for co-polymerization, a reaction with only UDP-GlcA (no 2nd donor, *none*) is shown; no polymer is produced unless both UDP-sugar donors are simultaneously present. The reaction containing both natural donors, UDP-GlcNAc and UDP-GlcA, forms the naturally occurring *O*-linked heparosan (*n*=1; note: proof of polymerization or lyase digestion also shown in Fig. 4b, Supp. Fig. 40). Using the 4-thiol-GlcNAc analog and UDP-GlcA forms the unnatural hemi-A *S*-linked heparosan (marked with *arrow*). Both polymers were digested by bacterial heparin lyase III (+ *lanes*) thus allowing disaccharide mapping the sulfation modification state (Supplementary Fig. 24-27). In the hemi-A *S*-linked heparosan, the cleavage occurs at the *O*-linkage, namely GlcNAc-*O*-GlcA (*note*: the *S*-link resides between the other disaccharide unit, GlcA-*S*-GlcNAc, is the site of action of human heparanase, but not the bacterial lyase; *see* Fig. 4a).

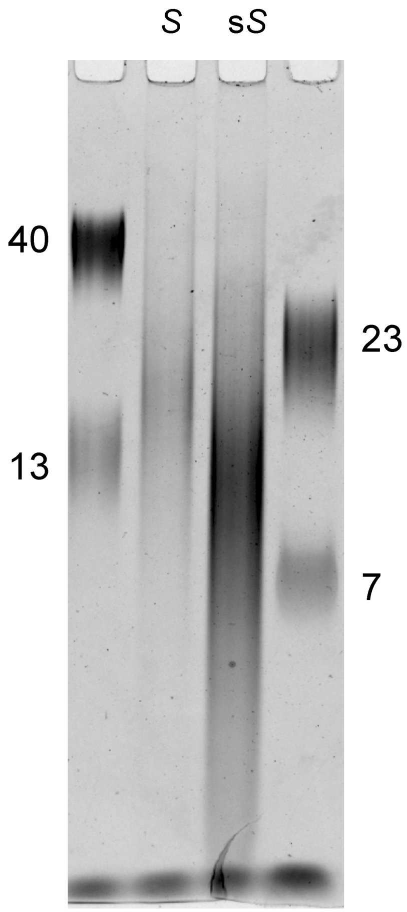

Supplementary Fig. 40. Determination of hemi-A *S*-linked heparosan analog average molecular weight (MW).

Heparosan standards with known MW (first and last lanes; *numerals* indicate average size in kDa as measured by multi-angle light scattering) were run on a 10% PAGE gel and compared to the *S*-linked heparosan (***S***) and chemically sulfated *S*-linked heparosan (**s*S***) made in preparative scale reactions (*n*=1). Sulfation increases the charge density thus the s*S* polymer migrates faster than the parent polymer as well as results in more intense staining.

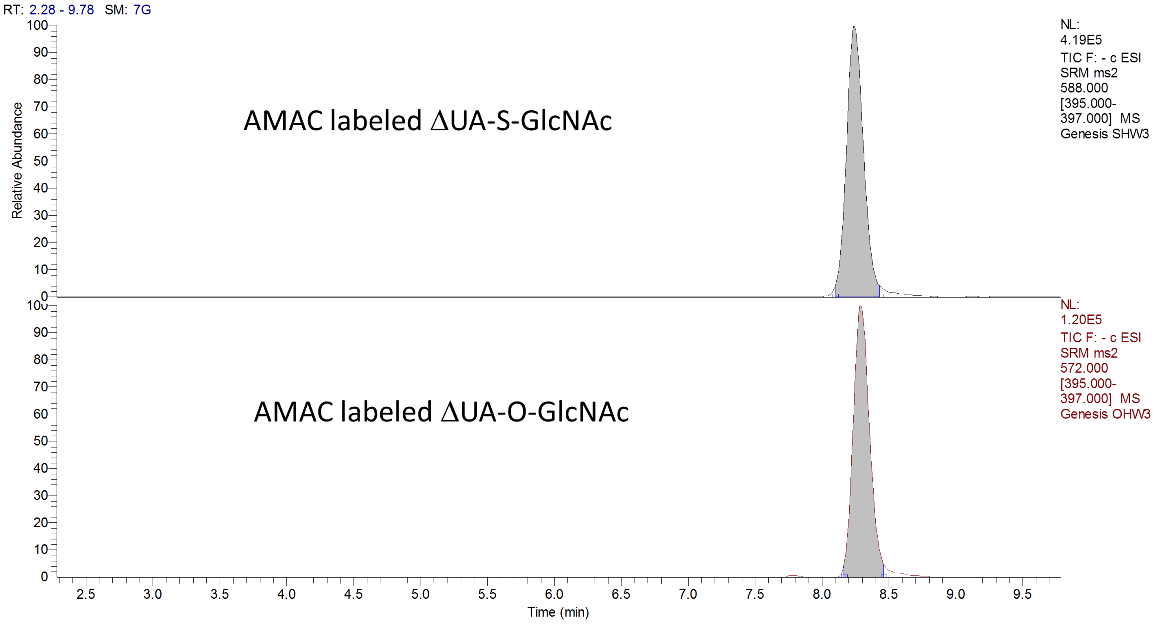

Supplementary Fig. 41. Cleavage of *S*-linked and *O*-linked heparosan with bacterial heparin lyases.

Disaccharide generated from heparin lyase treatment of *S*-linked and *O*-linked heparosan was monitored by LC-MS/MS using MRM after AMAC labeling. Ion transition *m/z* 588/396 and *m/z* 572/396, corresponding to ΔUA-*S*-GlcNAc and ΔUA-*O*-GlcNAc, respectively.

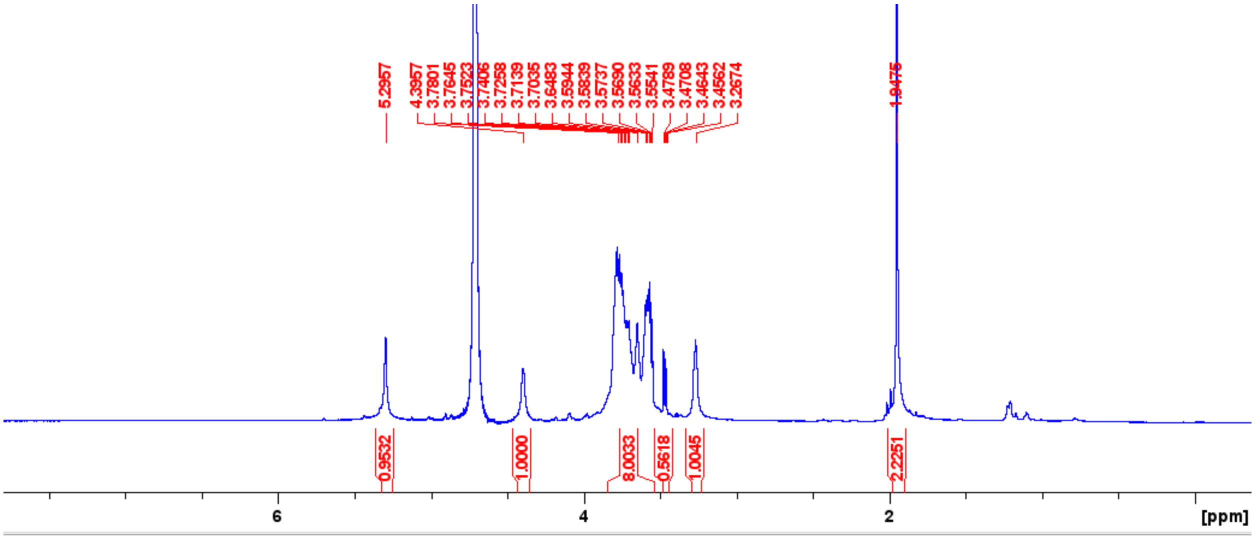

Supplementary Fig. 42. ^{1}H NMR spectrum (800 MHz, D_2O) of the natural *O*-linked heparosan standard.

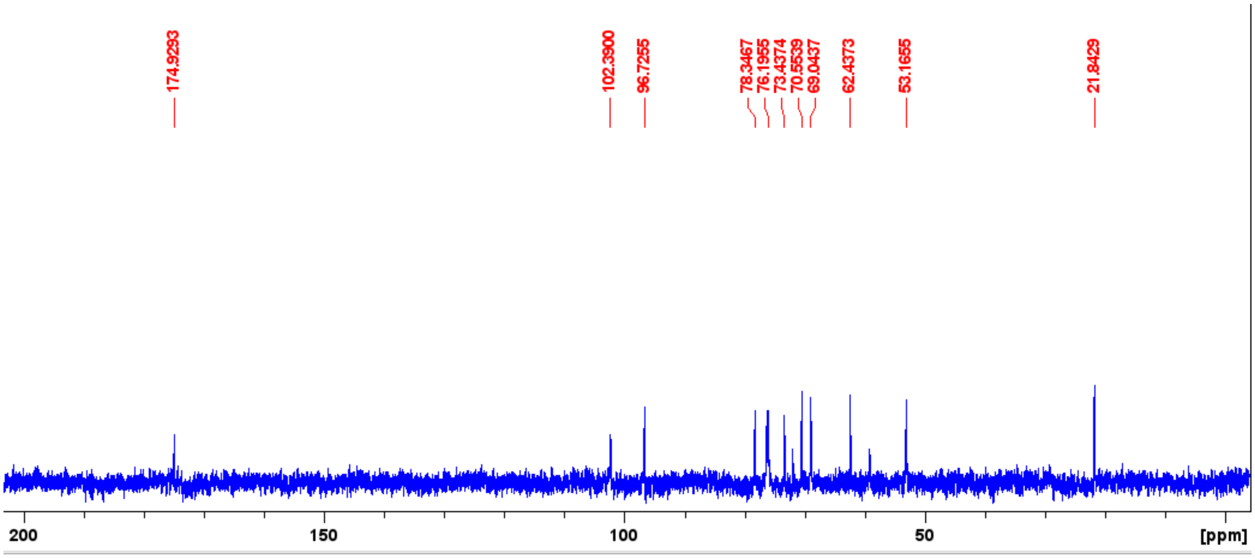

Supplementary Fig. 43. ^{13}C NMR spectrum (200 MHz, D_2O) of *O*-linked heparosan standard.

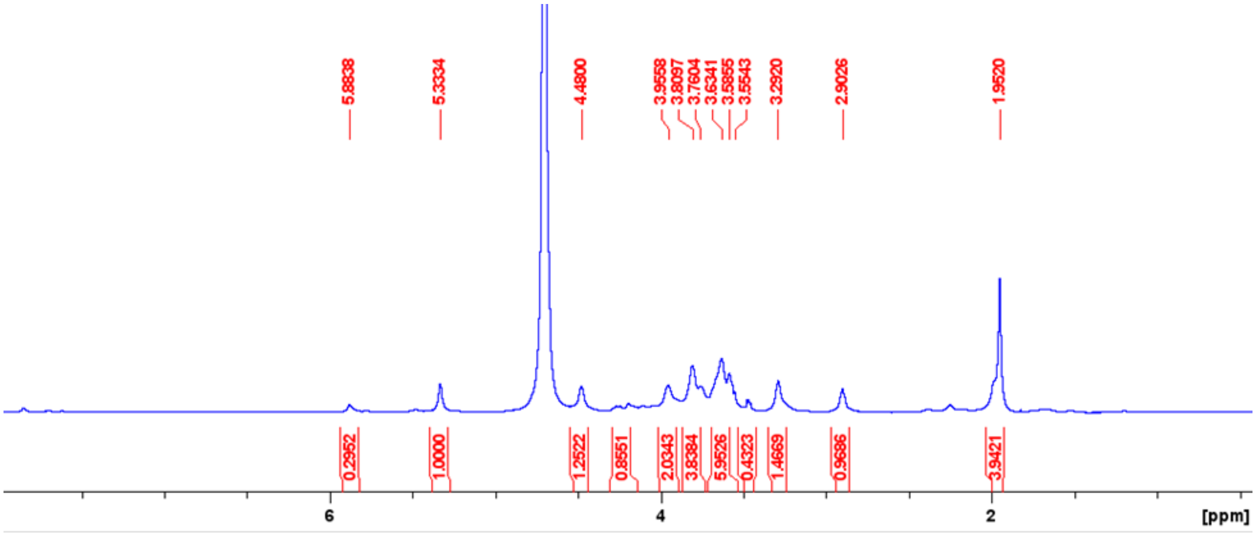

Supplementary Fig. 44. ^{1}H NMR spectrum (800 MHz, D_2O) of *S*-linked heparosan polysaccharide.

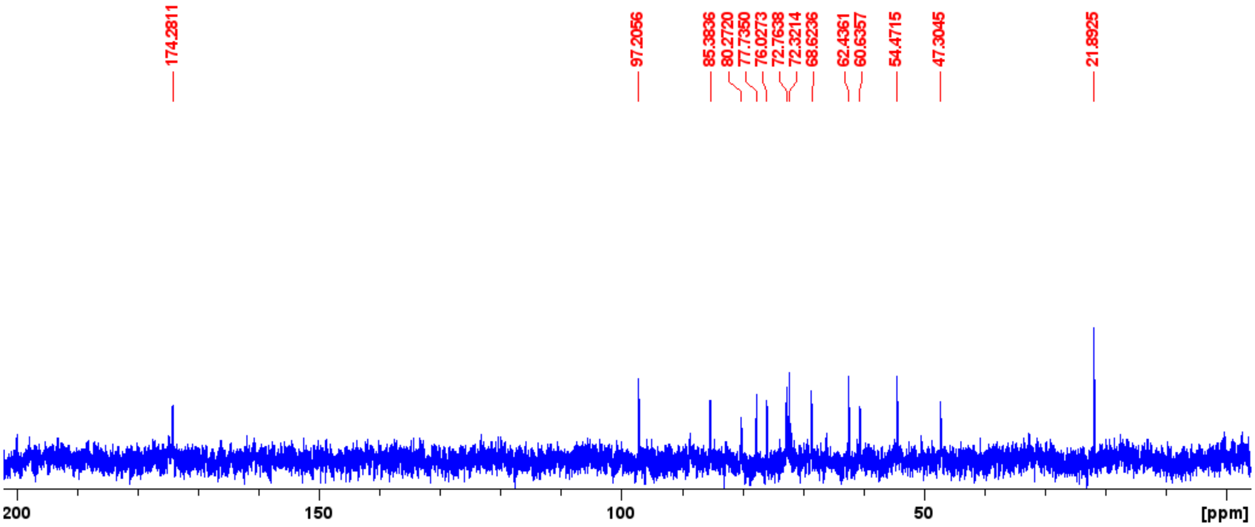

Supplementary Fig. 45. ^{13}C NMR spectrum (200 MHz, D_2O) of *S*-linked heparosan polysaccharide.

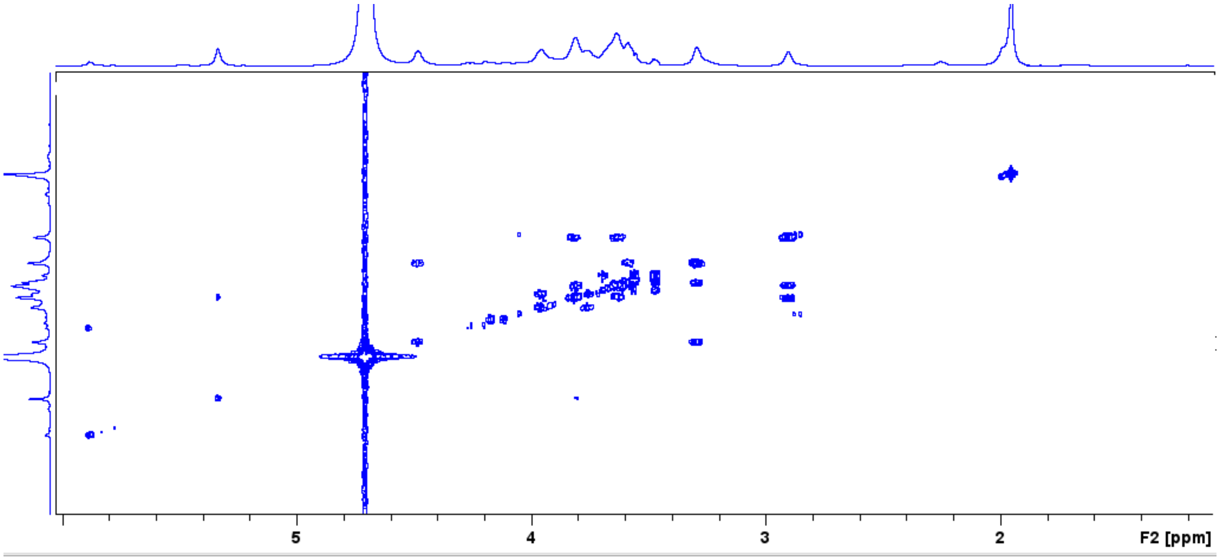

Supplementary Fig. 46. 2D COSY NMR spectrum (800 MHz, D_2O) of *S*-linked heparosan polysaccharide

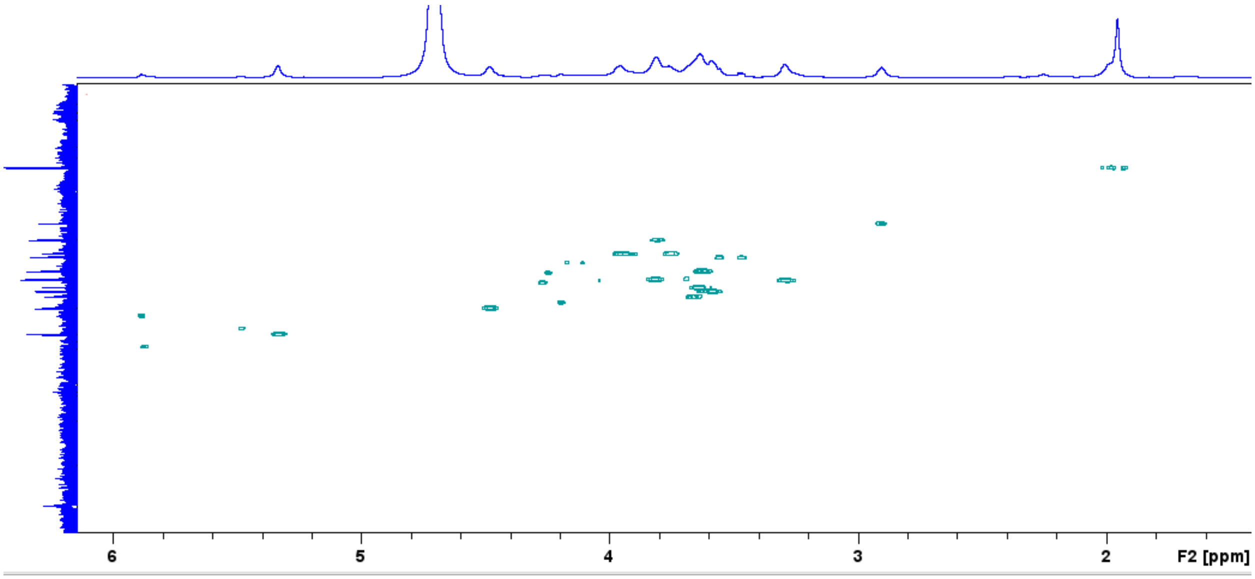

Supplementary Fig. 47. 2D HSQC NMR spectrum (800 MHz, D_2O) of *S*-linked heparosan polysaccharide.

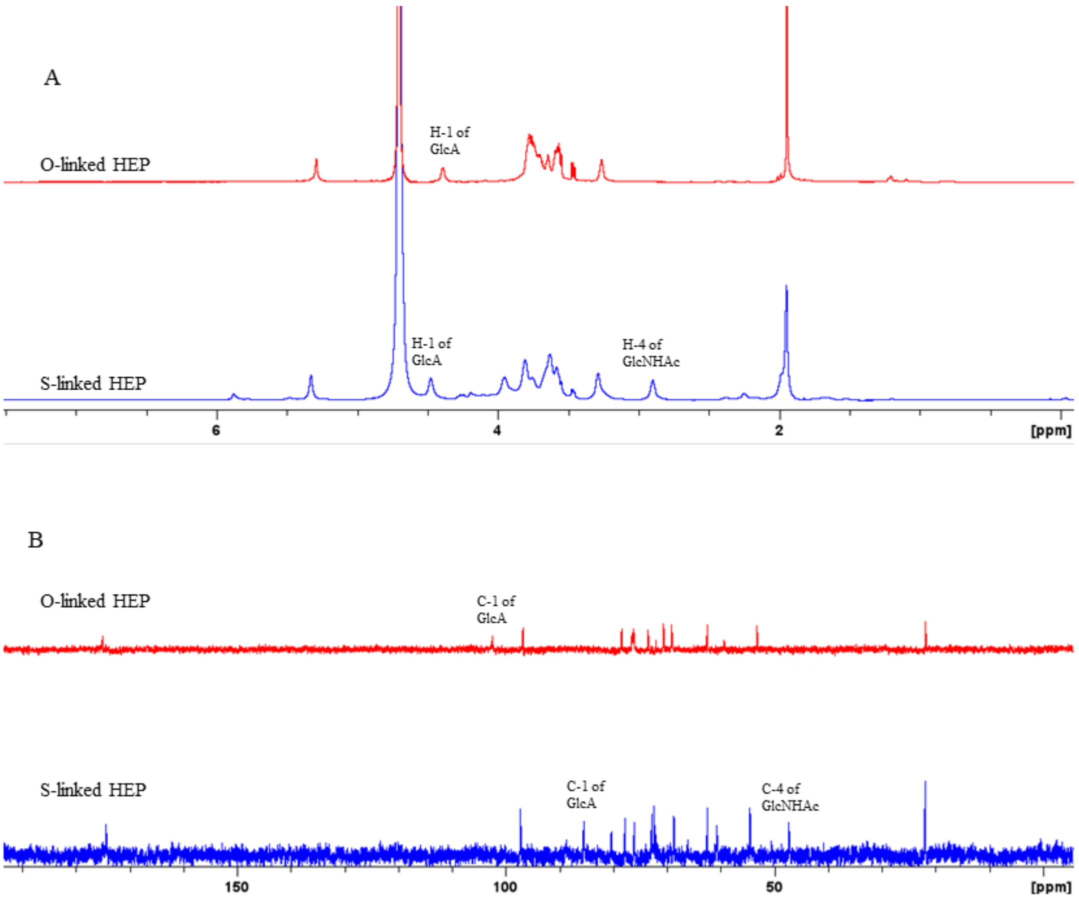

Supplementary Fig. 48. NMR analysis of *O*-linked heparosan standard and hemi-A *S*-linked heparosan.

(**A**) 1D ^{1}H NMR spectra of *O*-linked heparosan standard and hemi-A *S*-linked heparosan.

(**B**) 1D ^{13}C NMR spectra of *O*-linked heparosan standard and hemi-A *S*-linked heparosan.

MRM of HS Standard

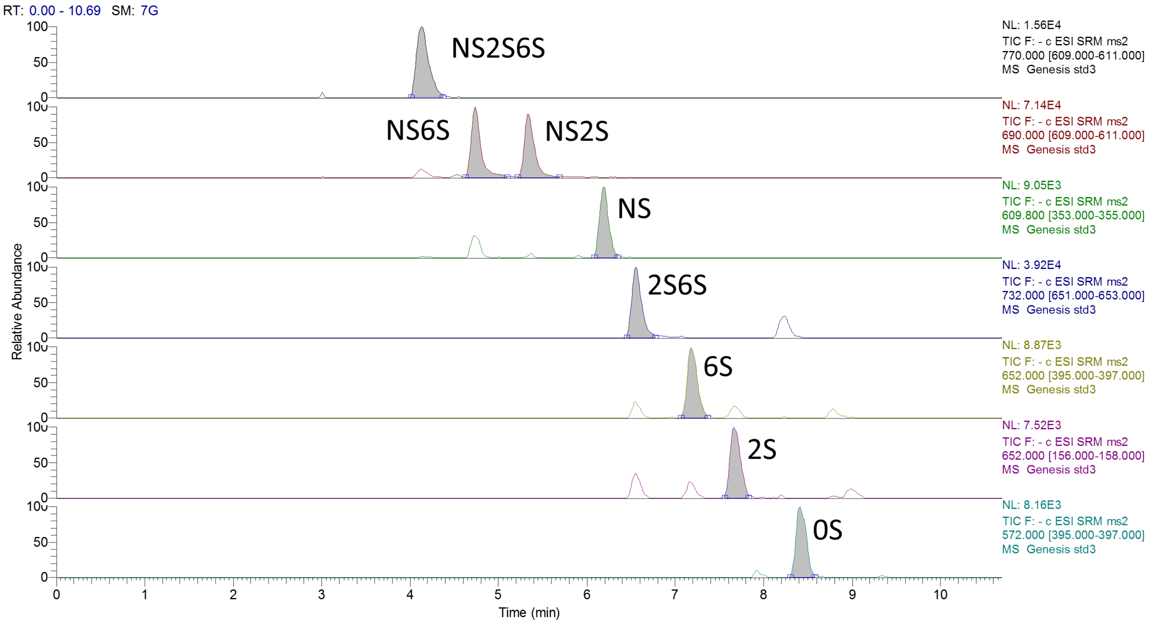

MRM of chemical synthesized S-linked-HS

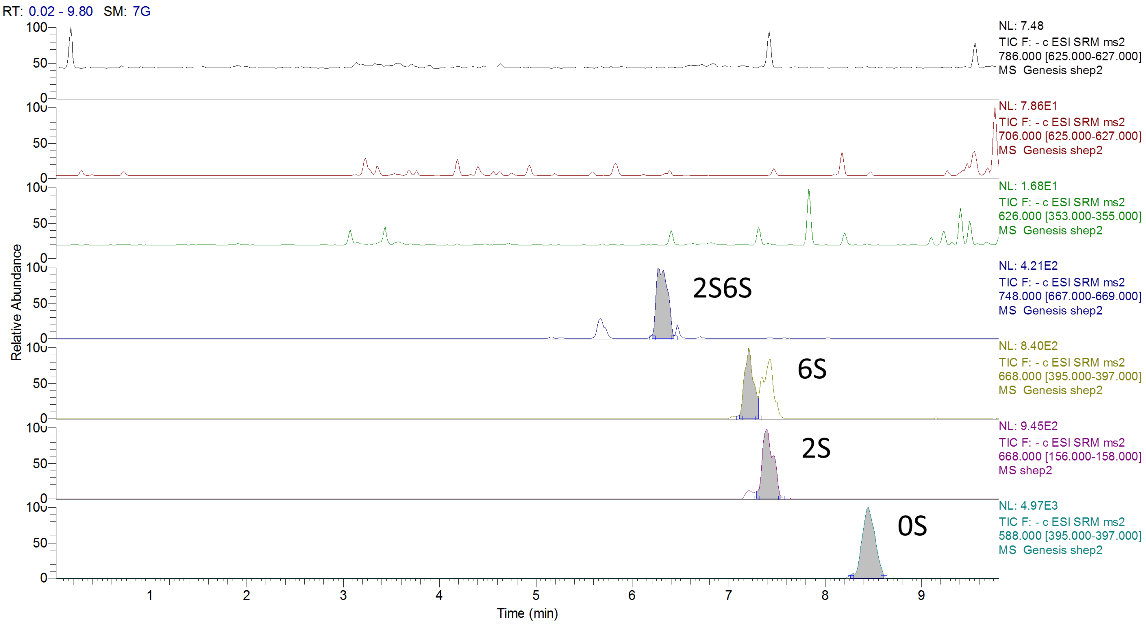

MRM of chemical synthesized O-linked-HS

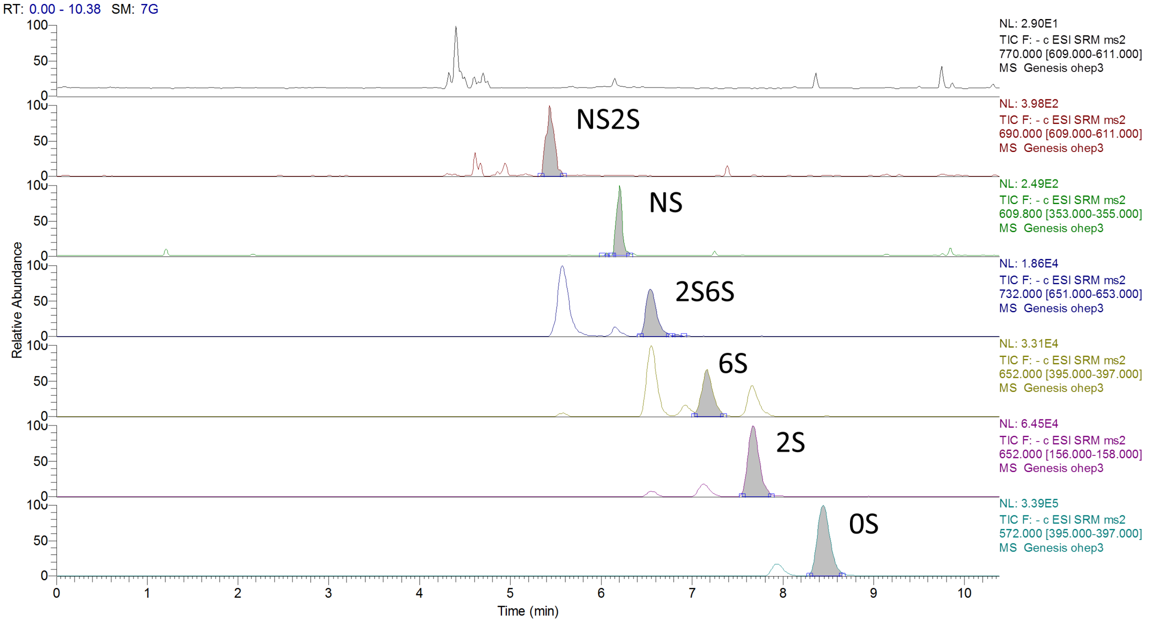

Supplementary Fig. 49. LC-MS/MS analysis of AMAC-labeled eight heparan sulfate disaccharides of chemically *O*-sulfated heparosan.

MRM ion chromatograms of AMAC-labeled disaccharides from equal molar disaccharide standard, chemically *O*-sulfated *S*-linked-HS analog, or chemically *O*-sulfated *O*-linked-HS. TriS, NS6S, NS2S and NSH are below the limit of detection (LOD) and are reported as 0.0%. The amounts of the various disaccharides are shown in Supplementary Fig. 50.

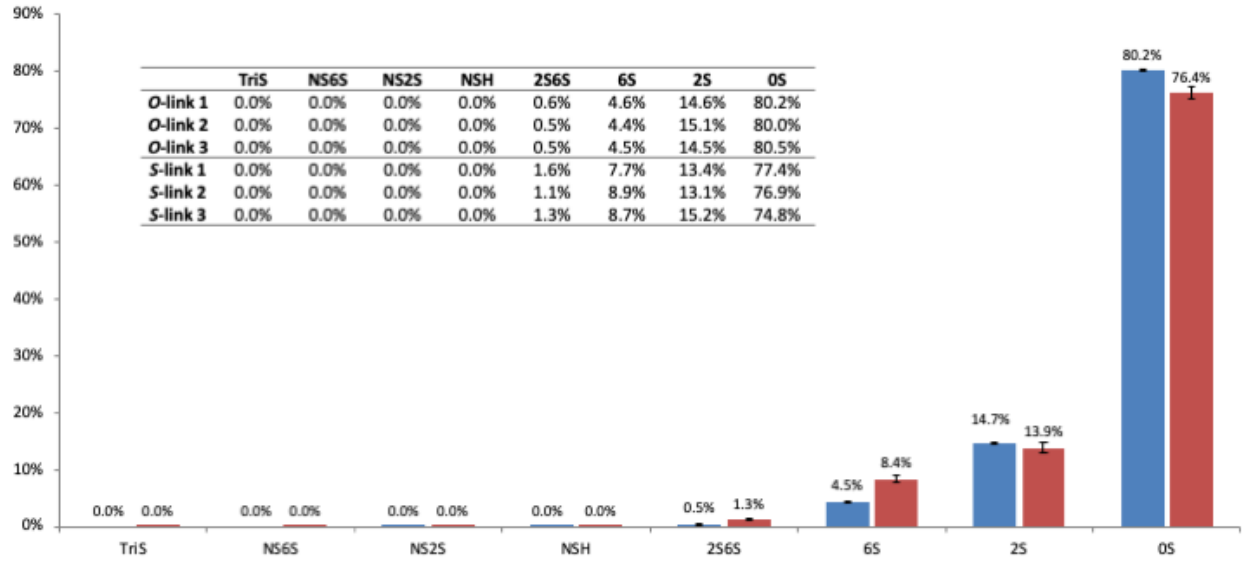

	TriS	NS6S	NS2S	NSH	2S6S	6S	2S	0S
***O*-link 1**	0.0%	0.0%	0.0%	0.0%	0.6%	4.6%	14.6%	80.2%
***O*-link 2**	0.0%	0.0%	0.0%	0.0%	0.5%	4.4%	15.1%	80.0%
***O*-link 3**	0.0%	0.0%	0.0%	0.0%	0.5%	4.5%	14.5%	80.5%
***S*-link 1**	0.0%	0.0%	0.0%	0.0%	1.6%	7.7%	13.4%	77.4%
***S*-link 2**	0.0%	0.0%	0.0%	0.0%	1.1%	8.9%	13.1%	76.9%
***S*-link 3**	0.0%	0.0%	0.0%	0.0%	1.3%	8.7%	15.2%	74.8%

Supplementary Fig. 50. MS disaccharide analysis of the chemically *O*-sulfated heparosans.

Blue bars represent chemically sulfated *O*-linked HS. Red bars represent chemically sulfated *S*-linked HS. The 2-*O*-sulfo-ΔUA-S-GlcNAc disaccharide was the major sulfated component (corresponding to chemical 2-O-sulfation; 13.9% +/- 0.94% or 14.7% +/- 0.23% mol% for *O*- vs *S*-link, respectively) with the next being ΔUA-GlcNAc6S (corresponding to chemical 6-*O*-sulfation; 4.5% +/- 0.10% or 8.4% +/- 0.57% mol% for *O*- vs S-link, respectively) in both the chemically sulfated *O*- and *S*-linked heparosan species. (*n*=3 independently prepared samples examined over *n*=3 independent tests, shown in inset table; bars depict averaged data +/- standard deviation; representative raw MRM results are shown in Supplementary Fig. 49).

MRM of HS Standard

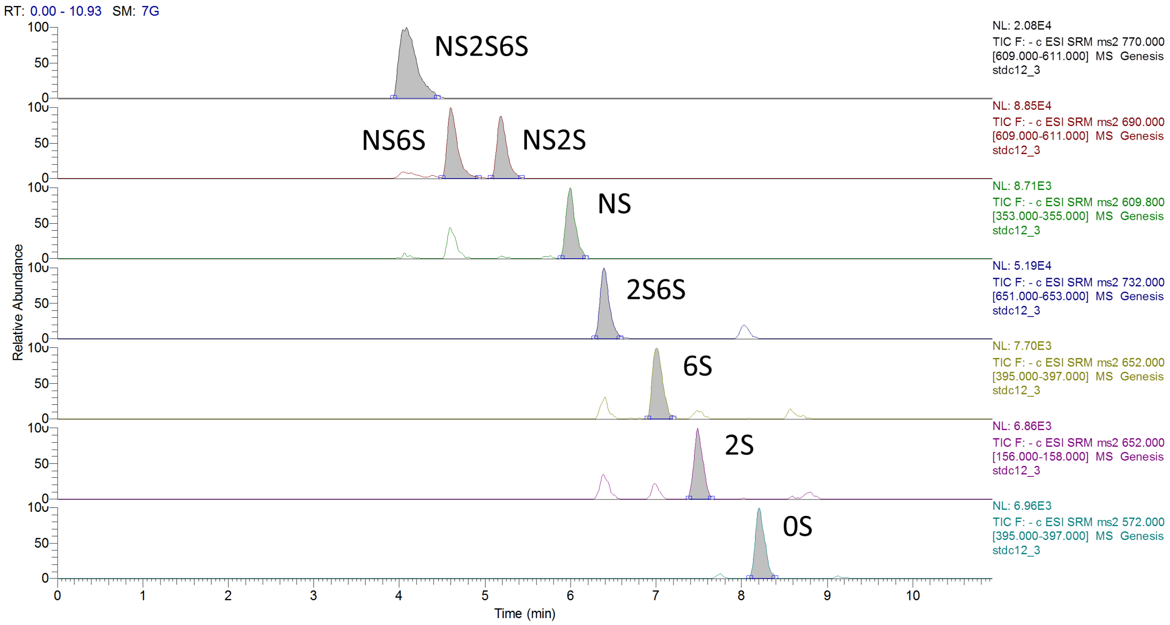

MRM of enzyme synthesized S-linked-HS

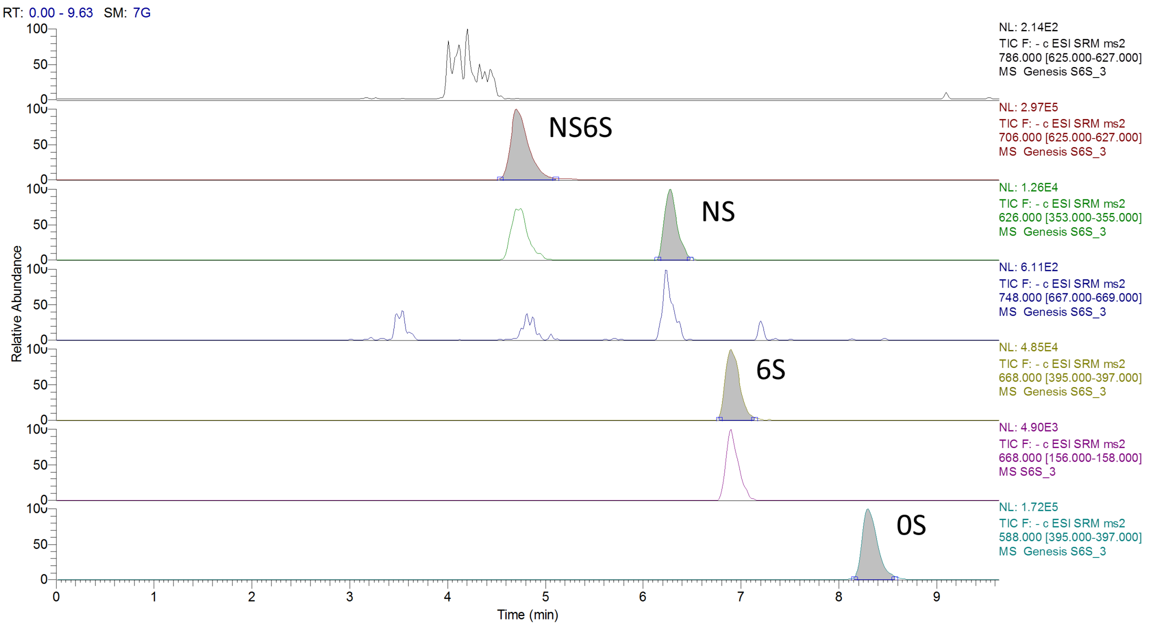

MRM of enzyme synthesized O-linked-HS

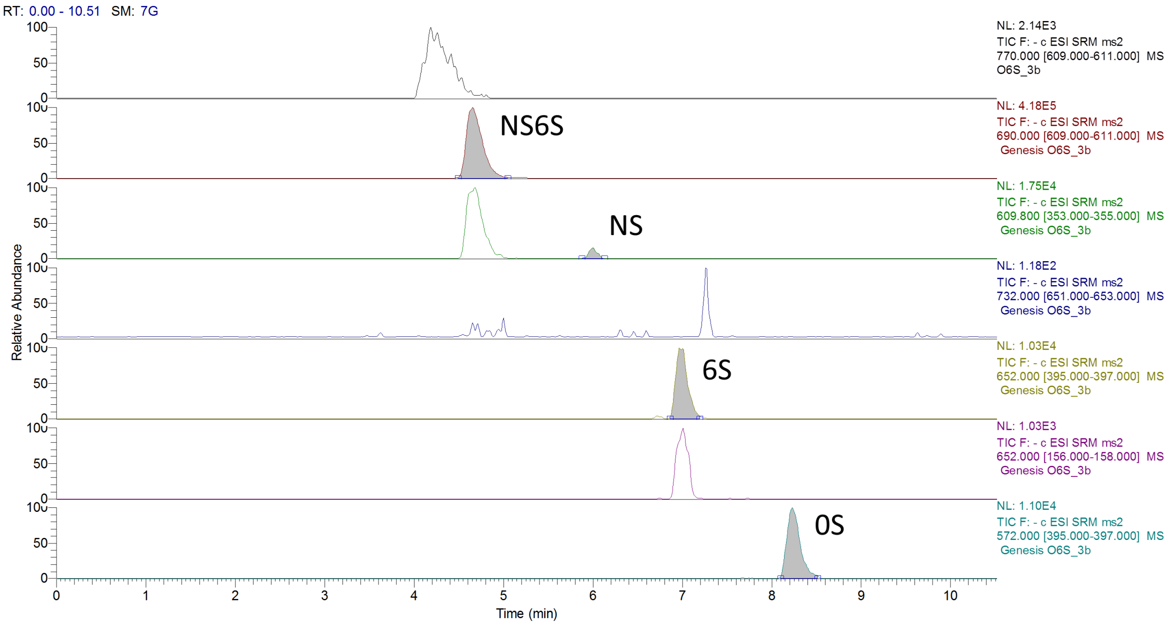

Supplementary Fig. 51. MS disaccharide analysis of NDST-treated *O*- and *S*-linked heparosan.

The amounts of the various sulfated isomers are presented in Supplementary Fig. 52.

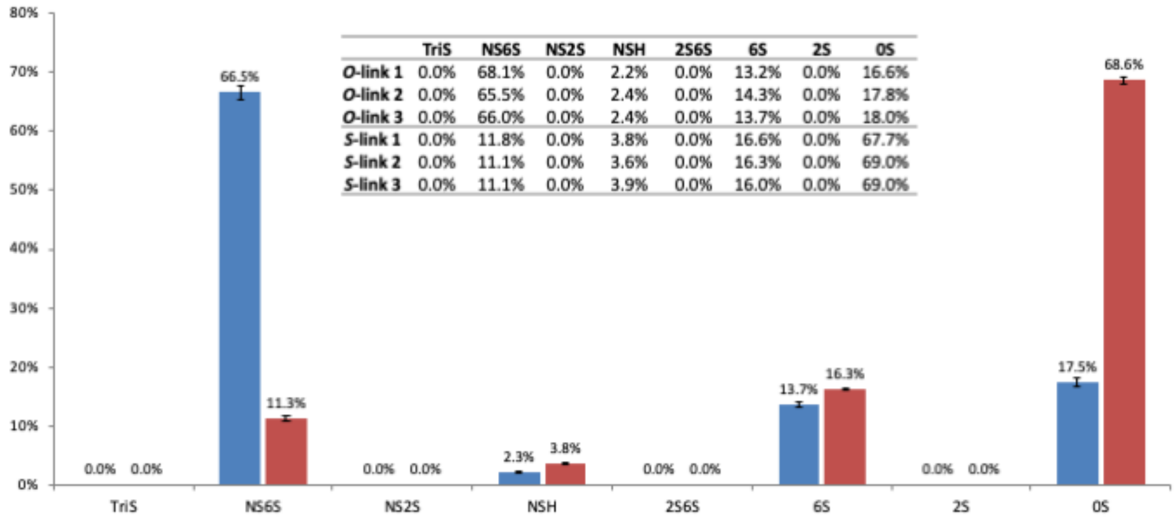

	TriS	NS6S	NS2S	NSH	2S6S	6S	2S	0S
***O*-link 1**	0.0%	68.1%	0.0%	2.2%	0.0%	13.2%	0.0%	16.6%
***O*-link 2**	0.0%	65.5%	0.0%	2.4%	0.0%	14.3%	0.0%	17.8%
***O*-link 3**	0.0%	66.0%	0.0%	2.4%	0.0%	13.7%	0.0%	18.0%
***S*-link 1**	0.0%	11.8%	0.0%	3.8%	0.0%	16.6%	0.0%	67.7%
***S*-link 2**	0.0%	11.1%	0.0%	3.6%	0.0%	16.3%	0.0%	69.0%
***S*-link 3**	0.0%	11.1%	0.0%	3.9%	0.0%	16.0%	0.0%	69.0%

Supplementary Fig. 52. MS disaccharide analysis of NDST- and 6*O*-ST-treated *O*- and *S*-linked heparosan.

Blue colored bars represent enzymatically sulfated *O*-linked HS. Red colored bars represent enzymatically sulfated *S*-linked HS. The hemi-A heparosan analog could be enzymatically converted, using *N*-deacetylase, *N*-sulfotransferase (NDST) to the *N*-sulfated-4-thio-HS analog. Disaccharide analyses showed that treatment with HS NDST and subsequent treatment with HS 6-*O*-sulfotransferase (6-OST) converted 15.1% +/- 0.47% (mol%) of the GlcNAc residues into $GlcNSO_3$ residues and resulted in 27.6% +/- 0.62% 6-*O*-sulfation affording the *N*- and *O*-sulfated-4-thio-HS analog (n=3 independently prepared samples examined over n=3 independent tests, shown in inset table; bars depict averaged data +/- standard deviation; Representative raw MRM results are shown in Supplementary Fig. 51).

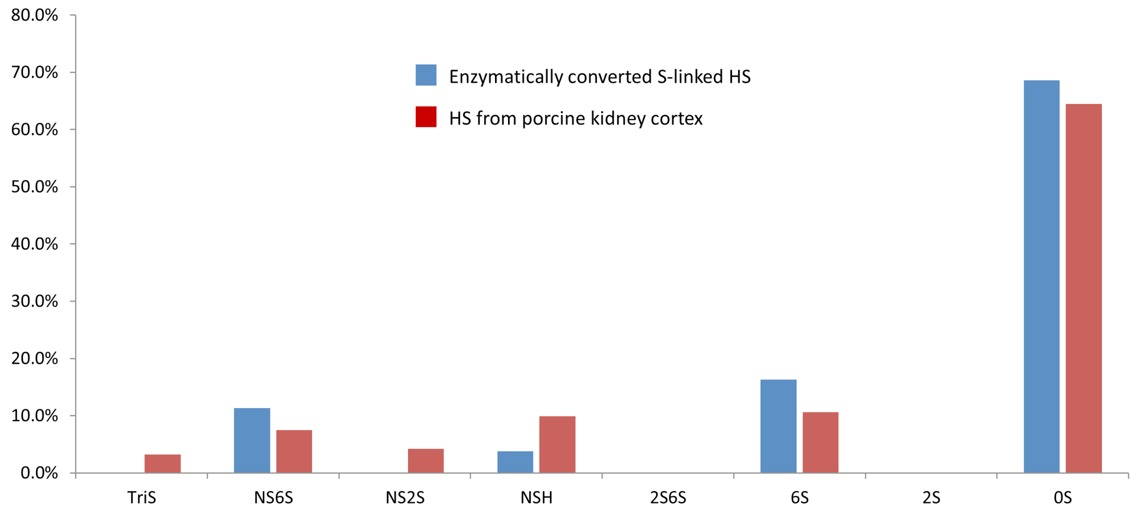

Supplementary Fig. 53. Disaccharide composition of enzymatically synthesized *S*-linked HS analog compared to a natural HS extracted from porcine kidney cortex[38].

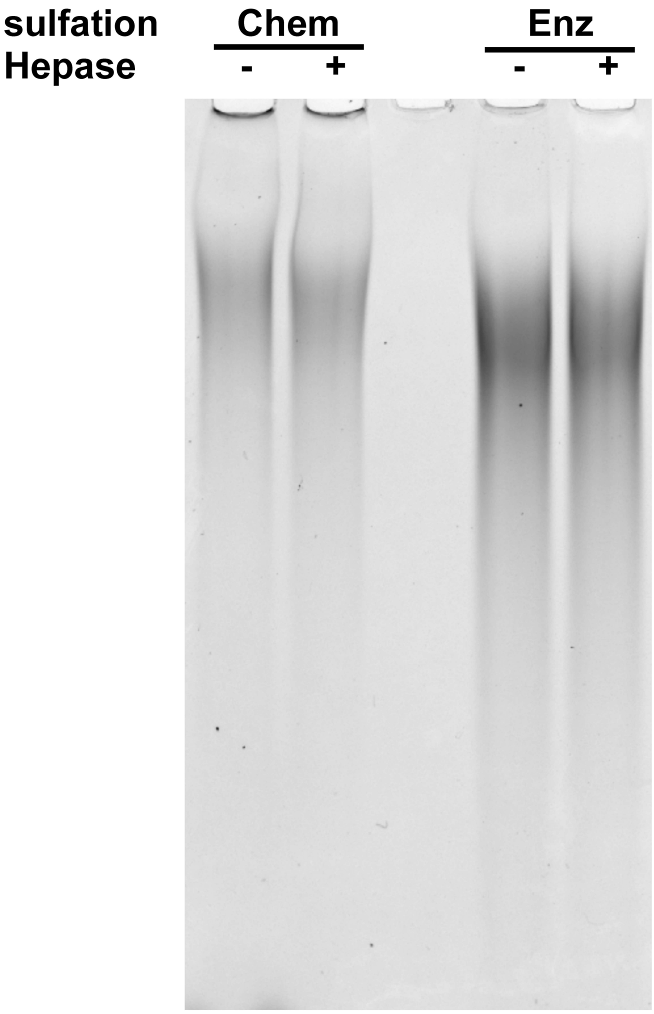

Supplementary Fig. 54. Human heparanase (***Hepase***) challenge of chemically (***Chem***) or enzymatically (***Enz***) sulfated *S*-linked polysaccharides.

PAGE analysis (20% gel with Stains-All detection) demonstrates that both sulfated versions of the hemi-A *S*-linked structure were recalcitrant to the action of heparanase even after 24 hours of digestion (*n*=1; also see Fig. 4b for independent heparanase challenge). In contrast, *O*-linked polymers were cleaved in minutes with only ~10% of the enzyme (*see* Main Fig. 4c, Supplementary Fig. 55). The enzymatically sulfated chains have higher sulfation levels than the chemically prepared chains, therefore, possess a higher charge density and migrate faster on gels.

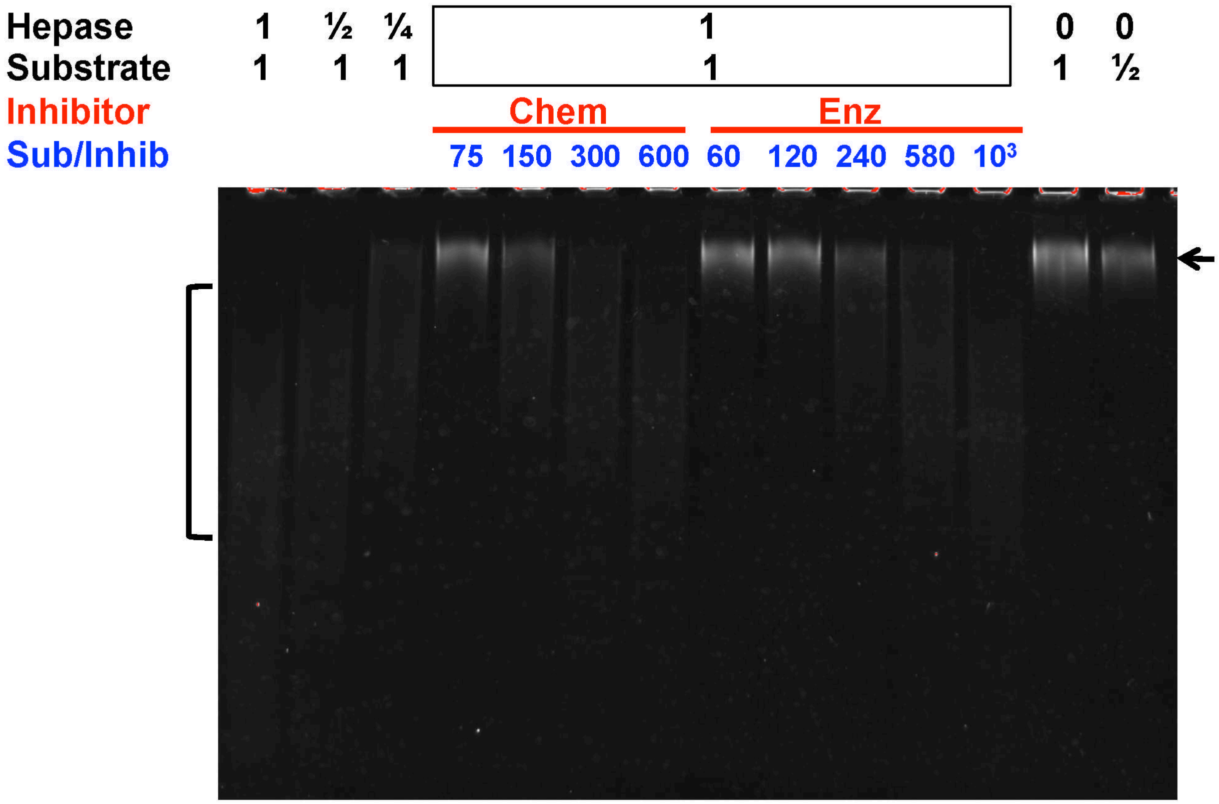

Supplementary Fig. 55. Inhibition of human heparanase by sulfated *S*-linked analogs.

PAGE analysis of the kinetics of heparanase enzyme (**Hepase**; load 1 = 0.12 pmoles) cleavage of a fluorescent sulfo-*O*-linked heparosan substrate (load 1 = 3.3 pmoles; marked with *arrow*) with a titration of the *S*-linked analog inhibitor (either chemically or enzymatically sulfated polymer, decreasing concentrations from left to right; 45-min time point shown here; 20 min in Fig. 4c). *Controls*: (*i*) reactions with one quarter or one half of the enzyme (*left lanes*; the *bracket* denotes the approximate extent of ½X enzyme fragments); (*ii*) reaction with half of the substrate without enzyme (*rightmost lane*).

The digestion of the sulfated heparosan with natural *O*-linkages was strongly inhibited by both *S*-linked sulfated analogs; the uncleavable polymers are at least ~200- to 300-fold more effective than expected for a simple competitive inhibitor mimicking the substrate as measured by the IC_{50} (~5-20 nM).

a

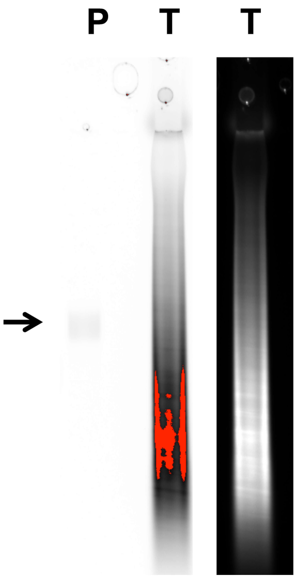

b

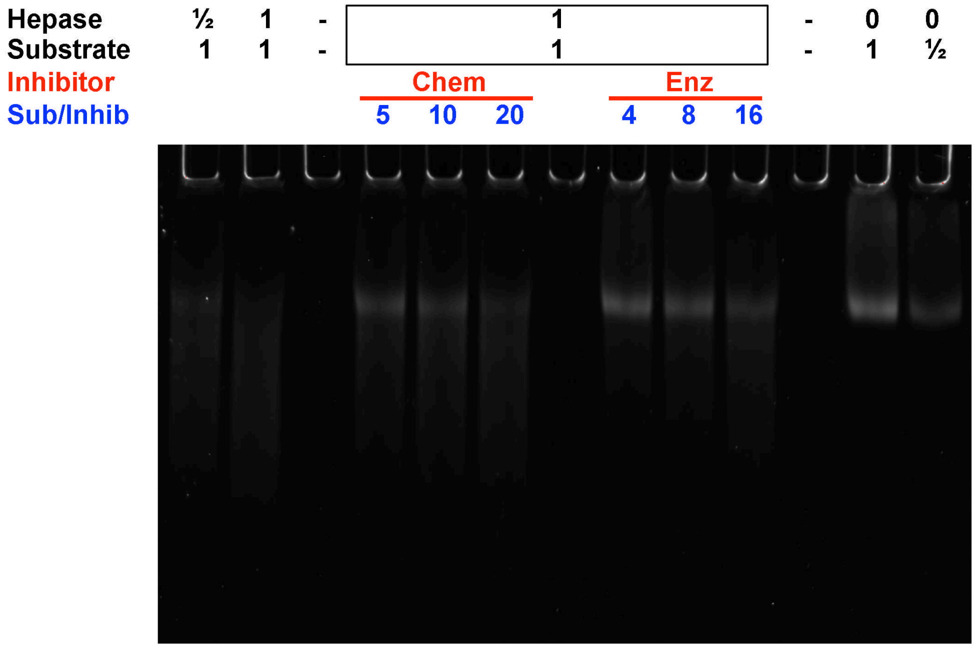

Supplementary Fig. 56. Inhibition of human heparanase digestion of a natural heparan sulfate substrate by sulfated *S*-linked heparan sulfate analogs.

a. PAGE analysis of the HS substrate used for heparanase inhibition tests. A purified fraction (**P**, marked with *arrow*) derived from a rhodamine-tagged natural heparan sulfate preparation (total, **T**) representing a large MW, abundant species in the broad-running smear was obtained by preparative electrophoresis (*see* Methods); the fluorescence signal of the analytical

gel is shown. The left two lanes were black/white inverted for more facile viewing, and a portion of the total HS lane in this image was saturated (red pixels); the right lane is the same lane of total HS imaged in the linear range in normal fluorescence mode (not inverted). 'P' was used as the substrate in kinetic assays.

b. Both chemically (**Chem**) or enzymatically (**Enz**) sulfated polymer could inhibit the degradation of the HS 'P' substrate by human heparanase at various substrate to inhibitor ratios (*Sub/Inhib*) at the 25 min time-point (1 of 2 gels; 3 titrations performed).

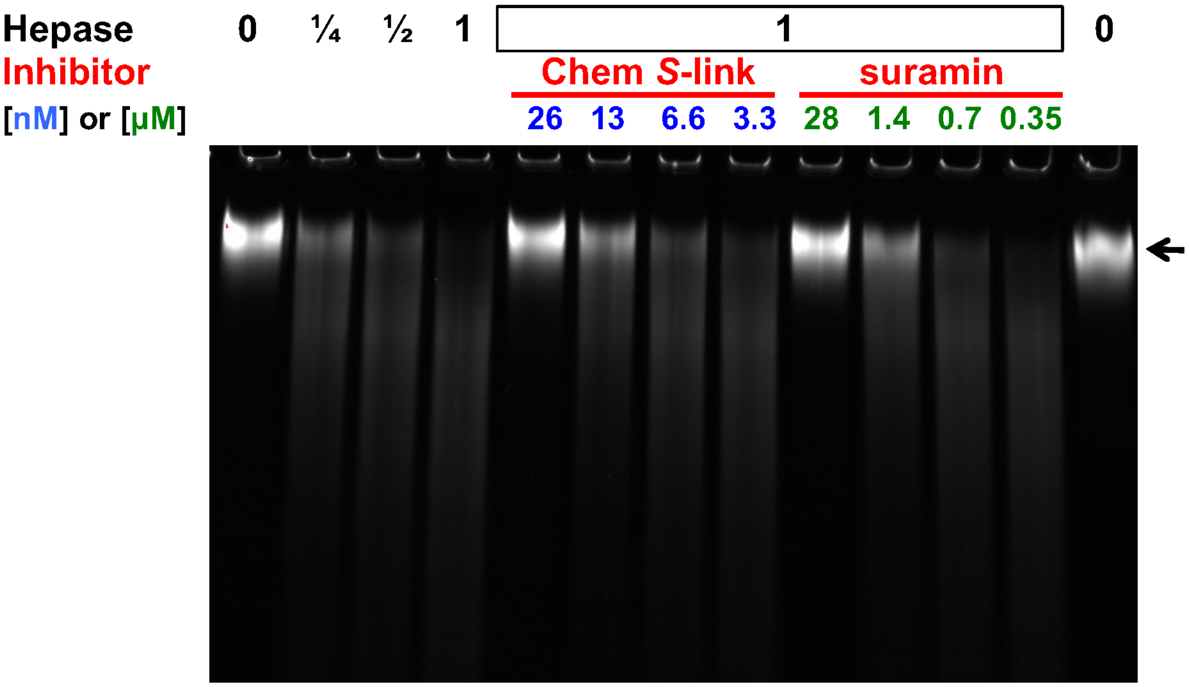

Supplementary Fig. 57. Inhibition of human heparanase digestion of a fluorescent, chemically sulfated *O*-linked heparosan substrate by the chemically sulfated hemi-A *S*-linked analog and suramin.

In this titration assay, the *S*-linked polysaccharide (shown in nanomolar units; one of 3 titrations, each with 2 time points) is at least 100-fold better than the known inhibitor suramin (in micromolar units) based on relative IC_{50} values (average ~5 versus ~550 nM, respectively).

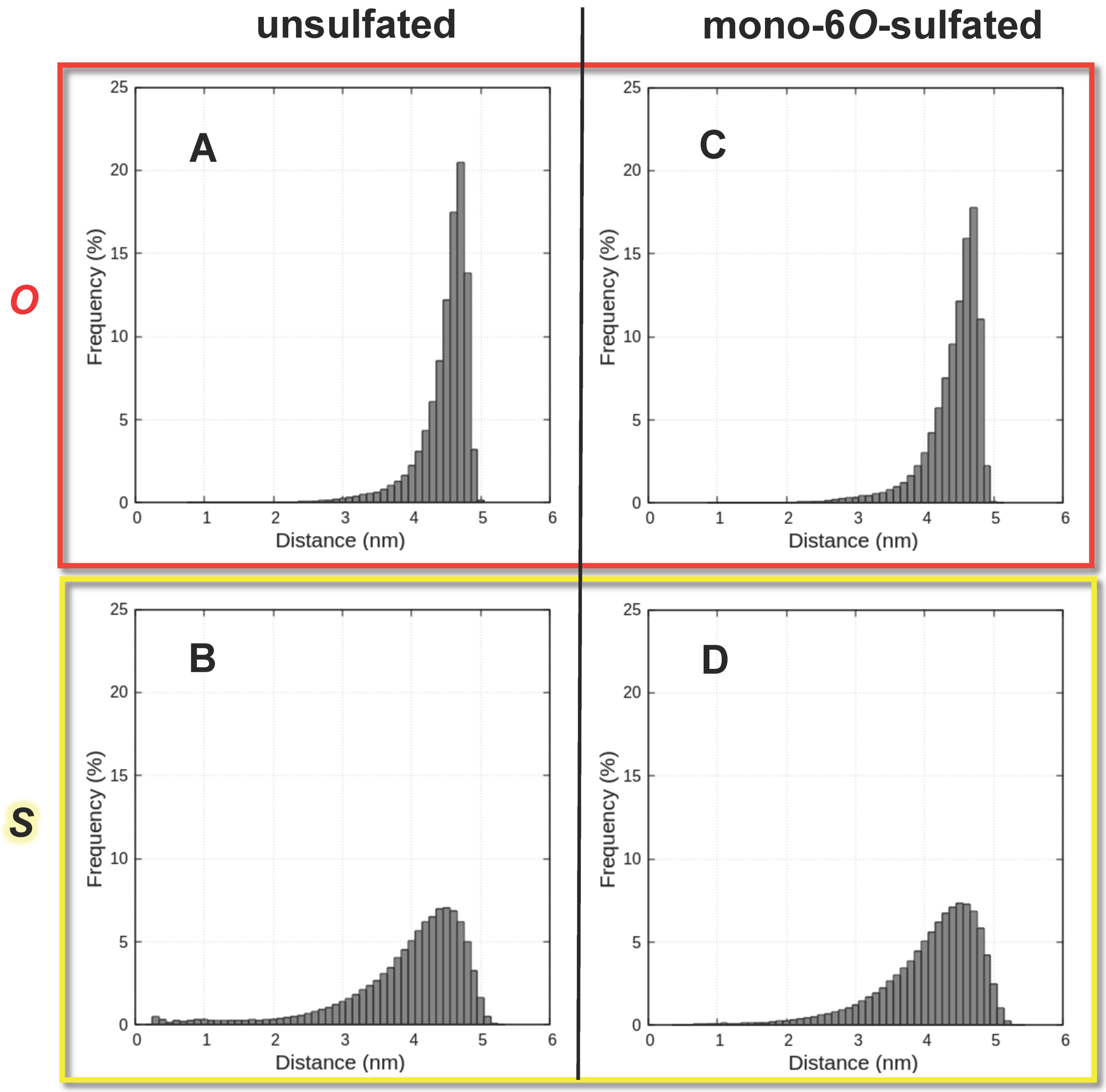

Supplementary Fig. 58. Molecular dynamics predictions of the distribution of end-to-end chain lengths for various heparosan-based decasaccharides.

The histograms depict the theoretical frequency (as a percentage of the total) of sugar chains possessing different average lengths in fully solvated 5 μs molecular dynamics simulations (end-to-end distance is from reducing terminal sugar O1 to non-reducing terminal sugar O4). Polymers are:

A. unsulfated, all *O*-links.
B. unsulfated, hemi-A *S*-links.
C. single 6*O*-sulfated GlcNAc in the middle of decamer, all *O*-links.
D. single 6*O*-sulfated GlcNAc in the middle of decamer, hemi-A *S*-links

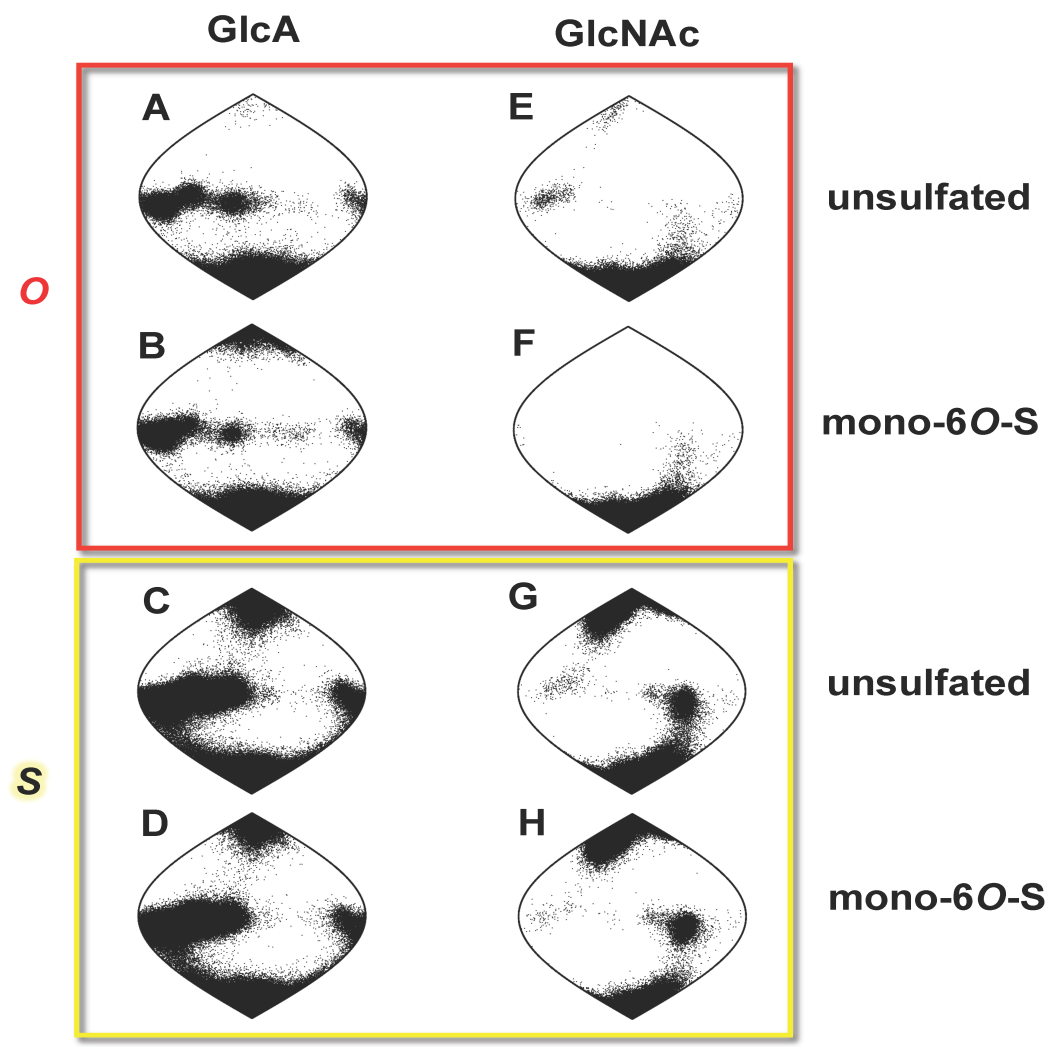

Supplementary Fig. 59. Sinusoidal plots of molecular dynamics simulations predictions of the sugar ring pucker conformers of the internal GlcA or GlcNAc residues in various heparosan-based decasaccharide polymers.

The sinusoidal plots depict the predicted sugar ring pucker conformer based on the Cremer-Pople azimuthal (θ) and meridional (φ) angles (*see* Main text). An internal monosaccharide (as noted in A-H below) from the various decasaccharide models is shown. The quantification of the various hypothetical boat conformers is presented in Supplementary Tables 1-4.

A. GlcA in an unsulfated *O*-linked polymer.
B. GlcA with a single adjacent 6-sulfated GlcNAc in an *O*-linked polymer.
C. GlcA in an unsulfated hemi-A *S*-linked polymer.
D. GlcA with a single adjacent 6-sulfated GlcNAc in hemi-A *S*-linked polymer.
E. GlcNAc in an unsulfated *O*-linked polymer.
F. 6-sulfated GlcNAc in an *O*-linked polymer.
G. GlcNAc in an unsulfated hemi-A *S*-linked polymer.
H. 6-sulfated GlcNAc in hemi-A *S*-linked polymer.

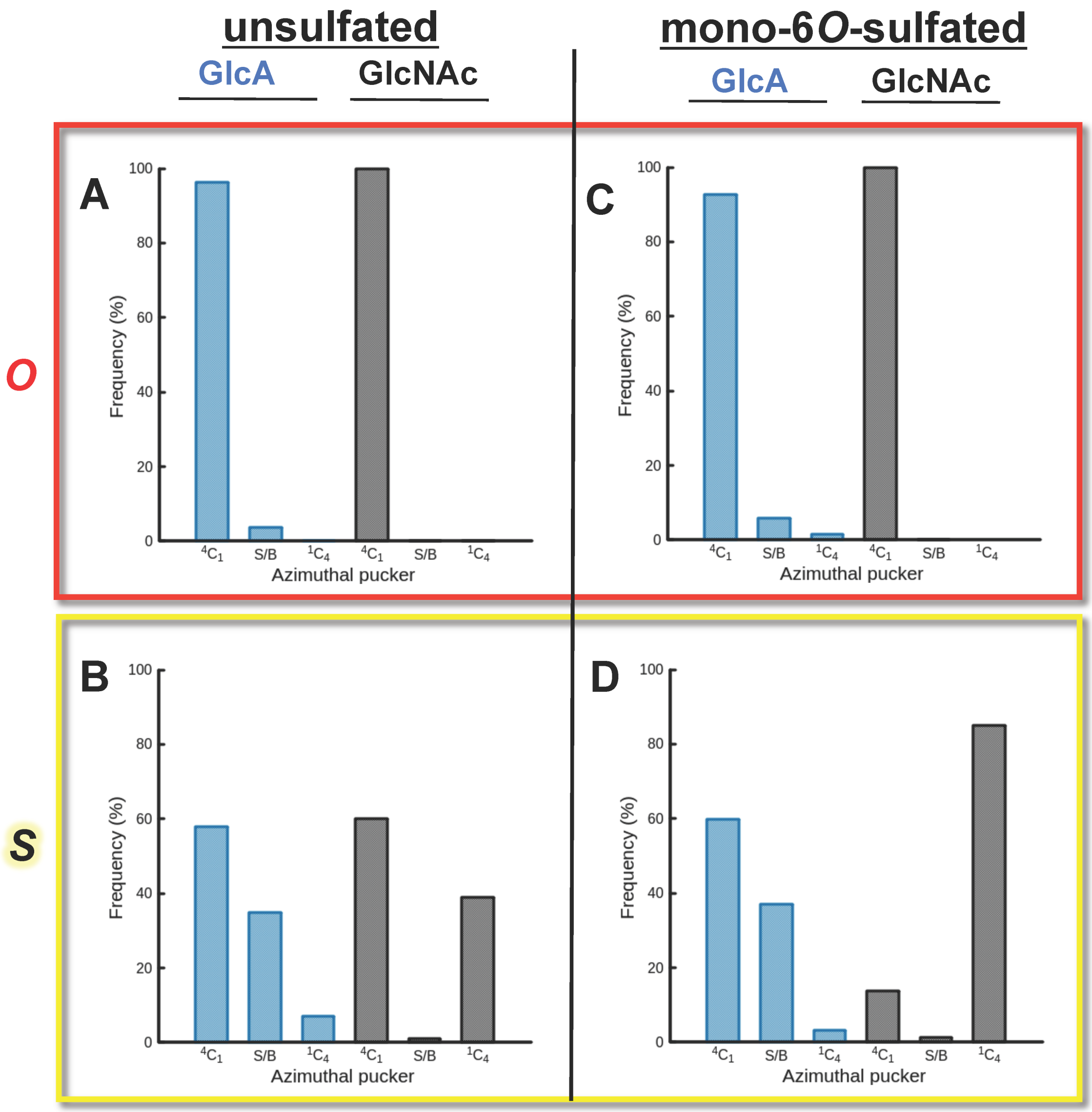

Supplementary Fig. 60. Quantification of azimuthal sugar ring pucker (indicating various chair, boat, skew conformers) of the internal glucuronic acid (GlcA) residue from multi-μs molecular dynamics simulations of various heparosan-based dodecamers.

The plots depict histograms of the predicted Cremer-Pople azimuthal puckering angles (60° intervals) of the GlcA (*blue*) or GlcNAc (*grey*) residue in the most internal disaccharide in 5 μs molecular dynamics simulations of various decasaccharides. These histograms permit a rough resolution between the two chairs in the northern and southern hemispheres (${}^{4}C_{1}$ and ${}^{1}C_{4}$) and prevalent skew and boat forms at the equator (sum of ${}^{1}S_{5}$, ${}^{1}S_{3}$, ${}^{1,4}B$, $B_{2,5}$ = ***S/B***).

A. unsulfated, all *O*-links.
B. unsulfated, hemi-A *S*-links.
C. single 6-sulfated GlcNAc in the middle of an *O*-linked chain.
D. single 6-sulfated GlcNAc in the middle of a hemi-A *S*-linked chain.

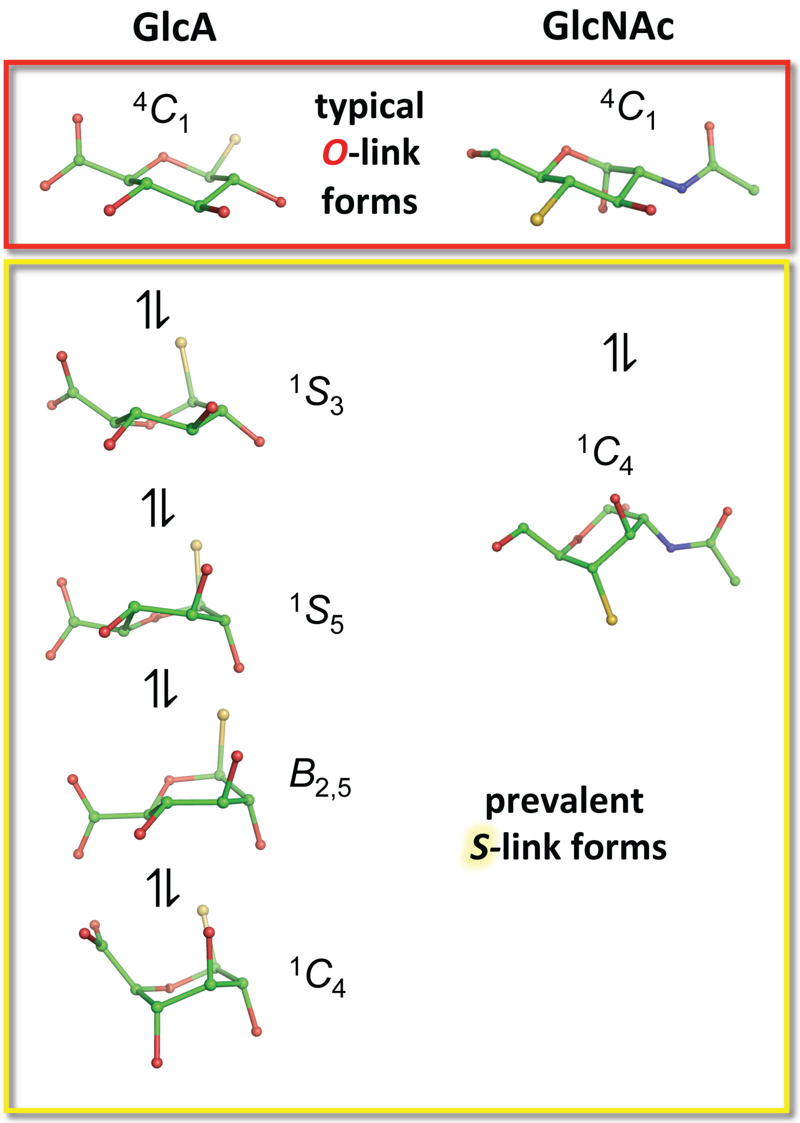

Supplementary Fig. 61. Prevalent hypothetical sugar ring pucker conformers extracted from multi-µs molecular dynamics simulations of various *O*-link and *S*-link heparosan-based decasaccharides.

Various predicted conformers of the GlcA (*left*) or GlcNAc (*right*) rings in the interior of decamers are shown in the 3D stick models. The most abundant forms found in *O*-link chains (*top*, *red box*) and the altered hemi-A *S*-link forms (*bottom*, *yellow box*) are depicted. (Atoms: carbon, *green*; oxygen, *red*; sulfur, *yellow*; nitrogen, *blue*)

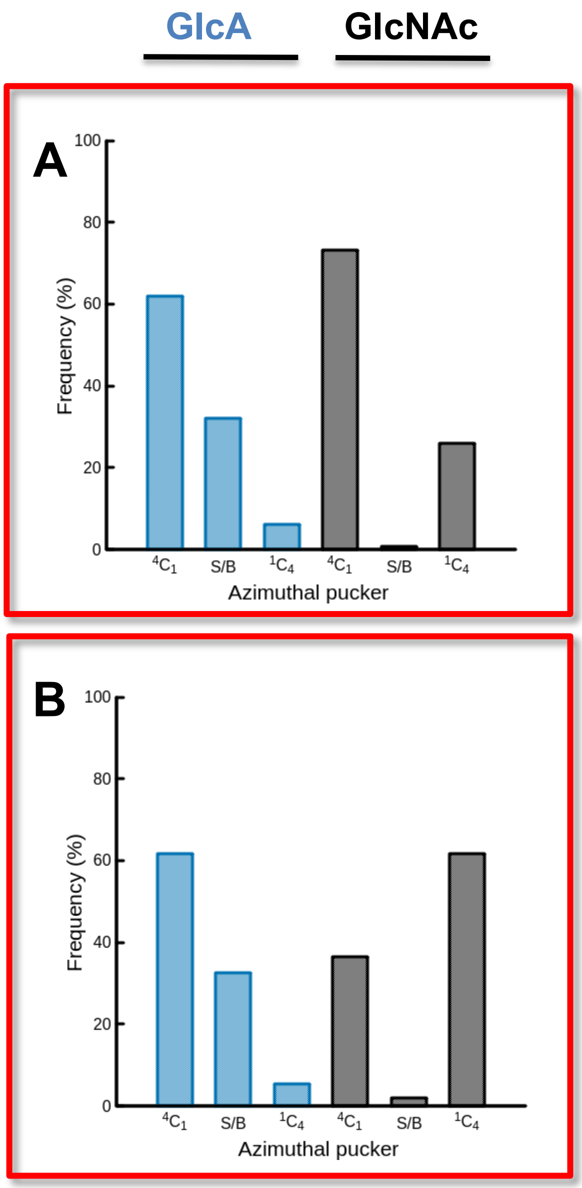

Supplementary Fig. 62. Quantification of azimuthal sugar ring pucker of the internal GlcA and GlcNAc residues from multi-µs molecular dynamics simulations of heparosan-based, hemi-A *S*-linked pentamers.

The plots depict histograms of the Cremer-Pople azimuthal puckering angles of a GlcA (*blue*) or GlcNAc (*grey*) residue in an internal disaccharide (marked with underlining) in 5 µs molecular dynamics simulations of a pair of alternative pentamers: **A**, non-reducing end GlcA-(6-*O*S-GlcNAc)-GlcA-GlcNAc-GlcA, or **B**, GlcNAc-GlcA-(6-*O*S-GlcNAc)-GlcA-GlcNAc. These histograms permit a rough resolution between the two chairs in the northern and southern hemispheres (***$^{4}C_{1}$*** and ***$^{1}C_{4}$***) and prevalent skew and boat forms at the equator (sum of $^{1}S_{5}$, $^{1}S_{3}$, $^{1,4}B$, $B_{2,5}$ = ***S/B***). The predicted conformer distributions of the *S*-linked pentamers and the *S*-linked decamers (Supplementary Fig. 60) are very similar.

Supplementary Tables 1-4. Quantification of the internal glucuronic acid (GlcA) meridional ring pucker.

The pucker (*i.e.* indicating types of boat, skew conformers) was modeled with multi-μs molecular dynamics simulations of various heparosan-based polymers (~500,000 data points each). Pucker is defined by the meridional angle (φ) only when the azimuthal angle is coincidentally within the range $-0.25 < \cos\theta < 0.25$, which roughly identifies boats and skew boats. Percentages are calculated using a denominator that is the total number of puckers measured (for any value of θ, including chairs). The boat and skew boat conformers are characterized in bins of 30° around the equator with the ^{03}B conformer occupying the φ=0° position. Data shown are for the internal GlcA residue from decasaccharide chains with:

A. unsulfated, all *O*-links.
B. adjacent single 6-sulfated GlcNAc in the middle of an all *O*-linked chain.
C. unsulfated, hemi-A *S*-links.
D. adjacent single 6-sulfated GlcNAc in the middle of a hemi-A *S*-linked chain.

The GlcA residue in *S*-linked chains is often in a distorted state in comparison to the same sugar in *O*-linked chains as seen by the increased frequency in some pucker conformers.

Supplementary Table 1 (*O*-link)

Pucker	^{03}B	$^{3}S_1$	B_{14}	$^{5}S_1$	^{25}B	$^{2}S_0$	B_{03}	$^{1}S_3$	^{14}B	$^{1}S_5$	B_{25}	$^{0}S_2$
%	0.09	0.02	0.01	0.01	0.00	0.06	0.25	1.35	0.67	0.44	0.14	0.37

Supplementary Table 2 (*O*-link, sulfated disaccharide)

Pucker	^{03}B	$^{3}S_1$	B_{14}	$^{5}S_1$	^{25}B	$^{2}S_0$	B_{03}	$^{1}S_3$	^{14}B	$^{1}S_5$	B_{25}	$^{0}S_2$
%	0.04	0.02	0.02	0.01	0.01	0.09	0.55	2.82	1.42	0.27	0.08	0.19

Supplementary Table 3 (*S*-link)

Pucker	^{03}B	$^{3}S_1$	B_{14}	$^{5}S_1$	^{25}B	$^{2}S_0$	B_{03}	$^{1}S_3$	^{14}B	$^{1}S_5$	B_{25}	$^{0}S_2$
%	0.21	0.03	0.00	0.00	0.07	0.56	1.08	4.88	6.02	10.10	5.32	2.70

Supplementary Table 4 (*S*-link, sulfated disaccharide)

Pucker	^{03}B	$^{3}S_1$	B_{14}	$^{5}S_1$	^{25}B	$^{2}S_0$	B_{03}	$^{1}S_3$	^{14}B	$^{1}S_5$	B_{25}	$^{0}S_2$
%	0.13	0.01	0.00	0.00	0.06	0.39	1.18	4.60	4.91	14.03	6.11	2.14
